# Effectiveness of physical therapies for patients with knee osteoarthritis: a systematic review and network meta-analysis of randomized controlled trials

**DOI:** 10.3389/fmed.2025.1714912

**Published:** 2025-12-04

**Authors:** Shanshan Zhang, Yihao Ge, Zhaodong Bi, Yuqing Li, Cici Bai, Feng Zhang, Miao Tian, Yunxu Tian, Kexin Zhang, Xiuting Li, Yanbin Zhu

**Affiliations:** 1 Department of Orthopaedic Surgery, The Third Hospital of Hebei Medical University, Shijiazhuang, China; 2 Department of Rehabilitation, The Third Hospital of Hebei Medical University, Shijiazhuang, China; 3 Department of Rehabilitation Medicine, Shanghai Jiao Tong University Affiliated Sixth People’s Hospital, Shanghai, China; 4 Key Laboratory of Biomechanics of Hebei Province, Hebei Orthopedic Research Institute, The Third Hospital of Hebei Medical University, Shijiazhuang, China

**Keywords:** physical therapies, knee osteoarthritis, pain, network meta-analysis, systematic review

## Abstract

**Background:**

Physical therapy offers a useful option for management of knee osteoarthritis (KOA) and this study aims to summarize the latest evidence on the effectiveness of variable physical therapies.

**Methods:**

We considered randomized trials of patients with KOA that compared any of the following interventions for treatment of osteoarthritis pain: physical therapies, general nursing, placebo. We performed frequentist random-effect network meta-analyses to summarize the evidence and applied the Confidence in Network Meta-Analysis frameworks to rate the certainty of evidence, calculate the treatment effects, categorize interventions, and present the findings.

**Results:**

The search identified 74 eligible RCTs, enrolling 3,707 participants. TENS demonstrated the largest reduction of NRS (MD, −2.73; 95% CI, −5.20 to −0.26) and VAS pain at rest (MD, −2.87; 95% CI, −4.87 to −0.87). Moderate to low-level evidence established laser as most effective for improving VAS for pain during walking at <1 month (MD, −2.50; 95% CI, −3.88 to −1.11) and 1–3 months (MD, −2.49; 95% CI, −4.51 to −0.48), and neuromuscular exercise (MD, −1.77; 95% CI, −3.14 to −0.39) at 3 months. For WOMAC total scores at <1 month, 1–3 months, and >3 months, shockwave (MD, −9.56; 95% CI, −16.71 to −2.41; low-level confidence), aquatic sports (MD, −16.53; 95% CI, −30.74 to −2.31; low-level confidence), and shockwave (MD, −30.02; 95% CI, −40.39 to −19.65, moderate-level confidence) respectively, demonstrated the greatest improvements.

**Conclusion:**

Physical therapies exhibited varying efficacy profiles in management of KOA with most being supported by moderate-to-low levels evidence, warranting further studies to better establish their effectiveness.

**Systematic review registration:**

Identifier PROSPERO CRD42023458296.

## Introduction

Knee osteoarthritis (KOA) is a chronic progressive disease with various causes, affecting over 600 million individuals globally (1), and associated with substantial disease burden due to joint pain, stiffness, functional decline and even disability (2). In the early stages of KOA, lifestyle interventions (e.g., weight loss, smoking cessation, and dietary changes) or pharmacotherapy (e.g., non-steroidal anti-inflammatory drugs, or opioids) are typically the preferred management approaches (3). However, issues with patient compliance or the risk of adverse events with these treatments may be significant concerns (4, 5). Additionally, the efficacy of the emerging platelet-rich plasma injection therapy and the widely used arthritis supplements, such as chondroitin sulfate and glucosamine, remains uncertain (6, 7). Physical therapy is generally safe, non-invasive and low-tech, making it a potentially promising alternative or adjunct therapy for KOA management.

The 2019 update to the American College of Rheumatology (ACR) guidelines strongly recommends exercise for patients with knee osteoarthritis, based on a 70% consensus among voting group members; however, existing evidence is insufficient to recommend specific exercise prescriptions (8). In contrast, the 2022 guidelines from the American Academy of Orthopedic Surgeons (AAOS) in 2022 took a more limited attitudes toward physical therapies such as ultrasound, laser, Transcutaneous Electrical Neuromuscular Stimulation (TENS), based on the low research quality, limited quantity, unclear balance between benefits and hazards (9). In 2009 and 2016, Cochrane conducted systematic reviews and meta-analyses, comparing the efficacy and safety of therapies such as TENS and aquatic sports, suggesting that aquatic sports may have a small, short-term effect on pain with moderate-quality evidence, but, the efficacy of TENS has yet to be determined (10, 11). Since then, numerous trials have been published for some novel physical therapies, including ultrasound (12), neuromuscular exercise (13), and electroacupuncture (14), yielding results that could potentially change clinical practice. However, their effectiveness has not been systematically reviewed, and the relative merits of existing physical therapies remains undetermined.

We conducted a systematic review and network meta-analysis (NMA) of available randomized controlled trials (RCTs) to summarize the latest evidence on the pain-relieving effect and safety of physical therapies in patients with KOA.

## Methods

The protocol for this study was registered with PROSPERO. This study followed the Preferred Reporting Items for Systematic Reviews and Meta-Analyses (PRISMA, Supplementary materials 1, 2) 2020 (checklist) (15).

### Search strategy and selection criteria

Two researchers searched PubMed, Embase, the Cochrane Library, and Web of Science for RCTs of physical therapy in people with knee osteoarthritis from database inception to December 15, 2024, which utilized a combination of keywords and free text words, was initially developed for PubMed and then adapted for the other three databases. There are no restrictions on language or age. In addition, references of included articles and relevant systematic reviews were screened for potentially eligible studies (Appendix 1).

We included RCTs of patients with KOA that compared any of the following physical therapy interventions for osteoarthritis pain: electroacupuncture, laser, TENS, ultrashort wave, interference therapy, ultrasound, shockwave, aerobic exercise, strength training, neuromuscular exercise, aquatic sports, balance training and proprioceptive exercise. The comparator can be placebo, general nursing, or any alternative physical therapy. The treatment duration had to be a minimum of 1 week, and pain had to be measured using at least one of the following scales or subscales: Numeric Rating Scale (NRS), Visual Analog Scale (VAS), or Western Ontario and McMaster Universities Osteoarthritis Index (WOMAC). Studies involving joint cavity perfusion, oral medication, surgical treatment, combination therapy research, conference papers, and incomplete raw data were excluded.

The literature retrieved from the database was imported into Endnote X21, and duplicates were removed. The screening process involved three stages. Firstly, eligible studies were independently screened by two reviewers based on title and abstract, with any uncertain studies included for further review. Secondly, articles potential meeting the criteria underwent a full-text assessment using predetermined inclusion and exclusion criteria. Finally, a thorough examination of previously published reviews and references cited in the literature was conducted. Any discrepancies were resolved through consultation.

### Data extraction and analysis

A pre-designed table was used to extract detailed information for each eligible study (Appendix 2), encompassing study characteristics (i.e., country, journal, and publication year), population demographics (i.e., baseline age, gender, and body mass index (BMI)), and interventions (i.e., name, follow-up duration, and outcomes assessed). We also evaluated the safety of physical therapy by analyzing adverse events without predefined restrictions. These data covered treatment discontinuations due to adverse events, incidences of increased knee pain, swelling, stiffness, fall, nausea, and other relevant outcomes. In case of incomplete data, efforts were made to contact the corresponding author. Any discrepancies were resolved through discussion.

We formed a multidisciplinary rehabilitation team comprised of one arthrologist, two rehabilitation physicians, two physiotherapists, two rheumatologists, two pharmacists and three nurses. Following group discussion, we prioritized the following outcomes as crucial—VAS, NRS and WOMAC total scale; and as important but not crucial—WOMAC individual subscales (i.e., pain, stiffness, function), and adverse events. We converted the means and standard deviations of VAS outcomes from millimeters to centimeters and standardized the WOMAC scale to a 96-point scale. Changes in mean and standard deviation from baseline for VAS, NRS, and WOMAC were calculated, and effects from individual trials were expressed as mean difference (MD) with a 95% confidence interval (CI). Assessments were conducted at distinct time periods following treatment allocation: <1 month, 1–3 months, >3 months. Each study’s outcome was used only once. When multiple studies evaluated the same intervention at different dose levels, each dose level was analyzed as a separate subgroup. However, if the dose level was not reported, or when the reported doses were comparable and could not be meaningfully distinguished, the data were combined into a single intervention group.

For studies reporting pre- and post-intervention means and standard deviations (SDs), the mean change and SD of the change were calculated using the standard formula, where r = 0.5 (16). If standard error (SE) or confidence interval (CI) were provided, SDendpoint was calculated using the Review Manager (Revman 5.4). For studies only reporting median, range, and interquartile range, we converted these values to standard deviation (SD) using an online conversion tool.^1^


Mean change=Mean endpoint–Mean baseline

$$\mathrm{SD}\;\text{change}=\sqrt{\begin{array}{l} {\left(\text{SDbaseline}\right)}^{2}+{\left(\text{SDendpoint}\right)}^{2}-2\times r\\ \times \text{SDbaseline}\times \text{SDendpoint} \end{array}}$$

The minimal clinically important difference (MCID) is defined as the smallest amount by which an outcome must change (mean difference or standard mean difference) to be considered clinically significant (17). The results were deemed to be clinically meaningful only if they met the clinical significance and statistical significance (18). The MCIDs for NRS, VAS, and the WOMAC subscales for pain, stiffness, function, and total score are 1.8 (19), 1.8 (20), 2 (21), 0.76 (22), 6 (20), 6.7 (23), respectively.

Two reviewers independently evaluated the risk of bias of the included trials using the Cochrane Randomized Trial Bias Risk Tool (version 1.0) (Appendix 3) (24). The domains to be assessed included random sequence generation, allocation concealment, blinding of participants and personnel, blinding of outcome assessment, incomplete outcome data, selective reporting, and other biases. Risk of bias for each domain was categorized as “low,” “high,” or “unclear.”

### Assessing the confidence of evidence

The Confidence in Network Meta-Analysis (CINeMA) tool was employed to assess the confidence in the NMA (25, 26), which included six domains: within-study bias, reporting bias, indirectness, imprecision, heterogeneity, and inconsistency. Each domain was judged as having no concerns, some concerns, or major concerns. Serious or very serious concerns in any of these domains resulted in downgrading the quality of evidence by one to two levels and the final quality of evidence was categorized as high, medium, low, or very low (Appendix 4). Any discrepancies were resolved through consensus. The analysis was carried out using the Revman and CINeMA websites.^2^

### Statistical analysis

We conducted a NMA of RCTs using the Stata network command within the frequentist framework. MD were used for continuous outcomes such as VAS and NRS, while odds ratios were used for binary outcomes like adverse events. Random-effects models were chosen as they are considered the most appropriate and conservative approach to account for variability among RCTs, providing pooled estimates and 95% confidence intervals. We assessed heterogeneity with Cochran’ Q test and the τ^2^ values, categorizing them as low (<0.04), low-moderate (0.04–0.16), moderate-high (0.16–0.36), and high (>0.36) (27). In cases of high heterogeneity, sensitivity analysis was performed by excluding each included study one by one before conducting the network meta-analysis again. We also performed a sensitivity network meta-analysis with low risk of bias for the crucial outcomes. Subgroup analyses based on BMI, age, and sex were conducted if each subgroup contained at least 10 RCTs. The cut-points for these variables were determined *post hoc* based on the median values of the included participants (i.e., lower 50% vs. upper 50%). Global consistency was evaluated using a global test, and local inconsistency was assessed using node-splitting methods (Appendix 5). Transitivity was examined by comparing the similarity of included populations based on average age, gender distribution, BMI, baseline pain levels, and Kellgren Lawrence scores.

We used Stata 16.0 to create a network diagram and ranked different physical therapies based on surface under the cumulative ranking (SUCRA) to evaluate their efficacy in treating knee osteoarthritis, with larger values representing higher ranking probabilities, funnel plots were employed to evaluate the potential impact of small-scale studies when at least 10 trials were included in a comparison, with parallel Egger tests conducted (Appendices 6–9, Appendix 13) (28). A *p* value > 0.05 indicated no publication bias. No formal multiplicity adjustments were applied across outcomes or time windows. Interpretations and conclusions were primarily anchored to the CINeMA certainty assessment for the primary outcome domain.

## Results

### Literature selection and study characteristics

**Figure 1:**
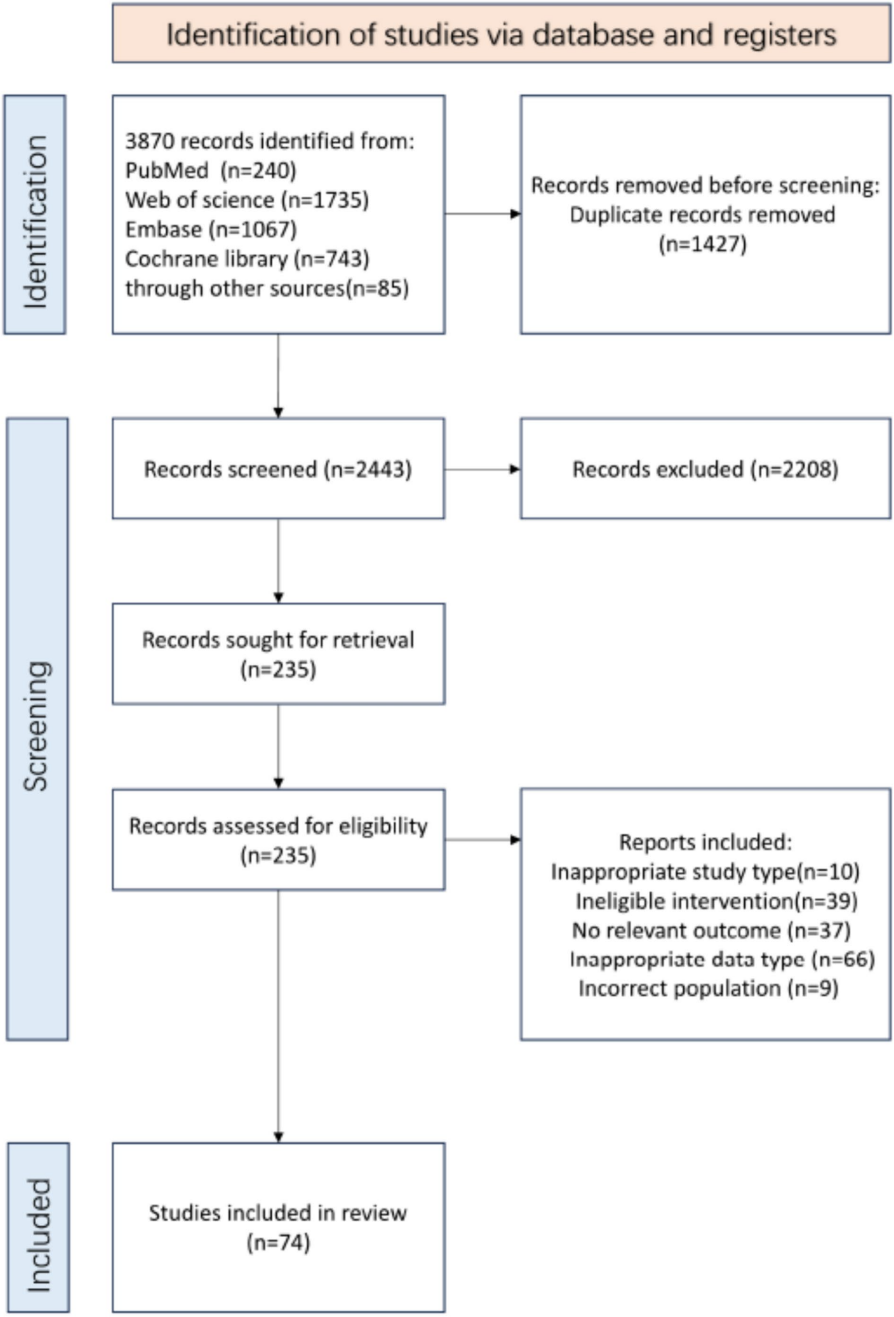
Flow diagram of preferred reporting items identified, included, and excluded for systematic reviews and meta-analyses (PRISMA).

A total of 3,870 articles were screened, and 235 full-text articles were evaluated (Figure 1) and updated the literature search once to ensure that the search results were up to date. Seven additional trials were identified from the reference lists of relevant papers and reviews. In total, we included 74 RCTs involving 3,707 patients, conducted across 21 countries and regions. The mean age of participants at enrollment was 62.1 years (SD = 7.8; age was not reported in three articles). Male participants constituted 22.4% and the mean BMI was 28.7 (SD = 5.2; BMI was not reported in 12 articles) (Appendix 2). Ultra short waves were not included due to the relevant trials not meeting the inclusion criteria, thirteen intervention measures were identified. Resistance training and strength training were merged and denoted as strength training; ultrasound and shockwave therapy as shockwave therapy; balance training and proprioception training as balance training; and interference therapy, electroacupuncture, and TENS as TENS. Tai Chi and Baduanjin were categorized as aerobic exercise, while health education and attention control were categorized as general nursing. Placebo conditions were defined as instances where the machine was not operational (23%) or did not register energy output (73%). General nursing is defined as health education (45%), attention control (10%), and maintaining existing daily activities (45%), rather than a true placebo. Consequently, there were nine interventions in the intervention group compared to the control group.

### Risk of bias, confidence of evidence, transitivity, and consistency

Out of 74 experiments, 26 studies (35%) were found to have a high risk of bias. Local inconsistencies were found during VAS pain during walking at more than 3 months and WOMAC total <1 month, but there is no strong statistical evidence to suggest global inconsistency. The study population was similar in terms of mean age, gender distribution, and BMI. However, only 26 studies (35%) have reported on baseline Kellgren Lawrence scores. After evaluating the level of evidence using CINeMA, most paired comparison results were found to have low-moderate credibility, the main reason for the downgrade is imprecision. The tau^2^ results showed high heterogeneity, except for VAS paint during walking at more than 3 months and WOMAC stiffness scores at more than 3 months. Additionally, we found asymmetric evidence in the funnel plots of VAS paint during walking at less than 1 month and WOMAC total<1 month.

### Crucial outcomes

**Figure 2:**
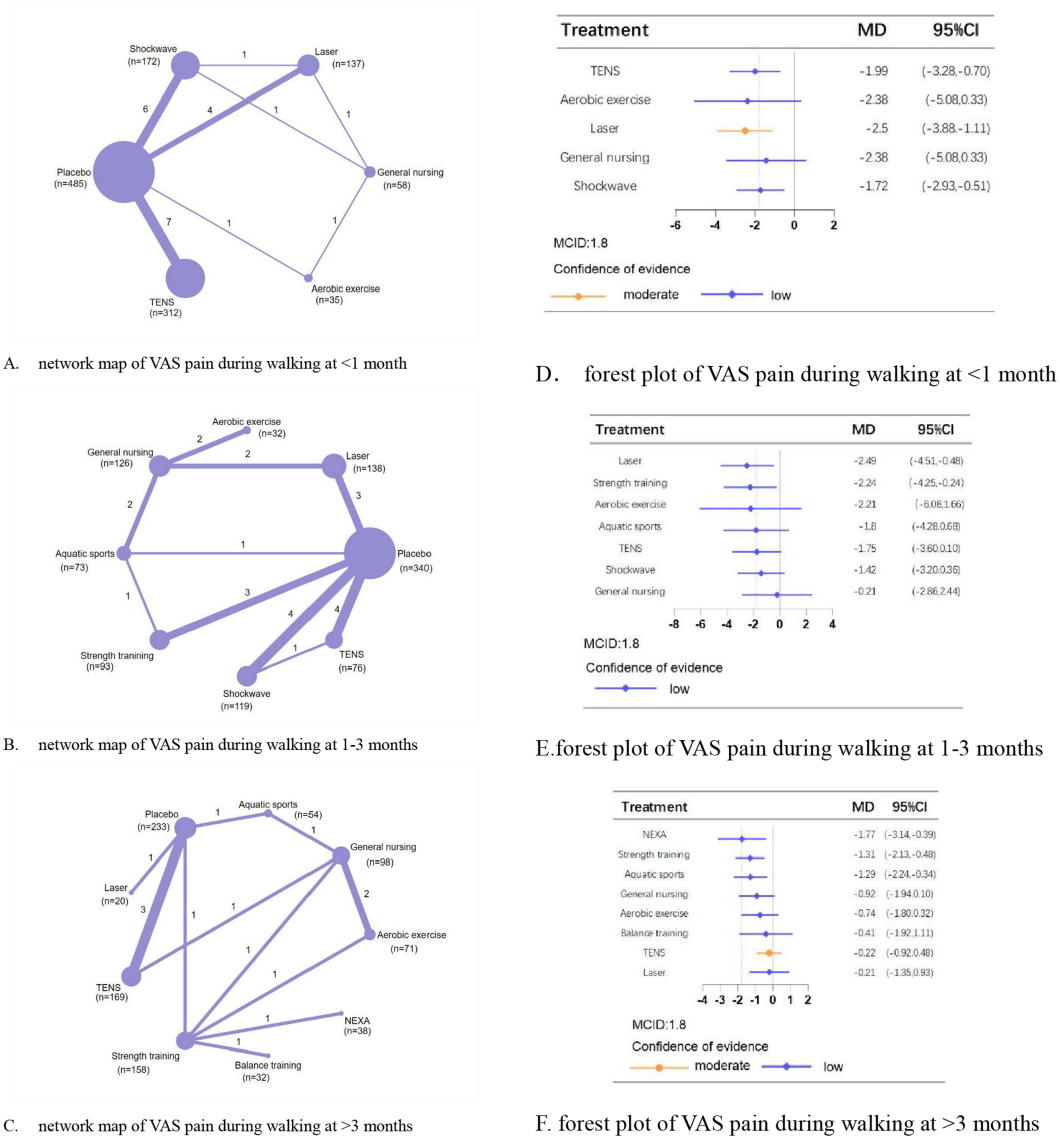
Network meta-analysis of different physical therapies for VAS pain during walking. **(A–C)** Network map of available comparisons between physical therapies and placebo. Each circle indicates a treatment node in the network map. The size of each node is proportional to the number of participants. Lines connecting two nodes represent direct comparisons between those two treatments. The thickness of each line is proportional to the number of trials directly comparing the two connected treatments. The thickness of the lines is proportional to the number of trials directly comparing the 2 connected treatments. **(D,E)** Forest plot represents the direct and indirect compared with placebo; The MCID of the VAS is indicated by the dashed line. Effect sizes are presented as MDs with 95% CI. Colors indicate the confidence in the effect estimates according to the CINeMA framework: orange = moderate, blue = low. MD, mean difference; MCID, minimal clinically important difference.

Regarding VAS pain during walking at <1 month, 1–3 months, and >3 months, we analyzed 22, 23, and 14 trials involving 1,244, 1,127, and 873 patients, respectively. The placebo-controlled effectiveness of physical therapies in addition to balance training and aerobic exercise were demonstrated. Laser (MD, −2.50; 95% CI, −3.88 to −1.11; moderate confidence), laser (MD, −2.49; 95% CI, −4.51 to −0.48; low confidence), and neuromuscular exercise (MD, −1.77; 95% CI, −3.14 to −0.39; low confidence) were most effective in improving pain at <1 month, 1–3 months, and >3 months, respectively (Figure 2).

**Figure 3:**
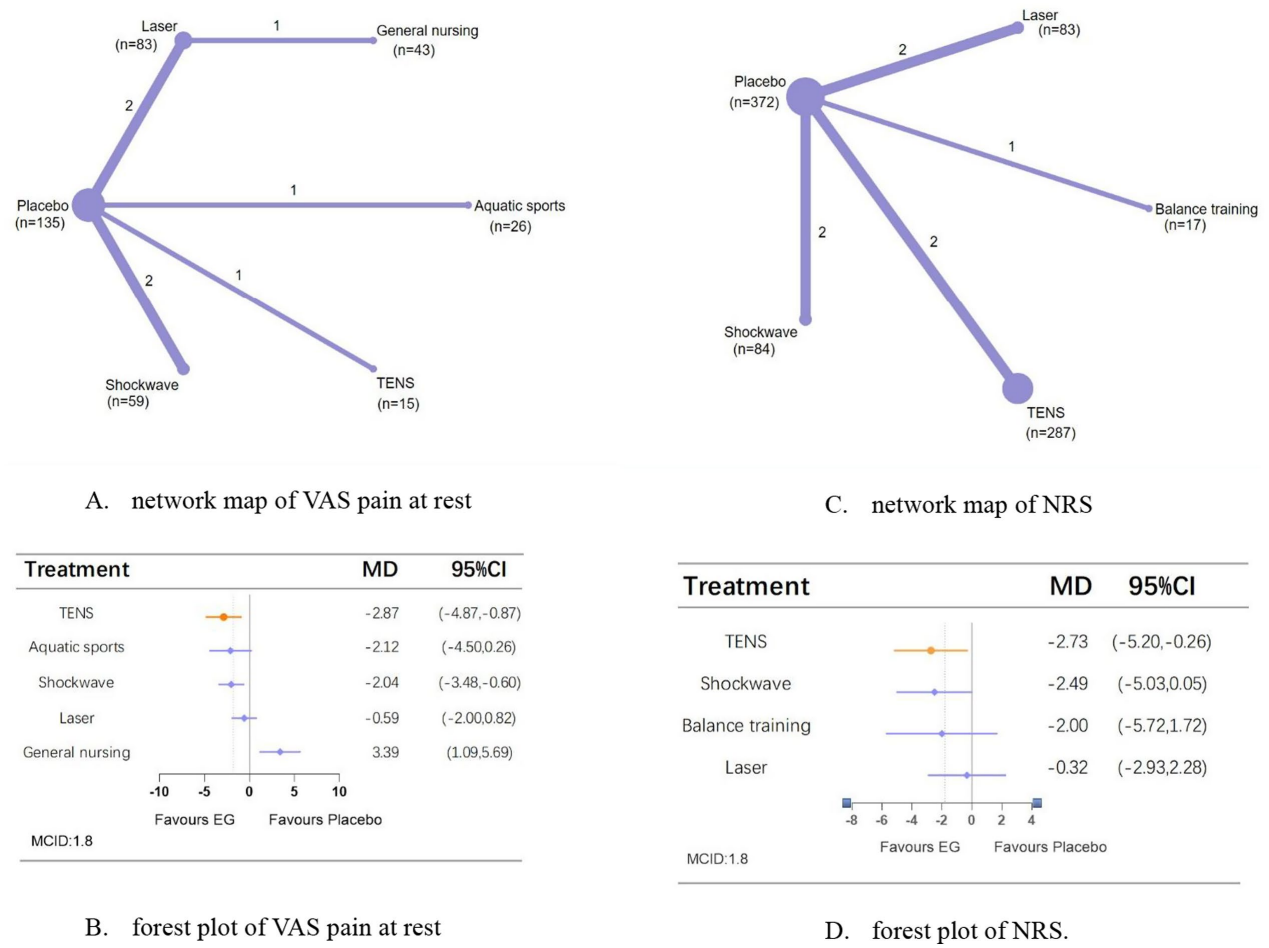
Network meta-analysis of different physical therapies for VAS pain at rest and NRS. **(A,B)** Network map of available comparisons between physical therapies and placebo. Each circle indicates a treatment node in the network map. The size of each node is proportional to the number of participants. Lines connecting two nodes represent direct comparisons between those two treatments. The thickness of each line is proportional to the number of trials directly comparing the two connected treatments. **(C,D)** Forest plot represents the direct and indirect compared with placebo; The MCID of the VAS, NRS are indicated by the dashed line. Effect sizes are presented as MDs with 95% CI. Colors indicate whether it has clinical significance: orange = yes, blue = no. MD, mean difference; MCID, minimal clinically important difference.

A network meta-analysis of VAS pain at rest included seven studies involving 361 patients (two studies employed independent and incomparable interventions) with an intervention period of 3–8 weeks. TENS (MD, −2.87; 95% CI, −4.87 to −0.87) showed the most significant analgesic effects compared with placebo (Figures 3A,B).

A network meta-analysis of NRS comprised 7 trials (one study with independent, non-comparable interventions), involved 843 people over an intervention period of 3–12 weeks. TENS (MD, −2.73; 95% CI, −5.20 to −0.26) is the only means that can improve pain compared with placebo (Figures 3C,D).

**Figure 4:**
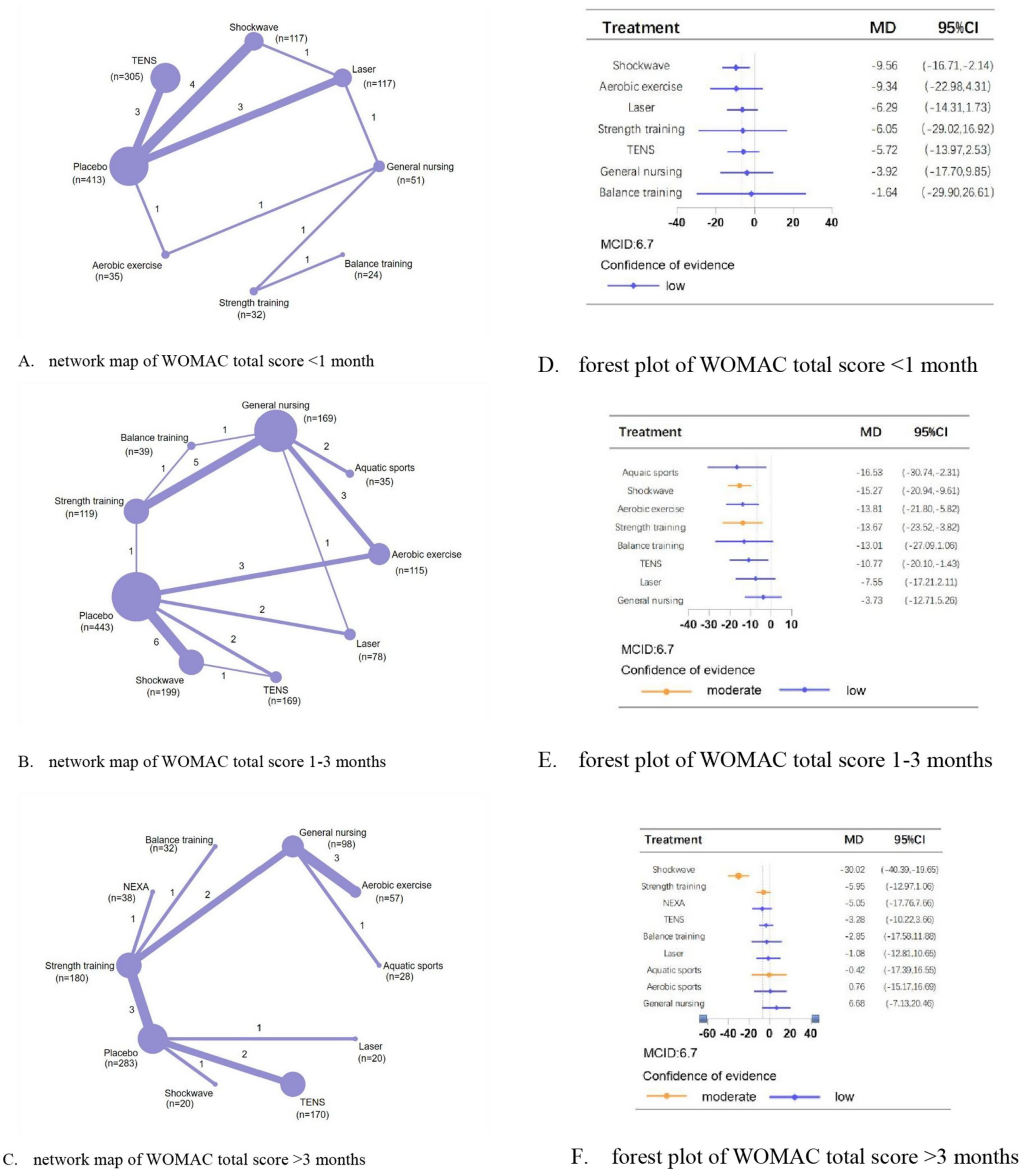
Network meta-analysis of different physical therapies for WOMAC total. **(A–C)** Network map of available comparisons between physical therapies and placebo. Each circle indicates a treatment node in the network map. The size of each node is proportional to the number of participants. Lines connecting two nodes represent direct comparisons between those two treatments. The thickness of each line is proportional to the number of trials directly comparing the two connected treatments. **(D,E)** Forest plot represents the direct and indirect compared with placebo; The MCID of the WOMAC is indicated by the dashed line. Effect sizes are presented as MDs with 95% CI. Colors indicate the confidence in the effect estimates according to the CINeMA framework: orange = moderate, blue = low. MD, mean difference; MCID, minimal clinically important difference.

For WOMAC total scores at <1 month, 1–3 months, and >3 months, we analyzed 16, 28, and 15 trials involving 1,094, 1,366, and 926 patients, respectively. Compared with placebo, shockwave，aquatic sports, and shockwave showed the most significant efficacy in reducing total scores at <1 month (MD, −9.56; 95% CI, −16.71 to −2.41; low confidence), 1–3 months (MD, −16.53; 95% CI, −30.74 to −2.31; low confidence), and >3 months (MD, −30.02; 95% CI, −40.39 to −19.65; moderate confidence), respectively (Figure 4).

In addition, a summary table (Table 1) provides an overview of the crucial outcomes, including effect estimates, confidence levels, and whether the MCID threshold was reached, to enhance the interpretability of the findings.

**Table 1:** Summary of crucial outcomes from the network meta-analysis.

Outcome measure	Intervention (vs. control)	Effect estimate (95% CI)	Confidence level (CINeMA)	MCID threshold achieved
VAS pain during walking at <1 month	Laser vs. Placebo	MD, −2.50; 95% CI, −3.88 to −1.11	Moderate	Yes
VAS pain during walking at 1–3 months	Laser vs. Placebo	MD, −2.49; 95% CI, −4.51 to −0.48	Low	Yes
VAS pain during walking at >3 months	neuromuscular exercise vs. Placebo	MD, −1.77; 95% CI, −3.14 to −0.39	Low	No
VAS pain at rest	TENS vs. Placebo	MD, −2.87; 95% CI, −4.87 to −0.87	Unable to assess	Yes
NRS	TENS vs. Placebo	MD, −2.73; 95% CI, −5.20 to −0.26	Unable to assess	Yes
WOMAC total scores at <1 month	Shockwave vs. Placebo	MD, −9.56; 95% CI, −16.71 to −2.41	Low	Yes
WOMAC total scores at 1–3 months	Aquatic sports vs. Placebo	MD, −16.53; 95% CI, −30.74 to −2.31	Low	Yes
WOMAC total scores at >3 months	Shockwave vs. Placebo	MD, −30.02; 95% CI, −40.39 to −19.65	Moderate	Yes

### Important but not crucial outcomes

For post-treatment WOMAC pain at <1 month, we analyzed 13 trials involving 976 patients; 1–3 months, 32 trials involving 1731 patients, and >3 months, 17 trials involving 1,417 patients. Shockwave (MD, −3.14; 95% CI, −5.71 to −0.57), aquatic sports (MD, −1.81; 95% CI, −3.24 to −0.39), TENS (MD, −4.27; 95% CI, −6.21 to −2.34) and strength training (MD, −2.79; 95% CI, −4.58 to −0.99) all showing significant effects compared to placebo with low confidence (Appendix 8: Supplementary Figures S8.6–S8.8).

For WOMAC stiffness scores at <1 month, 1–3 months, and >3 months, we analyzed 12, 25, and 11 trials involving 756, 1,289, and 736 patients, respectively. Only aerobic exercise (MD, −1.81; 95% CI, −2.85 to −0.77; low confidence) and TENS (MD, −1.64; 95% CI, −2.75 to −0.53; low confidence) effectively reduce stiffness compared to placebo (Appendix 8: Supplementary Figures S8.9–S8.11).

For WOMAC function scores at <1 month, 1–3 months, and >3 months, we analyzed 11, 32, and 15 trials involving 689, 1753, and 1,147 patients, respectively. Compared to placebo, shockwave (MD, −11.00; 95% CI, −20.77 to −1.23; low confidence), and TENS (MD, −12.23; 95% CI, −18.74 to −5.72; low confidence) were most effective in reducing functional scores at <1 month and 1–3 months, respectively. For WOMAC function at >3 months, no significant differences were found in the efficacy among the interventions (Appendix 8: Supplementary Figures S8.12–S8.14).

### Sensitivity analyses and subgroup analyses

The high heterogeneity observed among the 21 studies may primarily be attributed to differences in participant characteristics (such as age and baseline pain levels) and variations in the intervention protocols (such as treatment frequency and intensity). A sensitivity NMA restricted to studies at low risk of bias was conducted for the VAS pain during walking at 1–3 months, with consistent results. The sensitivity analysis proved to be consistent with the main results (Appendix 10), thereby confirming the robustness of our research findings. Subgroup analyses showed that for WOMAC stiffness at 1–3 months and WOMAC function at 1–3 months, TENS has only been observed for effective pain relief in patients under 63 years of age with KOA. Besides, for WOMAC pain at 1–3 months, WOMAC function at 1–3 months and WOMAC total score at 1–3 months, shockwave provided pain relief only in patients with BMI < 29 kg/m2 (Appendix 11).

### Adverse events

Across 16 trials involving 1,582 patients, 130 adverse events were reported, with a person-based incidence rate of 8.1%. The most commonly reported adverse events were increased knee pain (30.0%) and mild acupuncture-related events (52.3%). None of the interventions showed a higher risk of overall adverse events compared to placebo. However, strength training (OR, 19.79; 95% CI, 1.14 to 344.82) and neuromuscular exercise (OR, 39.26; 95% CI, 1.92 to 801.44) demonstrated a significantly higher odds ratio of adverse events compared to general nursing (Appendix 12).

## Discussion

### Principal finding

This NMA comprehensively evaluated and compared the efficacy and safety of 13 interventions for knee osteoarthritis, involving 74 RCTs and 3,707 participants. For pain control, 11 physical therapies demonstrated significant beneficial results compared to placebo, and laser, shockwave, TENS and aquatic sports proved among the best with moderate to low confidence of evidence. In terms of improving WOMAC total, shockwave and aquatic sports proved among the most effective compared to placebo with moderate confidence of evidence. There is a higher risk of overall adverse events with strength training and neuromuscular exercise compared with general nursing. It should be noted that the relative effect sizes of physical therapies were smaller when compared with general nursing than with placebo.

### Comparisons with other studies

To our knowledge, there was no previous NMA comparing the wide range of physical therapies used in patients with KOA, but exist several systematic review or small meta-analyses comparing specific interventions. The findings of our current and previous analyses indicate that aquatic sports is the most effective exercise therapy for pain relief (29). However, it typically takes at least 6 weeks for patients to experience the full range of clinical benefits, which may pose a challenge for patient adherence (30). Our pooling results also reflected this point, as exercise therapy lasting less than 1 month did not effectively alleviate pain. Some previous RCTs and meta-analyses have focused on comparing TENS, shockwave and laser with placebo, rather than directly comparing these three interventions (31–33). These studies have shown that these interventions are superior to placebo in terms of analgesic effect, and our NMA reconfirmed this result that 76.91% of patients were able to achieve the predetermined MCID with moderate to low confidence of evidence.

Individualized exercise has been shown to be an effective intervention for patients with KOA, and its superior safety profile further enhances its potential as a more promising and competitive option for wider adoption. As guidelines and clinical practice have evolved, the primary focus has shifted to determining which exercise modality is most appropriate. According to the literature, the exercise therapies most commonly prescribed and emphasized by physical therapists are resistance training and aquatic sports (34), compared to traditional strength training, aquatic sports demonstrated to be more beneficial than strength training, as it can reduce joint pressure by 36 to 55% and assist with movement under the influence of buoyancy (35). One potential concern may be ensuring patient compliance with the recommended exercise regimen, as it requires individuals to engage in at least three sessions per weeks, to achieve the optimal outcomes (36). Consequently, exercise intervention plans should prioritize the enhancement of exercise adherence. Besides, the optimal timing and intensity of exercise remain unclear (37), Our research quantity is relatively limited, and we have not conducted subgroup analysis on different doses, making it impossible to draw clear conclusions based on the included experiments.

The existing literature does not provide clear evidence regarding the efficacy of TENS, shockwave, and laser for pain relief for KOA and there is no consensus among the guidelines regarding the use of these modalities. In this NMA, we included RCTs evaluating TENS, shockwave, and laser, however, most were non-head-to-head, and with low quality and small scale. The pooled estimates from these studies suggest that these therapies can effectively improve pain and stiffness in KOA, but the low confidence of evidence weakens the certainty of these results. Given the emerging nature of this evidence, clinicians should remain alert to new findings. Recent high-quality research has identified dose–response relationships for these interventions, and suggest patients with KOA, who are hindered from engaging in exercise therapy due to severe pain, should consider a moderate dose of shockwave therapy (0.08–0.25 mJ/mm^2^), high-energy laser therapy (19 to 150 J/cm2), or high-frequency TENS therapy (100 Hz) (38–40). In the absence of definitive evidence, physiotherapists should exercise their clinical expertise to determine the most suitable intervention, considering the patient’s unique symptom severity, personal expectations, and tolerance for treatment.

### Study limitations

This study represents the most comprehensive and up-to-date systematic review and meta-analysis of physical therapy, encompassing almost all available treatment methods, involving sound, light, force, and electricity. We rigorously applied the CINeMA quality assessment method, thereby enhancing credibility for the research findings. However, it is important to acknowledge the limitations of our study. Firstly, the risk of bias in the studies we included was assessed as either unclear or high, which ultimately resulted in a low to-moderate degree of confidence in the CINeMA estimates. This introduces an inherent source of bias that needs to be taken into consideration when interpreting the findings. Secondly, the effectiveness and safety of physical therapy may vary depending on the specific doses and levels applied, regrettably, the current analysis was limited by the insufficient number of trials available to thoroughly investigate this potential effect modification with the necessary statistical precision. Thirdly, the included trials have varying population characteristics and follow-up periods, which could potentially introduce imprecise effect estimates, although the consistency of the results across these trials mitigated this concern. Finally, for certain outcome variables, there were fewer than 10 RCTs available, which precluded the possibility of examining the risk of bias and evaluating the quality of evidence for these specific outcomes.

## Conclusion

In conclusion, physical interventions other than balance exercise and proprioceptive exercise training proved more effective than placebo in alleviating pain symptom in knee osteoarthritis.TENS, laser, and neuromuscular exercise therapies demonstrated the greatest efficacy in reducing scores on the NRS and VAS scales; and shockwave and aquatic sports demonstrated among the most effective ways to improve WOMAC total score. Nonetheless, the majority of comparisons examining the effectiveness of these therapies are supported by only moderate-to-low levels of evidence, underscoring the need for additional, large-sample, multicenter, higher-quality head-to-head studies to more conclusively establish their clinical efficacy.

## Data Availability

The original contributions presented in the study are included in the article/Supplementary material, further inquiries can be directed to the corresponding authors.

## Footnotes

1
hkbu.edu.hk

2
http://cinema.ispm.unibe.ch/#general

## References

[1] Wiggers TGH Winters M Van den Boom NAC Haisma HJ Moen MH. Autologous stem cell therapy in knee osteoarthritis: a systematic review of randomised controlled trials. Br J Sports Med. (2021) 55:1161–9. doi: 10.1136/bjsports-2020-103671, PMID: 34039582

[2] Cai G Aitken D Laslett LL Pelletier JP Martel-Pelletier J Hill C . Effect of intravenous Zoledronic acid on tibiofemoral cartilage volume among patients with knee osteoarthritis with bone marrow lesions. JAMA. (2020) 323:1456–66. doi: 10.1001/jama.2020.2938, PMID: 32315057 PMC7175085

[3] Bichsel D Liechti FD Schlapbach JM Wertli MM. Cross-sectional analysis of recommendations for the treatment of hip and knee osteoarthritis in clinical guidelines. Arch Phys Med Rehabil. (2022) 103:559–569.e5. doi: 10.1016/j.apmr.2021.07.801, PMID: 34411512

[4] Conaghan PG Cook AD Hamilton JA Tak PP. Therapeutic options for targeting inflammatory osteoarthritis pain. Nat Rev Rheumatol. (2019) 15:355–63. doi: 10.1038/s41584-019-0221-y, PMID: 31068673

[5] Berry KM Neogi T Baker JF Collins JM Waggoner JR Hsiao CW . Obesity progression between young adulthood and midlife and incident arthritis: A retrospective cohort study of US adults. Arthritis Care Res. (2021) 73:318–27. doi: 10.1002/acr.24252, PMID: 32374930 PMC7644635

[6] Krishnamurthy A Lang AE Pangarkar S Edison J Cody J Sall J. Synopsis of the 2020 US Department of veterans affairs/US Department of defense clinical practice guideline: the non-surgical Management of hip and Knee Osteoarthritis. Mayo Clin Proc. (2021) 96:2435–47. doi: 10.1016/j.mayocp.2021.03.017, PMID: 34481599

[7] Beaudart C Lengelé L Leclercq V Geerinck A Sanchez-Rodriguez D Bruyère O . Symptomatic efficacy of pharmacological treatments for knee osteoarthritis: A systematic review and a network Meta-analysis with a 6-month time horizon. Drugs. (2020) 80:1947–59. doi: 10.1007/s40265-020-01423-8, PMID: 33074440 PMC7716887

[8] Kolasinski SL Neogi T Hochberg MC Oatis C Guyatt G Block J . 2019 American College of Rheumatology/Arthritis Foundation guideline for the Management of Osteoarthritis of the hand, hip, and knee. Arthritis Rheumatol. (2020) 72:220–33. doi: 10.1002/art.41142, PMID: 31908163 PMC10518852

[9] Brophy RH Fillingham YA. AAOS clinical practice guideline summary: Management of Osteoarthritis of the knee (nonarthroplasty), third edition. J Am Acad Orthop Surg. (2022) 30:e721–9. doi: 10.5435/jaaos-d-21-01233, PMID: 35383651

[10] Rutjes AWS Nüesch E Sterchi R Kalichman L Hendriks E Osiri M . Transcutaneous electrostimulation for osteoarthritis of the knee. Cochrane Database Syst Rev. (2009) 2009:CD002823. doi: 10.1002/14651858.CD002823.pub2, PMID: 19821296 PMC7120411

[11] Bartels EM Juhl CB Christensen R Hagen KB Danneskiold-Samsøe B Dagfinrud H . Aquatic exercise for the treatment of knee and hip osteoarthritis. Cochrane Database Syst Rev. (2016) 3:CD005523. doi: 10.1002/14651858.CD005523.pub3, PMID: 17943863

[12] Yeğin T Altan L Kasapoğlu Aksoy M. The effect of therapeutic ultrasound on pain and physical function in patients with knee osteoarthritis. Ultrasound Med Biol. (2017) 43:187–94. doi: 10.1016/j.ultrasmedbio.2016.08.035, PMID: 27727020

[13] Zhang Y Xie S Wang X Song K Wang L Zhang R . Effects of internet of things-based power cycling and neuromuscular training on pain and walking ability in elderly patients with KOA: protocol for a randomized controlled trial. Trials. (2022) 23:1009. doi: 10.1186/s13063-022-06946-x, PMID: 36514174 PMC9745721

[14] Wang Q Lv H Sun Z-T Tu JF Feng YW Wang TQ . Effect of Electroacupuncture versus sham Electroacupuncture in patients with knee osteoarthritis: A pilot randomized controlled trial. Evid Based Complement Alternat Med. (2020) 2020:1–9. doi: 10.1155/2020/1686952, PMID: 32802114 PMC7414333

[15] Page MJ McKenzie JE Bossuyt PM Boutron I Hoffmann TC Mulrow CD . The PRISMA 2020 statement: an updated guideline for reporting systematic reviews. BMJ. (2021) 372:n71. doi: 10.1136/bmj.n71, PMID: 33782057 PMC8005924

[16] Yuan F Dong H Gong J Wang D Hu M Huang W . A systematic review and Meta-analysis of randomized controlled trials on the effects of turmeric and Curcuminoids on blood lipids in adults with metabolic diseases. Adv Nutr. (2019) 10:791–802. doi: 10.1093/advances/nmz021, PMID: 31212316 PMC6743846

[17] Laigaard J Pedersen C Rønsbo TN Mathiesen O Karlsen APH. Minimal clinically important differences in randomised clinical trials on pain management after total hip and knee arthroplasty: a systematic review. Br J Anaesth. (2021) 126:1029–37. doi: 10.1016/j.bja.2021.01.021, PMID: 33678402

[18] Hoffman KE Penson DF Zhao Z Huang LC Conwill R Laviana AA . Patient-reported outcomes through 5 years for active surveillance, surgery, brachytherapy, or external beam radiation with or without androgen deprivation therapy for localized prostate Cancer. JAMA. (2020) 323:149–63. doi: 10.1001/jama.2019.20675, PMID: 31935027 PMC6990712

[19] Hinman RS McCrory P Pirotta M Relf I Forbes A Crossley KM . Acupuncture for chronic knee pain. JAMA. (2014) 312:1313. doi: 10.1001/jama.2014.12660, PMID: 25268438

[20] Bennell KL Kyriakides M Metcalf B Egerton T Wrigley TV Hodges PW . Neuromuscular versus quadriceps strengthening exercise in patients with medial knee osteoarthritis and varus malalignment: a randomized controlled trial. Arthritis Rheumatol. (2014) 66:950–9. doi: 10.1002/art.38317, PMID: 24757146

[21] Messier SP Mihalko SL Beavers DP Nicklas BJ DeVita P Carr JJ . Effect of high-intensity strength training on knee pain and knee joint compressive forces among adults with knee osteoarthritis. JAMA. (2021) 325:646–57. doi: 10.1001/jama.2021.0411, PMID: 33591346 PMC7887656

[22] Feng J Li Z Tian L Mu P Hu Y Xiong F . Efficacy and safety of curcuminoids alone in alleviating pain and dysfunction for knee osteoarthritis: a systematic review and meta-analysis of randomized controlled trials. BMC Complement Med Ther. (2022) 22:276. doi: 10.1186/s12906-022-03740-9, PMID: 36261810 PMC9580113

[23] Lv Z-t Shen L-l Zhu B Zhang ZQ Ma CY Huang GF . Effects of intensity of electroacupuncture on chronic pain in patients with knee osteoarthritis: a randomized controlled trial. Arthritis Res Ther. (2019) 21:120. doi: 10.1186/s13075-019-1899-6, PMID: 31088511 PMC6518678

[24] Higgins JPT Altman DG Gotzsche PC Juni P Moher D Oxman AD . The Cochrane collaboration's tool for assessing risk of bias in randomised trials. BMJ. (2011) 343:d5928–8. doi: 10.1136/bmj.d5928, PMID: 22008217 PMC3196245

[25] Papakonstantinou T Nikolakopoulou A Higgins JPT Egger M Salanti G. CINeMA: software for semiautomated assessment of the confidence in the results of network meta-analysis. Campbell Syst Rev. (2020) 16:16. doi: 10.1002/cl2.1080, PMID: 37131978 PMC8356302

[26] Nikolakopoulou A Higgins JPT Papakonstantinou T Chaimani A del Giovane C Egger M . CINeMA: an approach for assessing confidence in the results of a network meta-analysis. PLoS Med. (2020) 17:e1003082. doi: 10.1371/journal.pmed.1003082, PMID: 32243458 PMC7122720

[27] Chawla N Anothaisintawee T Charoenrungrueangchai K Thaipisuttikul P McKay GJ Attia J . Drug treatment for panic disorder with or without agoraphobia: systematic review and network meta-analysis of randomised controlled trials. BMJ. (2022) 376:e066084. doi: 10.1136/bmj-2021-066084, PMID: 35045991 PMC8767458

[28] Basu S Ranzani OT Kalra A Di Girolamo C Curto A Valerio F . Urban-rural differences in hypertension prevalence in low-income and middle-income countries, 1990–2020: a systematic review and meta-analysis. PLoS Med. (2022) 19:e1004079. doi: 10.1371/journal.pmed.1004079 36007101 PMC9410549

[29] Mo L Jiang B Mei T Zhou D. Exercise therapy for knee osteoarthritis: a systematic review and network meta-analysis. Orthop J Sports Med. (2023) 11:23259671231172773. doi: 10.1177/23259671231172773, PMID: 37346776 PMC10280533

[30] Dantas LO Salvini TF McAlindon TE. Knee osteoarthritis: key treatments and implications for physical therapy. Braz J Phys Ther. (2021) 25:135–46. doi: 10.1016/j.bjpt.2020.08.004, PMID: 33262080 PMC7990728

[31] Tang P Wen T Lu W Jin H Pan L Li H . The efficacy of extracorporeal shock wave therapy for knee osteoarthritis: an umbrella review. Int J Surg. (2024) 110:2389–95. doi: 10.1097/js9.0000000000001116, PMID: 38668665 PMC11020044

[32] Wu Y Zhu F Chen W Zhang M. Effects of transcutaneous electrical nerve stimulation (TENS) in people with knee osteoarthritis: a systematic review and meta-analysis. Clin Rehabil. (2021) 36:472–85. doi: 10.1177/02692155211065636, PMID: 34971318

[33] Stausholm MB Naterstad IF Joensen J Lopes-Martins RÁB Sæbø H Lund H . Efficacy of low-level laser therapy on pain and disability in knee osteoarthritis: systematic review and meta-analysis of randomised placebo-controlled trials. BMJ Open. (2019) 9:e031142. doi: 10.1136/bmjopen-2019-031142, PMID: 31662383 PMC6830679

[34] Gonçalves RS Pimenta N Martins PN Ferreira RM. Physical therapists’ choices, views and agreements regarding non-pharmacological and non-surgical interventions for knee osteoarthritis patients: a mixed-methods study. Mediterr J Rheumatol. (2023) 34:188–219. doi: 10.31138/mjr.34.2.188, PMID: 37654628 PMC10466349

[35] Xu Z Wang Y Zhang Y Lu Y Wen Y. Efficacy and safety of aquatic exercise in knee osteoarthritis: a systematic review and meta-analysis of randomized controlled trials. Clin Rehabil. (2022) 37:330–47. doi: 10.1177/02692155221134240, PMID: 36320162

[36] Juhl C Christensen R Roos EM Zhang W Lund H. Impact of exercise type and dose on pain and disability in knee osteoarthritis: A systematic review and Meta-regression analysis of randomized controlled trials. Arthritis Rheumatol. (2014) 66:622–36. doi: 10.1002/art.38290, PMID: 24574223

[37] van Doormaal MCM Meerhoff GA Vliet Vlieland TPM Peter WF. A clinical practice guideline for physical therapy in patients with hip or knee osteoarthritis. Musculoskeletal Care. (2020) 18:575–95. doi: 10.1002/msc.1492, PMID: 32643252

[38] Charles A Shukrimi B Zamzuri B Ardilla HBAR. Portable transcutaneous electrical nerve stimulation therapy at different frequencies in the treatment of knee osteoarthritis: A quasi-experimental study. J Orthop Case Rep. (2020) 10:108–13. doi: 10.13107/jocr.2020.v10.i03.1772, PMID: 33954149 PMC8051567

[39] Ahmad MA Moganan M A Hamid MS Sulaiman N Moorthy U Hasnan N . Comparison between low-level and high-intensity laser therapy as an adjunctive treatment for knee osteoarthritis: a randomized, double-blind clinical trial. Life. (2023) 13:1519. doi: 10.3390/life13071519, PMID: 37511894 PMC10381799

[40] Liao C-D Huang Y-Y Chen H-C Liou TH Lin CL Huang SW. Relative effect of extracorporeal shockwave therapy alone or in combination with noninjective treatments on pain and physical function in knee osteoarthritis: A network Meta-analysis of randomized controlled trials. Biomedicine. (2022) 10:306. doi: 10.3390/biomedicines10020306, PMID: 35203516 PMC8869515

