# Supplementary material for: Effectiveness of physical therapies for patients with knee osteoarthritis: a systematic review and network meta-analysis of randomized controlled trials
Source: Front Med (Lausanne). 2025 Dec 4;12:1714912. doi: 10.3389/fmed.2025.1714912 (PMC12711732; doi:10.3389/fmed.2025.1714912)
Supplement: Supplementary file 2 [file Data_Sheet_2.doc]

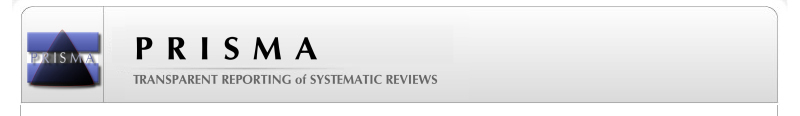
**PRISMA 2020 Flow Diagram**

**Screening**

**Included**

**Eligibility**

**Identification**

Records identified through database searching
(n = 3785)

Additional records identified through other sources
(n =85)

Records after duplicates removed
(n = 2443)

Records screened
(n =2443)

Records excluded
(n = 2208)

Full-text articles assessed for eligibility
(n = 235)

Full-text articles excluded, with reasons
 (Inappropriate study type(n=10)

Ineligible intervention(n=39)

No relevant outcome (n=37)

Inappropriate data type (n=66)

Incorrect population (n=9))

Studies included in qualitative synthesis
(n = 74)

Studies included in quantitative synthesis (meta-analysis)
(n = 74)
