# Supplementary material for: Effectiveness of physical therapies for patients with knee osteoarthritis: a systematic review and network meta-analysis of randomized controlled trials
Source: Front Med (Lausanne). 2025 Dec 4;12:1714912. doi: 10.3389/fmed.2025.1714912 (PMC12711732; doi:10.3389/fmed.2025.1714912)
Supplement: Supplementary file 3 [file Data_Sheet_3.docx]

**Supplementary Appendix**

**Effectiveness of physical therapies for patients with** **knee osteoarthritis: a systematic review and network meta-analysis of randomized controlled trials**

Content

[**Appendix 1: Search strategy** 1](#_Toc213090002)

[**Appendix 2: Characteristics of included studies** 5](#_Toc213090003)

[**Appendix 3: Risk of bias of randomized clinical trials** 18](#_Toc213090004)

[**Appendix 4: CINeMA Assessment** 23](#_Toc213090005)

[**Appendix 5: Evaluation of inconsistency and heterogeneity** 38](#_Toc213090006)

[**Appendix 6: Network maps** 54](#_Toc213090007)

[**Appendix 7: Funnel plots** 71](#_Toc213090008)

[**Appendix 8: Comprehensive comparisons of different types of physical therapies** 86](#_Toc213090009)

[**Appendix 9: Cumulative probability plots** 103](#_Toc213090010)

[**Appendix 10: The results of sensitivity Analyses** 121](#_Toc213090011)

[**Appendix 11: The results of subgroup analyse** 153](#_Toc213090012)

[**Appendix 12: Adverse events** 175](#_Toc213090013)

[**Appendix 13: contribution matrix** 180](#_Toc213090014)

**Appendix 1: Search strategy**

**Table S1.** Search strategy of PubMed

| # | **Searches** |
| --- | --- |
| 1 | osteoarthritis, knee [MeSH Major Topic] |
| 2 | osteoarthritis of knee [Title/Abstract] OR knee osteoarthritis [Title/Abstract] OR knee osteoarthritis [Title/Abstract] OR osteoarthritis of the knee [Title/Abstract] |
| 3 | #1 AND #2 |
| 4 | (randomized controlled trial [Filter]) |
| 5 | Electroacupuncture [Title/Abstract] OR electric needle [Title/Abstract] OR electro-needling [Title/Abstract] |
| 6 | Laser [Title/Abstract] OR laser beam [Title/Abstract] |
| 7 | transcutaneous electrical nerve stimulation [Title/Abstract] OR transcutaneous nerve stimulation [Title/Abstract] |
| 8 | ultrashort wave [Title/Abstract] |
| 9 | interferential therapy [Title/Abstract] OR Interference therapy [Title/Abstract] |
| 10 | Ultrasound [Title/Abstract] OR ultrasonic wave [Title/Abstract] OR ultrasonic [Title/Abstract] |
| 11 | shock wave [Title/Abstract] OR blast wave [Title/Abstract] |
| 12 | aerobic exercise [Title/Abstract] OR aerobic training [Title/Abstract] OR aerobics [Title/Abstract] |
| 13 | power training [Title/Abstract] OR strength training [Title/Abstract] |
| 14 | neuromuscular exercise [Title/Abstract] |
| 15 | aquatic sports [Title/Abstract] OR water sport [Title/Abstract] |
| 16 | balance training [Title/Abstract] |
| 17 | proprioception exercise [Title/Abstract] |
| 18 | #5 OR #6 OR #7 OR #8 OR #9 OR #10 OR #11 OR #12 OR #13 OR #14 OR #15 OR #16 OR #17 |
| 19 | Pain [Title/Abstract] OR discomfort*[Title/Abstract] OR stiff*[Title/Abstract] |
| 20 | #3 AND #4 AND #18 AND #19 |

**Table S2.** Search strategy of Web of Science

| # | | **Searches** |
| --- | --- | --- |
| 1 | (((TS= (osteoarthritis of knee)) OR TS= (knee osteoarthritides)) OR TS= (knee osteoarthritis)) OR TS= (osteoarthritis of the knee) | |
| 2 | ((TS=(pain)) OR TS=(discomfort*)) OR TS= (stiff*) | |
| 3 | ((((((((((((((((((((((((TS=(electroacupuncture)) OR TS=(electric needle)) OR TS=(electro-needling)) OR TS=(laser)) OR TS=(laser beam)) OR TS=( transcutaneous electrical nerve stimulation)) OR TS=(transcutaneous nerve stimulation)) OR TS=(ultrashort wave)) OR TS=( interferential therapy)) OR TS=(Interference therapy)) OR TS=( ultrasound)) OR TS=(ultrasonic wave)) OR TS=(ultrasonic)) OR TS=(shock wave)) OR TS=( blast wave)) OR TS=(aerobic exercise)) OR TS=(aerobic training)) OR TS=(aerobics)) OR TS=(power training)) OR TS=(strength training)) OR TS=(neuromuscular exercise)) OR TS=(aquatic sports)) OR TS=( water sport)) OR TS=(balance training)) OR TS=(proprioception exercise) | |
| 4 | ((((TS= (randomized controlled trial)) OR TS= (controlled clinical trial)) OR TS=(randomly)) OR TS= (randomized)) OR TS= (randomized trial) | |
| 5 | #1 AND #2 AND #3 AND #4 | |

**Table S3.** Embase

| # | | **Searches** |
| --- | --- | --- |
| 1 | ‘osteoarthritis of knee’ OR ‘knee osteoarthritides’ OR ‘knee osteoarthritis’ OR ‘osteoarthritis of the knee’ | |
| 2 | ‘pain’ OR ‘discomfort’ OR ‘stiff*’ | |
| 3 | ‘electroacupuncture’ OR ‘electric needle’ OR ‘electro-needling’ OR ‘laser’ OR ‘laser beam’ OR ‘transcutaneous electrical nerve stimulation’ OR ‘transcutaneous nerve stimulation’ OR ‘ultrashort wave’ OR ‘interferential therapy’ OR ‘Interference therapy’ OR ‘ultrasound’ OR ‘ultrasonic wave’ OR ‘ultrasonic’ OR ‘shock wave’ OR ‘blast wave’ OR ‘aerobic exercise’ OR ‘aerobic training’ OR ‘aerobics’ OR ‘power training’ OR ‘strength training’ OR ‘neuromuscular exercise’ OR ‘aquatic sports’ OR ‘water sport’ OR ‘balance training’ OR ‘proprioception exercise’ | |
| 4 | ‘randomized controlled trial’ OR ‘controlled clinical trial’ OR ‘randomly’ OR ‘randomized ‘ OR ‘randomized trial’ | |
| 5 | #1 AND #2 AND #3 AND #4 | |

**Table S4.** Cochrane library

| # | | **Searches** |
| --- | --- | --- |
| 1 | osteoarthritis,knee | |
| 2 | "osteoarthritis of knee" OR "knee osteoarthritides" OR "knee osteoarthritis" OR "osteoarthritis of the knee" | |
| 3 | ‘electroacupuncture’ OR ‘electric needle’ OR ‘electro-needling’ OR ‘laser’ OR “pain” OR “discomfort” OR “stiff*” | |
| 4 | “electroacupuncture” OR “electric needle” OR “electro-needling” OR “laser” OR “laser beam” OR “transcutaneous electrical nerve stimulation” OR “transcutaneous nerve stimulation” OR “ultrashort wave” OR “interferential therapy” OR “Interference therapy” OR “ultrasound” OR “ultrasonic wave” OR “ultrasonic” OR “shock wave” OR “blast wave” OR “aerobic exercise” OR “aerobic training” OR “aerobics” OR “power training” OR “strength training” OR “neuromuscular exercise” OR “aquatic sports” OR “water sport” OR “balance training” OR “proprioception exercise” | |
| 5 | #1 AND #2 AND #3 AND #4 | |

**Appendix 2: Characteristics of included studies**

**Table S2.1:** Baseline of characteristics of included studies.

| First  author's name | Journal | Study country | diagnostic criteria | Average age (years) | BMI | Sample size (F/M) | Treatment period | Outcome | Treatment | Number of lost visits |
| --- | --- | --- | --- | --- | --- | --- | --- | --- | --- | --- |
| Rogers2012^1^ | Journal of Sports Science and Medicine | USA | Diagnostic criteria of the American Rheumatology Society | 70.0±10.07 | 28.2 | 8(6:2) | 8 weeks | (iii) | Resistance training:Perform 15 repetitions per leg in a single set | NA |
|  |  |  |  | 71.2±10.9 | 30.8 | 8(6:2) |  |  | sham treatment | NA |
| Messier  2021^2^ | JAMA | USA | Kellgren Lawrence standard diagnosis | 67±9 | 31±6 | 108(52:75) | 18 months | (iii) | High intensity strength training:Three times a weekth training | 19 |
|  |  |  |  | 64±8 | 31±6 | 104(51:75) |  |  | Low intensity strength training :Three times a weekth training | 22 |
|  |  |  |  | 64±7 | 32±5 | 99(48:76) |  |  | attention control | 25 |
| Law2004^3^ | J Rehabil Med | China | Kellgren Lawrence standard diagnosis | 82.7±6.1 | 25.0±2.8 | 13(13:0) | 2 weeks | (ii) | TENS:2Hz | Two people have exited in total |
|  |  |  |  | 84.3±6.9 | 24.8±3.5 | 12(12:0) |  |  | TENS:100Hz |  |
|  |  |  |  | 80.00±5.8 | 26.4±6.1 | 13(12:1) |  |  | TENS:2/100GHz |  |
|  |  |  |  | 83.2±5.4 | 29.2±6.7 | 10(10:0) |  |  | Placebo |  |
| Bennell  2014^4^ | ARTHRITIS & RHEUMATOLOGY | Australia | Radiological manifestations | 62.7±7.3 | 29.6±3.9 | 38(24:26) | 12 weeks | (ii)(iii) | Neuromuscular movement:8 sets, repeat 15 times | 12 |
|  |  |  |  | 62.2±7.4 | 29.7±4.3 | 44(26:24) |  |  | strength training:2-3 sets, repeat 10 times | 6 |
| Elerian2016^5^ | Int J Physiother | Egypt | Kellgren Lawrence standard diagnosis | 51±3.5 | NA | 20 | 3 weeks | (ii)(iii) | shock wave:5Hz | 0 |
|  |  |  |  | 51±3.5 | NA | 20 |  |  | Fake treatment | 0 |
| Chang2012^6^ | Disability and Rehabilitation | Taiwan | Kellgren Lawrence standard diagnosis | 65.0±8.4 | 24.9±3.3 | 24(24:0) | 8 weeks | (iii) | Resistance training:Repeat 10 times | 6 |
|  |  |  |  | 70.0±8.4 | 25.7±3.6 | 17(17:0) |  |  | general nursing | 13 |
| Tsauo2008^7^ | Clinical Rehabilitation | Taiwan | Kellgren Lawrence standard diagnosis | 61.7±6.6. | 26.5±3.5 | 15(14:1) | 8 weeks | (iii) | Proprioceptive motor training | 15 |
|  |  |  |  | 60.1±6.7 | 28.5±5.8 | 14(10:4) |  |  | general nursing | 16 |
| RPT2008^8^ | J Rehabil Med | Denmark | Diagnostic criteria of the American Rheumatology Society | 65±12.6 | NA | 27(22:5) | 8 weeks | (ii) | Water sports:Each session lasts 50 minutes | 2 |
|  |  |  |  | 70±9.9 | NA | 24(18:9) |  |  | Not exercising | 3 |
| Karakaş2020^9^ | Clinical Rehabilitation | Turkey | Diagnostic criteria of the American Rheumatology Society | 59.1±7.45 | 28.7±4.86 | 39(31:8) | 8 weeks | (ii)(iii) | ultrasonic | 9 |
|  |  |  |  | 60.75±7.46 | 29.22±10.13 | 36(32:4） |  |  | False ultrasound | 12 |
| Sadeghi  2023^10^ | Caspian Journal of Internal Medicine | Iran | Kellgren Lawrence standard diagnosis | 52.8±9.6 | 29.22±3.77 | 24(20:4) | 8 weeks | (ii) | Quadriceps strength training | 0 |
|  |  |  |  | 49.6±7.4 | 28.54±4.18 | 24（18:6） |  |  | Hamstring strength training | 0 |
|  |  |  |  | 52.8±9.7 | 28.61±4.34 | 24(21:3) |  |  | Strength training for quadriceps and hamstring muscles | 0 |
|  |  |  |  | 55.7±9.2 | 31.32±5.03 | 24(17:7) |  |  | no movement | 0 |
| YURTKURAN2007^11^ | Photomedicine and Laser Surgery | Turkey | Diagnostic criteria of the American Rheumatology Society | 51.83±6.83 | 31.76±8.8.1 | 27(27:1) | 2 weeks | (ii)(iii) | Laser:The laser output power is 4 mW | 1 |
|  |  |  |  | 53.478±7.13 | 32.72±3.71 | 27（26:1） |  |  | Placebo laser | 1 |
| TASCIOGLU  2010^12^ | The Journal of International Medical Research | Turkey | Diagnostic criteria of the American Rheumatology Society | 59.7±2.63 | 29.96±4.02 | 27(17:10) | 2 weeks | (ii)(iii) | Ultrasound:1 MHz frequency and 2 W/cm2 power | 3 |
|  |  |  |  | 61.64±3.74 | 30.82±3.84 | 28（21:7） |  |  | Pulse ultrasound | 2 |
|  |  |  |  | 60.04±2.83 | 28.70±3.98 | 27(18:9) |  |  | sham ultrasound | 3 |
| McIlroy  2017^13^ | Musculoskeletal care | Bratain | NA | 64.3±8.7 | 32.4±6.2 | 7 | 6 weeks | (ii)(iii) | Water sports | 0 |
|  |  |  |  | 62.3±6.6 | 34.6±10.3 | 6 |  |  | general nursing | 1 |
| DeVita2018^14^ | Clinical Biomechanics | USA | Diagnostic criteria of the American Rheumatology Society | 58.1±6.5 | 26.4±4.0 | 15(10:5) | 12 weeks | (iii) | strength training:Perform 3 sets per group, 10 repetitions per set | 1 |
|  |  |  |  | 56.2±8.9 | 27.9±3.9 | 15（8:7） |  |  | No attention | 0 |
| ÖZGÖNENEL2009^15^ | Ultrasound in Medicine and Biology | Turkey | Diagnostic criteria of the American Rheumatology Society | 53.6±6.9 | NA | 34(28:6) | 2 weeks | (ii)(iii) | Ultrasonic:Frequency of 1 MHz, power of 1 watt/cm2 | 0 |
|  |  |  |  | 56.2±8.0 | NA | 33(26:7) |  |  | False ultrasound | 0 |
| Zhong2019^16^ | Archives of Physical Medicine and Rehabilitation | China | Diagnostic criteria of the American Rheumatology Society | 62.5±8.2 | 25.3±2.3 | 29(21:11) | 4 weeks | (ii)(iii) | shock wave:Once a week for four consecutive weeks | 3 |
|  |  |  |  | 63.2±7.7 | 25.4±2.9 | 29(19:12) |  |  | Fake treatment | 2 |
| Hinman  2014^17^ | JAMA | Australia | Clinical guidelines | 63.4±8.7 | 30.7±6.1 | 58(43:28) | 12 weeks | (i)(iii) | Laser:Energy output 0.2 J/point | 13 |
|  |  |  |  | 63.8±7.5 | 28.8±5.4 | 51(31:39) |  |  | Fake treatment | 19 |
| AN2008^18^ | THE JOURNAL OF ALTERNATIVE AND COMPLEMENTARY MEDICINE | China | Clinical symptoms and signs | 65.4±8.2 | 25.7±2.9 | 11 | 8 weeks | (iii) | Aerobic Exercise:Repeat 20 times | 3 |
|  |  |  |  | 64.6±6.7 | 25.4±2.9 | 10 |  |  | No intervention | 4 |
| Atamaz  2012^19^ | Archives of Physical Medicine And Rehabilitation | Turkey | Kellgren Lawrence standard diagnosis | 61.9±6.9 | 28.4±3.5 | 29(31:6) | 3 weeks | (ii)(iii) | TENS:80Hz | 8 |
|  |  |  |  | 62.0±7.9 | 29.8±3.4 | 27(27:4) |  |  | Interference current:100Hz | 4 |
|  |  |  |  | 60.7±6.5 | 29.0±4.1 | 33(27:10) |  |  | Sham TENS | 4 |
|  |  |  |  | 61.3±7.8 | 30.4±4.9 | 34(28:7) |  |  | Sham interference current | 1 |
| Hammam  2020^20^ | Journal of Taibah University Medical Sciences | Egypt | Diagnostic criteria of the American Rheumatology Society | 50.4±3.4 | 30.7±3.5 | 15(8:7) | 4 weeks | (ii) | Low energy shock wave:0.02 mJ/mm^2^ | 0 |
|  |  |  |  | 49.9±2.6 | 31±2.4 | 15(9:6) |  |  | High energy shock wave:0.178 mJ/mm^2^ | 0 |
|  |  |  |  | 49.7±3.1 | 31.1±3 | 15(9:6) |  |  | False shockwave | 0 |
| Artuç2023^21^ | Korean J Pain | Turkey | Kellgren Lawrence standard diagnosis | 58.5±9.8 | 30.33±6.62 | 19(16:3) | 2 weeks | (ii)(iii) | TENS:80 Hz | 1 |
|  |  |  |  | 56.05±9.45 | 29.34±4.57 | 18(15:3) |  |  | Placebo TENS | 2 |
|  |  |  |  | 61.95±11.78 | 31.43±3.51 | 18(14:4) |  |  | Interference current:100Hz | 2 |
|  |  |  |  | 54.00±8.79 | 31.20±3.74 | 18(15:3) |  |  | Sham interference current | 2 |
| Cheing  2002^22^ | Clinical Rehabilitation | China | Kellgren Lawrence standard diagnosis | 65.3±8.3 | 26.8±4.0 | 16(14:2) | 4 weeks | (ii) | TENS:80Hz | 0 |
|  |  |  |  | 64.1±6.1 | 28.8±3.7 | 14(15:1) |  |  | Placebo TENS | 2 |
| Weng2009^23^ | Kaohsiung J Med Sci | Taiwan | Kellgren Lawrence standard diagnosis | NA | NA | 28 | 8 weeks | (ii) | strength training:Three times a week | 5 |
|  |  |  |  | NA | NA | 26 |  |  | No intervention | 7 |
| Mostafa  2022^24^ | Photobiomodulation, Photomedicine, and Laser Surgery | Egypt | Diagnostic criteria of the American Rheumatology Society | 40.12±9.45 | 28.82±5.23 | 20(11:9) | 4 weeks | (ii)(iii) | shock wave:0.05 mJ/mm2 | 0 |
|  |  |  |  | 46.62±8.68 | 29.26±2.48 | 20(10:10) |  |  | Laser:30Hz | 0 |
| Draper2018^25^ | Journal of Orthopaedic Surgery and Research | USA | NA | 53.6±8.9 | 34.9±8.85 | 51(23:28) | 6 weeks | (i)(iii) | Ultrasonic:1.3W | 4 |
|  |  |  |  | 51±9.0 | 34.5±8.3 | 33(17:16) |  |  | placebo | 4 |
| Alghadir  2014^26^ | Lasers Med Sci | Saudi Arabia | Diagnostic criteria of the American Rheumatology Society | 55.2±8.14 | 32.34±5.77 | 20(10:10) | 4 weeks | (ii)(iii) | Laser:Wavelength of 850 nm | 0 |
|  |  |  |  | 57±7.77 | 33.09±4.98 | 20(8:12) |  |  | placebo | 0 |
| Reichenbach2022^27^ | Osteoarthritis and Cartilage | Switzerland | Diagnostic criteria of the American Rheumatology Society | 64.8±9.9 | 27.5±9.9 | 108(52:56) | 3 weeks | (iii) | TENS | 0 |
|  |  |  |  | 66.3±10.3 | 26.9±4.9 | 112(60:52) |  |  | placebo | 0 |
| Özgönenel2018^28^ | Journal of Medical Ultrasound | Turkey | Diagnostic criteria of the American Rheumatology Society | 54.0±17.08 | 28.8±5.6 | 15(9:6) | 2 weeks | (ii)(iii) | ultrasonic:1 MHz | 0 |
|  |  |  |  | 55.4±11.06 | 28.1±5.5 | 18(6:12) |  |  | Sham ultrasound | 0 |
| Cho2016^29^ | Annals of Rehabilitation Medicine | Korea | Kellgren Lawrence standard diagnosis | 75.5±7.7 | NA | 9(1:8) | 3 weeks | (ii) | shock wave:0.05 mJ/mm2 | 0 |
|  |  |  |  | 72.7±5.9 | NA | 9(2:7) |  |  | Sham shockwave | 0 |
| Lv2019^30^ | Arthritis Research And Therapy | China | Kellgren Lawrence standard diagnosis | 64.6±10.2 | 22.67±2.22 | 145(106:39) | 2 weeks | (ii)(iii) | Strong electroacupuncture:30 minutes | 14 |
|  |  |  |  | 63.7±9.3 | 22.46±1.87 | 64(57:7) |  |  | Weak electroacupuncture:30 minutes | 11 |
|  |  |  |  | 63.7±9.3 | 22.46±1.87 | 71(57:14) |  |  | Sham electroacupuncture | 5 |
| Mascarin  2012^31^ | BMC Musculoskelet Disord | Brazil | Diagnostic criteria of the American Rheumatology Society | 64.8±7.0 | NA | 12 | 12 weeks | (ii)(iii) | TENS:100Hz | 0 |
|  |  |  |  | 62.8±7.6 | NA | 12 |  |  | Ultrasonic:1 MHz | 0 |
| LIN2009^32^ | journal of orthopaedic & sports physical therapy | China | Clinical history, radiographic imaging, and physical evaluation | 63.7±8.2 | 27±9 | 36(25:11) | 8 weeks | (iii) | Proprioceptive motor training:20 minutes | 0 |
|  |  |  |  | 61.6±7.20 | 26±10 | 36(24:12) |  |  | strength training | 0 |
|  |  |  |  | 62.2±6.7 | 25±11 | 36(26:10) |  |  | No movement | 0 |
| Nazari2019^33^ | Lasers in Medical Science | Iran | Diagnostic criteria of the American Rheumatology Society | 61.5±3.9 | 27.7±1.40 | 30(17:13) | 4 weeks | (ii)(iii) | Laser:30Hz | 1 |
|  |  |  |  | 62.4±3.14 | 27.2±1.6 | 30(16:14) |  |  | Conventional physical therapy | 1 |
| Dadalto  2013^34^ | Fisioterapia em Movimento | Brazil | Diagnostic criteria of the American Rheumatology Society | NA | NA | 9 | 8 weeks | (iii) | Resistance training:Repeat 15 times | 1 |
|  |  |  |  | NA | NA | 6 |  |  | No intervention | 0 |
| Wang2020^35^ | Alternative therapies in health and medicine | China | NA | 63.5±10.6 | 24.8±2.3 | 33(12:24) | 10 weeks | (i)(iii) | shock wave:15 Hz/s | 3 |
|  |  |  |  | 64.3±11.1 | 25.2±2.4 | 31(15:21) |  |  | placebo | 5 |
| Dias2017^36^ | Brazilian Journal of Physical Therapy | Brazil | Diagnostic criteria of the American Rheumatology Society | 70.8±5.0 | 30.5±4.3 | 33 | 6 weeks | (iii) | Hydrotherapy:Twice a week | 4 |
|  |  |  |  | 71.0±5.2 | 30.0±5.2 | 32 |  |  | education | 4 |
| Fukuda  2011^37^ | Revista brasileira de ortopedia | Brazil | Kellgren Lawrence standard diagnosis | 63.0±9.0 | 30.0±3.5 | 25(20:5) | 3 weeks | (i) | Laser:27.0 J per time | 0 |
|  |  |  |  | 63.0±8.0 | 28.7±4.1 | 22(14:8) |  |  | placebo | 0 |
| Tascioglu  2004^38^ | SWISS MED WKLY | Turkey | Kellgren Lawrence standard diagnosis | 62.86±7.32 | 27.56±5.65 | 20(14:6) | 3 weeks | (ii)(iii) | Laser:3J | 0 |
|  |  |  |  | 59.92±7.59 | 28.63±6.48 | 20(15:5) |  |  | Laser:1.5J | 0 |
|  |  |  |  | 64.27±10.55 | 29.56±9.54 | 20(13:7) |  |  | Placebo | 0 |
| Robbins  2022^39^ | Clinical Biomechanic | Australia | Kellgren Lawrence standard diagnosis | 66.09±5.89 | 32.89±5.95 | 43(32:11) | 8 weeks | (ii) | Laser:27J | 0 |
|  |  |  |  | 62.44±3.34 | 31.69±3.79 | 43(38:5) |  |  | health education | 0 |
| Foroughi  2011^40^ | Clinical Biomechanic | Australia | Diagnostic criteria of the American Rheumatology Society | 66±8 | 31.4±5.4 | 20 | 6 months | (iii) | Resistance training:3 sets, 8 repetitions per set | 6 |
|  |  |  |  | 65±7 | 32.7±8.4 | 25 |  |  | Sham treatment | 3 |
| Jorge2015^41^ | CLINICAL REHABILITATION | Brazil | Diagnostic criteria of the American Rheumatology Society | 61.7±6.4 | 30.6±5.75 | 29(29:0) | 12 weeks | (ii)(iii) | Resistance training | 0 |
|  |  |  |  | 59.9±7.5 | 31.4±4.42 | 30(30:0) |  |  | No intervention | 3 |
| Pazit2018^42^ | Physical Therapy in Sport | Australia | Diagnostic criteria of the American Rheumatology Society | 67.78±6.28 | 28.2±5.6 | 9(4:5) | 8 weeks | (iii) | High speed resistance training | 1 |
|  |  |  |  | 70.44±7.83 | 28.4±3.9 | 9(4:5) |  |  | conventional therapy | 1 |
| Kuş2023^43^ | Clinical Rehabilitation | Turkey | Diagnostic criteria of the American Rheumatology Society | 58.54±7.05 | 30.53±3.86 | 24(16:8) | 8 weeks | (ii)(iii) | Proprioceptive motor training | 6 |
|  |  |  |  | 60.04±6.05 | 29.59±3.77 | 22(15:7) |  |  | Resistance training | 6 |
| Siriratna  2022^44^ | Pain Research and Management | Thailand | Diagnostic criteria of the American Rheumatology Society | 66.1±9.4 | 28.1±5.2 | 21(18:3) | 4-5 weeks | (ii)(iii) | Laser:562.50 J | 0 |
|  |  |  |  | 65.0±8.5 | 27.4±5.8 | 21(16:5） |  |  | Sham laser | 2 |
| Ye2020^45^ | Frontiers in Medicine | China | Diagnostic criteria of the American Rheumatology Society | 64.48±7.81 | 24.15±2.47 | 25(13:12) | 12 weeks | (iii) | Eight section Brocade Aerobic Exercise:40 minutes each session | 0 |
|  |  |  |  | 63.08±3.65 | 24.56±2.31 | 25（17:8） |  |  | No intervention | 0 |
| Assar2020^46^ | BMC Sports Science, Medicine and Rehabilitation | Iran | Diagnostic criteria of the American Rheumatology Society | 55.9±8.6 | 29.8±7.2 | 12 | 8 weeks | (ii)(iii) | Resistance training:Three times a week | 0 |
|  |  |  |  | 57.5±6.9 | 28.5±3.7 | 12 |  |  | Water sports:Three times a week | 0 |
| YILDIZ2015^47^ | Turkish Journal of Medical Sciences | Turkey | Kellgren Lawrence standard diagnosis | 56.13±6.61 | 32.31±5.23 | 30(25:5) | 2 weeks | (ii) | Continuous ultrasound:1 MHz | 0 |
|  |  |  |  | 54.63±6.53 | 31.15±4.68 | 30（24:6） |  |  | Pulse ultrasound:1MHz | 0 |
|  |  |  |  | 57.76±7.15 | 30.91±4.33 | 30(26:4) |  |  | Placebo ultrasound | 0 |
| Ince2023^48^ | Clinical Rehabilitation | Turkey | Diagnostic criteria of the American Rheumatology Society | 55.9±7.7 | 33.7±6.3 | 29 | 10 weeks | (ii) | Balance training:Three times a week | 1 |
|  |  |  |  | 56.7±7.5 | 32.8±5.4 | 30 |  |  | Proprioceptive training:20-30 minutes | 0 |
| Øiestad1  2023^49^ | BMC Musculoskeletal Disorders | Norway | Radiological examination | 57.6±6.6 | 28.9±4.3 | 54(30:24) | 12 weeks | (i)(ii) | strength training | 3 |
|  |  |  |  | 57.3±7.1 | 29.4±4.4 | 53(28:25） |  |  | Aerobic exercise:2-3 times per week | 2 |
|  |  |  |  | 57.8±7.4 | 28.4±4.1 | 54(24:30) |  |  | General nursing | 2 |
| Foroughi  2011^50^ | Clinical Biomechanics | Australia | Diagnostic criteria of the American Rheumatology Society | 64±7 | 32.6±6.9 | 18 | 6 months | (iii) | Resistance motion | 10 |
|  |  |  |  | 64±8 | 31.9±5.2 | 19 |  |  | Sham movement | 7 |
| Zhao2013^51^ | journal o f surg i cal r esearch | China | Diagnostic criteria of the American Rheumatology Society | 59.9±11.3 | 26.1±2.6 | 30(20:14) | 4 weeks | (iii) | shock wave:6 Hz/s | 4 |
|  |  |  |  | 61.8±9.8 | 25.6±2.5 | 31（25:6） |  |  | placebo | 5 |
| Takacs2017^52^ | Archives of Physical Medicine and Rehabilitation | Canada | Kellgren Lawrence standard diagnosis | 66.1±8.7 | 28.5±5.4 | 17(19:1) | 10 weeks | (i)(iii) | Balance training:Four times a week | 3 |
|  |  |  |  | 67.1±5.4 | 28.9±4.5 | 19（13:7） |  |  | No intervention | 1 |
| GIN2017^53^ | Ultrasound in Medicine and Biology | Turkey | Diagnostic criteria of the American Rheumatology Society | NA | NA | 30 | 2 weeks | (iii) | Ultrasonic:1 MHz | 2 |
|  |  |  |  | NA | NA | 32 |  |  | Sham ultrasound | 1 |
| BAKER2021^54^ | The Journal of Rheumatology | USA | Image findings | 69±6 | 31±4 | 19(17:6) | 16 weeks | (iii) | strength training | Eight people have exited in total |
|  |  |  |  | 68±6 | 32±5 | 19（19:4） |  |  | health education |  |
| Külcü2009^55^ | Turk J Rheumatol | Turkey | Diagnostic criteria of the American Rheumatology Society | 63.1±13.6 | NA | 15(13:2) | 3 weeks | (ii)(iii) | ultrasonic:1Mhz | 0 |
|  |  |  |  | 62±6 | NA | 15（12:3） |  |  | No intervention | 0 |
| Antúnez2021^56^ | Revista Española de Geriatría y Gerontología | Spain | Diagnostic criteria of the American Rheumatology Society | 82.21 | NA | 17 | 4 weeks | (ii)(iii) | Aerobic exercise:Twice a week, 50 minutes each time | 0 |
|  |  |  |  | 86.12 | NA | 14 |  |  | placebo | 0 |
| Nambi S2017^57^ | Lasers Med Sci | Saudi Arabia | Kellgren Lawrence standard diagnosis | 58±6 | 26.9±4.8 | 17 | 4 weeks | (ii) | Laser:1.5J | 0 |
|  |  |  |  | 60±8 | 28.3±3.5 | 17 |  |  | placebo | 0 |
| Almeida  2021^58^ | Clinical Rheumatology | Brazil | Diagnostic criteria of the American Rheumatology Society | 55.2±7.4 | 26±3.14 | 21(16:5) | 14 weeks | (iii) | strength training:Three times a week | 1 |
|  |  |  |  | 53.8±7.7 | 27±2.7 | 20（16:4） |  |  | Health education | 2 |
| Cherian  2015^59^ | J Knee Surg | USA | Kellgren Lawrence standard diagnosis | 55±12 | 32.5±3.77 | 13(11:2) | 3 months | (ii) | TENS:48-400 μs | Two people have exited in total |
|  |  |  |  | 54±9 | 34.8±9.21 | 10（7:3） |  |  | General nursing |  |
| Itoh2008^60^ | BioMed Central | Japan | Diagnostic criteria of the American Rheumatology Society | NA | NA | 6 | 5 weeks | (ii)(iii) | TENS:4.122 kHz | 2 |
|  |  |  |  | NA | NA | 6 |  |  | No intervention | 2 |
| Jan2008^61^ | Physical Therapy | China | Diagnostic criteria of the American Rheumatology Society | 63.3±6.6 | NA | 34(27:7) | 8 weeks | (iii) | High resistance motion | 3 |
|  |  |  |  | 61.8±7.1 | NA | 34（27:7） |  |  | Low resistance movement | 0 |
|  |  |  |  | 62.8±6.3 | NA | 30(25:5) |  |  | No intervention | 4 |
| Lee2009^62^ | Clinical Rehabilitation | China | Kellgren Lawrence standard diagnosis | 70.2±4.8 | 26±3.8 | 29(27:2) | 8 weeks | (iii) | Tai Chi Qigong:Twice a week | 1 |
|  |  |  |  | 66.9±6.0 | 26±2.8 | 15（14:1） |  |  | No intervention | 2 |
| Gomiero  2018^63^ | Sao Paulo Med J. | Brazil | Diagnostic criteria of the American Rheumatology Society | 61.6±6.8 | 24.1±3.8 | 30(30:2) | 16 weeks | (ii)(iii) | Proprioceptive movement | 2 |
|  |  |  |  | 61.8±6.4 | 23.6±3.5 | 32（31:1） |  |  | Resistance motion | 0 |
| Helianthi  2016^64^ | Acta Med Indones-Indones J Intern Med | Indonesia | Kellgren Lawrence standard diagnosis | 69±6 | 25.8±4.3 | 30(18:12) | 2 weeks | (ii) | Laser:50 mW | 1 |
|  |  |  |  | 68±5 | 26.3±4.3 | 29（24:5） |  |  | placebo | 2 |
| Gundog M  2012^65^ | American Journal of Physical Medicine & Rehabilitation | Turkey | Diagnostic criteria of the American Rheumatology Society | 59.6±8.4 | 28.1±3.5 | 15（12：3） | 3 weeks | (ii)(iii) | 40Hz interference current | 0 |
|  |  |  |  | 59.6±8.1 | 29.5±4.3 | 15（12:3） |  |  | 100Hz interference current | 0 |
|  |  |  |  | 60.2±8.6 | 28.7±4.5 | 15(12:3) |  |  | 180Hz interference current | 0 |
|  |  |  |  | 60.5±10.8 | 28.8±2.7 | 15(12:3) |  |  | Sham treatment | 0 |
| Samut2015^66^ | Modern Rheumatology | Turkey | Diagnostic criteria of the American Rheumatology Society | 57.57±5.79 | 33.94±7.33 | 14 | 6 weeks | (ii)(iii) | Aerobic exercise:Three times a week | 0 |
|  |  |  |  | 60.92±8.85 | 30.36±5.67 | 14 |  |  | health education | 0 |
| Song2022^67^ | Front. Aging Neurosci. | China | Image findings | 64.15±8.56 | 24.60±5.64 | 17 | 3 months | (iii) | Tai Chi :Three times a week | 2 |
|  |  |  |  | 64.15±8.56 | 24.37±2.71 | 18 |  |  | health education | 3 |
| Zhu2017^68^ | J Tradit Chin Med | China | Diagnostic criteria of the American Rheumatology Society | 64.6±3.4 | 25.2±3.5 | 21（21:0) | 24 weeks | (iii) | Tai Chi:Three times a week | 2 |
|  |  |  |  | 64.5±3.4 | 25±3.4 | 19（19:0） |  |  | health education | 4 |
| Tu2021^69^ | Arthritis & Rheumatology | China | Diagnostic criteria of the American Rheumatology Society | 62.7±6.6 | 25.4±2.9 | 151(119:32) | 8 weeks | (i)(iii) | Electroacupuncture:  40 minutes each time | 9 |
|  |  |  |  | 62.8±7.1 | 26±3.5 | 146（106:40） |  |  | Sham electroacupuncture | 14 |
| Bruce-Brand2012^70^ | BMC Musculoskeletal Disorders | Ireland | Kellgren Lawrence standard diagnosis | 63.4±5.9 | 33.9±8.3 | 10(4:6) | 6 weeks | (iii) | Resistance training:Three times a week | 4 |
|  |  |  |  | 65.2±3.1 | 31.7±4.1 | 6（3:3） |  |  | general nursing | 7 |
| ELGENDY  2022^71^ | BMC Musculoskeletal Disorders | Egypt | Kellgren Lawrence standard diagnosis | 48.66±8.55 | 31.33±2.25 | 15 | 4 weeks | (ii) | shock wave:5Hz | 0 |
|  |  |  |  | 55.13±6.68 | 30.8±2.48 | 15 |  |  | general nursing | 0 |
| Brismee  2006^72^ | Clinical Rehabilitation | USA | Diagnostic criteria of the American Rheumatology Society | 70.8±9.8 | 27.96±5.92 | 18(19:3) | 12 weeks | (ii)(iii) | Tai Chi:Three times a week | 4 |
|  |  |  |  | 68.8±8.9 | 27.7±6.57 | 13(15:4) |  |  | health education | 6 |
| Taglietti  2018^73^ | Clinical Rehabilitation | Brazil | Diagnostic criteria of the American Rheumatology Society | 67.3±5.9 | 29.2±0.8 | 28(23:8) | 8 weeks | (ii)(iii) | Water sports:Twice a week | 3 |
|  |  |  |  | 68.7±6.7 | 30.4±0.9 | 21(18:11) |  |  | health education | 8 |
| Topp2002^74^ | Arch Phys Med Rehabil | USA | According to reporting standards | 63.53±1.90 | NA | 32(21:11) | 16 weeks | (iii) | Equal length resistance motion:12 weeks | 0 |
|  |  |  |  | 65.57±1.82 | NA | 35(25:10) |  |  | Dynamic resistance motion | 0 |

Abbreviations: BMI, body mass index; NA, not available;TENS, Transcutaneous Electrical Neuromuscular Stimulation;(i),NRS；(ii), VAS; (iii), WOMAC;

**References**

1. Matthew WR,Nauris T,Stuart JS,et al.Efficacy of home-based kinesthesia, balance & agility exercise training among persons with symptomatic knee osteoarthritis.Journal of Sports Science and Medicine.(2012) 11, 751-758.

2. Messier SP, Mihalko SL, Beavers DP,et al. Effect of High-Intensity Strength Training on Knee Pain and Knee Joint Compressive Forces Among Adults With Knee Osteoarthritis: The START Randomized Clinical Trial. JAMA. 2021 Feb 16; 325(7):646-657. doi: 10.1001/jama.2021.0411.

3. Law PP, Cheing GL. Optimal stimulation frequency of transcutaneous electrical nerve stimulation on people with knee osteoarthritis. J Rehabil Med. 2004 Sep;36(5):220-5. doi: 10.1080/16501970410029834.

4. Bennell KL, Kyriakides M, Metcalf B, et al. Neuromuscular versus quadriceps strengthening exercise in patients with medial knee osteoarthritis and varus malalignment: a randomized controlled trial. Arthritis Rheumatol. 2014 Apr; 66(4):950-9. doi: 10.1002/art.38317.

5. Ahmed EE,²Tamer MA,³Nour A,et al.EFFECT OF SHOCK WAVE THERAPY VERSUS CORTICOSTEROID INJECTION IN MANAGEMENT OF KNEE OSTEOARTHRITIS.Int J Physiother. Vol 3(2), 246-251,April (2016).doi:10.15621/ijphy/2016/v3i2/94906

6. Chang TF, Liou TH, Chen CH, et al. Effects of elastic-band exercise on lower-extremity function among female patients with osteoarthritis of the knee. Disabil Rehabil. 2012;34(20):1727-35. doi: 10.3109/09638288.2012.660598.

7. Tsauo JY, Cheng PF, Yang RS. The effects of sensorimotor training on knee proprioception and function for patients with knee osteoarthritis: a preliminary report. Clin Rehabil. 2008 May;22(5):448-57. doi: 10.1177/0269215507084597.

8. Lund H, Weile U, Christensen R, Rostock B,et al. A randomized controlled trial of aquatic and land-based exercise in patients with knee osteoarthritis. J Rehabil Med. 2008 Feb;40(2):137-44. doi: 10.2340/16501977-0134.

9. Karakaş A, Dilek B, Şahin MA,et al. The effectiveness of pulsed ultrasound treatment on pain, function, synovial sac thickness and femoral cartilage thickness in patients with knee osteoarthritis: a randomized, double-blind clinical, controlled study. Clin Rehabil. 2020 Dec;34(12):1474-1484. doi: 10.1177/0269215520942953.

10. Sadeghi A, Rostami M, Khanlari Z, et al. Effectiveness of muscle strengthening exercises on the clinical outcomes of patients with knee osteoarthritis: A randomized four-arm controlled trial. Caspian J Intern Med. 2023 Summer;14(3):433-442. doi: 10.22088/cjim.14.3.433.

11. Yurtkuran M, Alp A, Konur S, Ozçakir S,et al. Laser acupuncture in knee osteoarthritis: a double-blind, randomized controlled study. Photomed Laser Surg. 2007 Feb;25(1):14-20. doi: 10.1089/pho.2006.1093.

12. Tascioglu F, Kuzgun S, Armagan O, et al. Short-term effectiveness of ultrasound therapy in knee osteoarthritis. J Int Med Res. 2010 Jul-Aug;38(4):1233-42. doi: 10.1177/147323001003800404.

13. McIlroy S, Sayliss L, Browning P,Bearne LM. Aquatic therapy for people with persistent kneepain: A feasibility study. Musculoskeletal Care. 2017;15:350–355.doi.org/10.1002/msc.1179.

14. DeVita P, Aaboe J, Bartholdy C, et al. Quadriceps-strengthening exercise and quadriceps and knee biomechanics during walking in knee osteoarthritis: A two-centre randomized controlled trial. Clin Biomech (Bristol, Avon). 2018 Nov;59:199-206. doi: 10.1016/j.clinbiomech.2018.09.016.

15. Ozgönenel L, Aytekin E, Durmuşoglu G. A double-blind trial of clinical effects of therapeutic ultrasound in knee osteoarthritis. Ultrasound Med Biol. 2009 Jan;35(1):44-9. doi: 10.1016/j.ultrasmedbio.2008.07.009.

16. Zhong Z, Liu B, Liu G, et al. A Randomized Controlled Trial on the Effects of Low-Dose Extracorporeal Shockwave Therapy in Patients With Knee Osteoarthritis. Arch Phys Med Rehabil. 2019 Sep; 100(9):1695-1702. doi: 10.1016/j.apmr.2019.04.020.

17. Hinman RS, McCrory P, Pirotta M, et al. Acupuncture for chronic knee pain: a randomized clinical trial. JAMA. 2014 Oct 1;312(13):1313-22. doi: 10.1001/jama.2014.12660.

18. An B, Dai K, Zhu Z, et al. Baduanjin alleviates the symptoms of knee osteoarthritis. J Altern Complement Med. 2008 Mar;14(2):167-74. doi: 10.1089/acm.2007.0600.

19. Atamaz FC, Durmaz B, Baydar M, et al. Comparison of the efficacy of transcutaneous electrical nerve stimulation, interferential currents, and shortwave diathermy in knee osteoarthritis: a double-blind, randomized, controlled, multicenter study. Arch Phys Med Rehabil. 2012 May;93(5):748-56. doi: 10.1016/j.apmr.2011.11.037.

20. Hammam RF, Kamel RM, Draz AH, et al. Comparison of the effects between low- versus medium-energy radial extracorporeal shock wave therapy on knee osteoarthritis: A randomised controlled trial. J Taibah Univ Med Sci. 2020 May 15;15(3):190-196. doi: 10.1016/j.jtumed.2020.04.003.

21. Artuç ŞE, Uçkun AÇ, Si VAS FA,et al. Comparison of the effects of transcutaneous electrical nerve stimulation and interferential current therapies in central sensitization in patients with knee osteoarthritis. Korean J Pain. 2023 Jul 1; 36(3):392-403. doi: 10.3344/kjp.23118.

22. Cheing GL, Hui-Chan CW, Chan KM. Does four weeks of TENS and/or isometric exercise produce cumulative reduction of osteoarthritic knee pain? Clin Rehabil. 2002 Nov;16(7):749-60. doi: 10.1191/0269215502cr549oa.

23. Weng MC, Lee CL, Chen CH, et al. Effects of different stretching techniques on the outcomes of isokinetic exercise in patients with knee osteoarthritis. Kaohsiung J Med Sci. 2009 Jun;25(6):306-15. doi: 10.1016/S1607-551X(09)70521-2.

24. Mostafa MSEM, Hamada HA, Kadry AM,et al. Effect of High-Power Laser Therapy Versus Shock Wave Therapy on Pain and Function in Knee Osteoarthritis Patients: A Randomized Controlled Trial. Photobiomodul Photomed Laser Surg. 2022 Mar;40(3):198-204. doi: 10.1089/photob.2021.0136.

25. Draper DO, Klyve D, Ortiz R, Best TM. Effect of low-intensity long-duration ultrasound on the symptomatic relief of knee osteoarthritis: a randomized, placebo-controlled double-blind study. J Orthop Surg Res. 2018 Oct 16;13(1):257. doi: 10.1186/s13018-018-0965-0.

26. Alghadir A, Omar MT, Al-Askar AB,et al. Effect of low-level laser therapy in patients with chronic knee osteoarthritis: a single-blinded randomized clinical study. Lasers Med Sci. 2014 Mar;29(2):749-55. doi: 10.1007/s10103-013-1393-3.

27. Reichenbach S, Jüni P, Hincapié CA, et al. Effect of transcutaneous electrical nerve stimulation (TENS) on knee pain and physical function in patients with symptomatic knee osteoarthritis: the ETRELKA randomized clinical trial. Osteoarthritis Cartilage. 2022 Mar; 30(3):426-435. doi: 10.1016/j.joca.2021.10.015.

28. Özgönenel L, Okur SÇ, Dogan YP, Çaglar NS. Effectiveness of therapeutic ultrasound on clinical parameters and ultrasonographic cartilage thickness in knee osteoarthritis: A double-blind trial. J Med Ultrasound 2018;26:194-9.DOI:

10.4103/JMU.JMU_21_18.

29. Cho SJ, Yang JR, Yang HS, et al. Effects of Extracorporeal Shockwave Therapy in Chronic Stroke Patients With Knee Osteoarthritis: A Pilot Study. Ann Rehabil Med. 2016 Oct; 40(5):862-870. doi: 10.5535/arm.2016.40.5.862.

30. Lv ZT, Shen LL, Zhu B, et al. Effects of intensity of electroacupuncture on chronic pain in patients with knee osteoarthritis: a randomized controlled trial. Arthritis Res Ther. 2019 May 14;21(1):120. doi: 10.1186/s13075-019-1899-6.

31. Mascarin NC, Vancini RL, Andrade ML, et al. Effects of kinesiotherapy, ultrasound and electrotherapy in management of bilateral knee osteoarthritis: prospective clinical trial. BMC Musculoskelet Disord. 2012 Sep 22;13:182. doi: 10.1186/1471-2474-13-182.

32. Lin DH, Lin CH, Lin YF, Jan MH. Efficacy of 2 non-weight-bearing interventions, proprioception training versus strength training, for patients with knee osteoarthritis: a randomized clinical trial. J Orthop Sports Phys Ther. 2009 Jun;39(6):450-7. doi: 10.2519/jospt.2009.2923.

33. Nazari A, Moezy A, Nejati P, et al. Efficacy of high-intensity laser therapy in comparison with conventional physiotherapy and exercise therapy on pain and function of patients with knee osteoarthritis: a randomized controlled trial with 12-week follow up. Lasers Med Sci. 2019 Apr;34(3):505-516. doi: 10.1007/s10103-018-2624-4.

34. Thais VD,Cintia PS,Elirez BS.Neuromuscular electrical stimulation, exercises against resistance, muscle strength, pain, and motor function in patients with primary osteoarthritis of the knee.Fisioter Mov. 2013;26(4):777-789.

35. Wang TS, Guo P, Li G, Wang JW. Extracorporeal Shockwave Therapy for Chronic Knee Pain: A Multicenter, Randomized Controlled Trial. Altern Ther Health Med. 2020 Mar;26(2):34-37.

36. Dias JM, Cisneros L, Dias R, et al. Hydrotherapy improves pain and function in older women with knee osteoarthritis: a randomized controlled trial. Braz J Phys Ther. 2017 Nov-Dec;21(6):449-456. doi: 10.1016/j.bjpt.2017.06.012.

37. Fukuda VO, Fukuda TY, Guimarães M, et al. SHORT-TERM EFFICACY OF LOW-LEVEL LASER THERAPY IN PATIENTS WITH KNEE OSTEOARTHRITIS: A RANDOMIZED PLACEBO-CONTROLLED, DOUBLE-BLIND CLINICAL TRIAL. Rev Bras Ortop. 2015 Dec 6;46(5):526-33. doi: 10.1016/S2255-4971(15)30407-9.

38. Tascioglu F, Armagan O, Tabak Y, et al. Low power laser treatment in patients with knee osteoarthritis. Swiss Med Wkly. 2004 May 1;134(17-18):254-8. doi: 10.4414/smw.2004.10518.

39. Robbins SR, Alfredo PP, Junior WS, et al. Low-level laser therapy and static stretching exercises for patients with knee osteoarthritis: A randomised controlled trial. Clin Rehabil. 2022 Feb;36(2):204-213. doi: 10.1177/02692155211047017.

40 .Foroughi N, Smith RM, Lange AK,et al. Lower limb muscle strengthening does not change frontal plane moments in women with knee osteoarthritis: A randomized controlled trial. Clin Biomech (Bristol, Avon). 2011 Feb;26(2):167-74. doi: 10.1016/j.clinbiomech.2010.08.011.

41. Jorge RT, Souza MC, Chiari A, et al. Progressive resistance exercise in women with osteoarthritis of the knee: a randomized controlled trial. Clin Rehabil. 2015 Mar;29(3):234-43. doi: 10.1177/0269215514540920.

42. Levinger Pazit, Dunn Jeremy, Bifera Nancy, et al, Safety and feasibility of high speed resistance training with and without balance exercises for knee osteoarthritis: a pilot randomised controlled trial, Physical Therapy in Sport (2018), doi: 10.1016/j.ptsp.2018.10.001.

43. Kuş G, Tarakçı E, Razak Ozdincler A, et al. Sensory-motor training versus resistance training in the treatment of knee osteoarthritis: A randomized controlled trial. Clin Rehabil. 2023 May;37(5):636-650. doi: 10.1177/02692155221137642.

44. Siriratna P, Ratanasutiranont C, Manissorn T, et al. Short-Term Efficacy of High-Intensity Laser Therapy in Alleviating Pain in Patients with Knee Osteoarthritis: A Single-Blind Randomised Controlled Trial. Pain Res Manag. 2022 Oct 21;2022:1319165. doi: 10.1155/2022/1319165.

45. Ye J, Simpson MW, Liu Y,et al. The Effects of Baduanjin Qigong on Postural Stability, Proprioception, and Symptoms of Patients With Knee Osteoarthritis: A Randomized Controlled Trial. Front Med (Lausanne). 2020 Jan 10;6:307. doi: 10.3389/fmed.2019.00307.

46. Assar S, Gandomi F, Mozafari M, et al. The effect of Total resistance exercise vs. aquatic training on self-reported knee instability, pain, and stiffness in women with knee osteoarthritis: a randomized controlled trial. BMC Sports Sci Med Rehabil. 2020 Apr 29;12:27. doi: 10.1186/s13102-020-00175-y.

47. YILDIZ, SERAP KAPCI; ÖZKAN, et al "The effectiveness of ultrasound treatment for the management of kneeosteoarthritis: a randomized, placebo-controlled, double-blind study," Turkish Journal of Medical Sciences: Vol. 45: No. 6, Article 1. doi.org/10.3906/sag-1408-81.

48. Ince B, Goksel Karatepe A, Akcay S, et al. The efficacy of balance and proprioception exercises in female patients with knee osteoarthritis: A randomized controlled study. Clin Rehabil. 2023 Jan;37(1):60-71. doi: 10.1177/02692155221111929.

49. Øiestad BE, Årøen A, Røtterud JH,et al. The efficacy of strength or aerobic exercise on quality of life and knee function in patients with knee osteoarthritis. A multi-arm randomized controlled trial with 1-year follow-up. BMC Musculoskelet Disord. 2023 Sep 8;24(1):714. doi: 10.1186/s12891-023-06831-x.

50. Foroughi N, Smith RM, Lange AK, et al. Progressive resistance training and dynamic alignment in osteoarthritis: A single-blind randomised controlled trial. Clin Biomech (Bristol, Avon). 2011 Jan;26(1):71-7. doi: 10.1016/j.clinbiomech.2010.08.013.

51. Zhao Z, Jing R, Shi Z,et al. Efficacy of extracorporeal shockwave therapy for knee osteoarthritis: a randomized controlled trial. J Surg Res. 2013 Dec;185(2):661-6. doi: 10.1016/j.jss.2013.07.004.

52. Takacs J, Krowchuk NM, Garland SJ, et al. Dynamic Balance Training Improves Physical Function in Individuals With Knee Osteoarthritis: A Pilot Randomized Controlled Trial. Arch Phys Med Rehabil. 2017 Aug;98(8):1586-1593. doi: 10.1016/j.apmr.2017.01.029.

53. Yeğin T, Altan L, Kasapoğlu Aksoy M. The Effect of Therapeutic Ultrasound on Pain and Physical Function in Patients with Knee Osteoarthritis. Ultrasound Med Biol. 2017 Jan;43(1):187-194. doi: 10.1016/j.ultrasmedbio.2016.08.035.

54. Baker KR, Nelson ME, Felson DT, et al. The efficacy of home based progressive strength training in older adults with knee osteoarthritis: a randomized controlled trial. J Rheumatol. 2001 Jul;28(7):1655-65. PMID: 11469475.

55. Duygu GK,Gülçin G,Elif ÇA,Short-Term Efficacy of Pulsed Electromagnetic Field Therapy on Pain and Functional Level in Knee Osteoarthritis: A Randomized Controlled Study.Turk J Rheumatol 2009; 24: 144-8.

56. Espejo Antúnez L, Cardero Durán MÁ, Caro Puértolas B, et al. Efectos del ejercicio físico en la funcionalidad y calidad de vida en mayores institucionalizados diagnosticados de gonartrosis [Effects of exercise on the function and quality of life in the institutionalised elderly diagnosed with gonarthrosis]. Rev Esp Geriatr Gerontol. 2012 Nov-Dec;47(6):262-5. Spanish. doi: 10.1016/j.regg.2011.06.011.

57. S GN, Kamal W, George J, Manssor E. Radiological and biochemical effects (CTX-II, MMP-3, 8, and 13) of low-level laser therapy (LLLT) in chronic osteoarthritis in Al-Kharj, Saudi Arabia. Lasers Med Sci. 2017 Feb;32(2):297-303. doi: 10.1007/s10103-016-2114-5.

58. de Almeida AC, Aily JB, Pedroso MG, et al. Reductions of cardio VAS cular and metabolic risk factors after a 14-week periodized training model in patients with knee osteoarthritis: a randomized controlled trial. Clin Rheumatol. 2021 Jan; 40(1):303-314. doi: 10.1007/s10067-020-05213-1.

59. Cherian JJ, Kapadia BH, Bhave A, et al. Use of Transcutaneous Electrical Nerve Stimulation Device in Early Osteoarthritis of the Knee. J Knee Surg. 2015 Aug;28(4):321-7. doi: 10.1055/s-0034-1389160.

60. Itoh K, Hirota S, Katsumi Y, et al. A pilot study on using acupuncture and transcutaneous electrical nerve stimulation (TENS) to treat knee osteoarthritis (OA). Chin Med. 2008 Feb 29;3:2. doi: 10.1186/1749-8546-3-2.

61. Jan MH, Lin JJ, Liau JJ, et al. Investigation of clinical effects of high- and low-resistance training for patients with knee osteoarthritis: a randomized controlled trial. Phys Ther. 2008 Apr;88(4):427-36. doi: 10.2522/ptj.20060300.

62. Lee HJ, Park HJ, Chae Y, et al. Tai Chi Qigong for the quality of life of patients with knee osteoarthritis: a pilot, randomized, waiting list controlled trial. Clin Rehabil. 2009 Jun;23(6):504-11. doi: 10.1177/0269215508101746.

63. Gomiero AB, Kayo A, Abraão M, Peccin MS, Grande AJ, Trevisani VF. Sensory-motor training versus resistance training among patients with knee osteoarthritis: randomized single-blind controlled trial. Sao Paulo Med J. 2018 Jan-Feb;136(1):44-50. doi: 10.1590/1516-3180.2017.0174100917. Epub 2017 Dec 7. PMID: 29236934.

64. Helianthi DR, Simadibrata C, Srilestari A, et al. Pain Reduction After Laser Acupuncture Treatment in Geriatric Patients with Knee Osteoarthritis: a Randomized Controlled Trial. Acta Med Indones. 2016 Apr;48(2):114-21. PMID: 27550880.

65. Gundog M, Atamaz F, Kanyilmaz S, et al. Interferential current therapy in patients with knee osteoarthritis: comparison of the effectiveness of different amplitude-modulated frequencies. Am J Phys Med Rehabil. 2012 Feb;91(2):107-13. doi: 10.1097/PHM.0b013e3182328687.

66. Samut G, Dinçer F, Özdemir O. The effect of isokinetic and aerobic exercises on serum interleukin-6 and tumor necrosis factor alpha levels, pain, and functional activity in patients with knee osteoarthritis. Mod Rheumatol. 2015;25(6):919-24. doi: 10.3109/14397595.2015.1038425.

67. Song J, Wei L, Cheng K, et al (2022) The Effect of Modified Tai Chi Exercises on the Physical Function and Quality of Life in Elderly Women With Knee Osteoarthritis. Front. Aging Neurosci. 14:860762. doi: 10.3389/fnagi.2022.860762.

68. Zhu Q, Huang L, Wu X, et al. Effect of Taijiquan practice versus wellness education on knee proprioception in patients with knee osteoarthritis: a randomized controlled trial. J Tradit Chin Med. 2017 Dec;37(6):774-781. PMID: 32188186.

69. Tu JF, Yang JW, Shi GX, et al. Efficacy of Intensive Acupuncture Versus Sham Acupuncture in Knee Osteoarthritis: A Randomized Controlled Trial. Arthritis Rheumatol. 2021 Mar;73(3):448-458. doi: 10.1002/art.41584.

70. Bruce-Brand RA, Walls RJ, Ong JC, et al. Effects of home-based resistance training and neuromuscular electrical stimulation in knee osteoarthritis: a randomized controlled trial. BMC Musculoskelet Disord. 2012 Jul 3;13:118. doi: 10.1186/1471-2474-13-118.

71. Mohamed HE, Sara AE, Mohamed SE et al.Efficacy Of ShockwaveTherapy Versus Intra-Articular Platelet-Rich Plasma Injection In Management Of Knee Osteoarthritis: A Randomized Controlled Trial. International Journal of Pharmaceutical Research, 2020, 12(04).DOI: 10.31838/ijpr/2020.12.04.589.

72. Brismée JM, Paige RL, Chyu MC, et al. Group and home-based tai chi in elderly subjects with knee osteoarthritis: a randomized controlled trial. Clin Rehabil. 2007 Feb;21(2):99-111. doi: 10.1177/0269215506070505.

73. Taglietti M, Facci LM, Trelha CS,et al. Effectiveness of aquatic sportss compared to patient-education on health status in individuals with knee osteoarthritis: a randomized controlled trial. Clin Rehabil. 2018 Jun;32(6):766-776. doi: 10.1177/0269215517754240.

74. Topp R, Woolley S, Hornyak J 3rd, et al. The effect of dynamic versus isometric resistance training on pain and functioning among adults with osteoarthritis of the knee. Arch Phys Med Rehabil. 2002 Sep;83(9):1187-95. doi: 10.1053/apmr.2002.33988.

**Appendix 3: Risk of bias of randomized clinical trials**

Abbreviations: L, low bias; U, unclear bias; H, high bias.

**Table S3.1:** The Cochrane bias risk Tool 2.0 was used to assess the bias risk of randomized clinical trials with NRS outcome.

| ID | Random sequence generation | Allocation concealment | Blinding of participants and personnel | Blinding of outcome assessment | Incomplete outcome data | Selection of the  reported result | other bias |
| --- | --- | --- | --- | --- | --- | --- | --- |
| Wang2020 | L | U | L | L | L | U | L |
| Draper2018 | L | U | L | L | L | U | L |
| Hinman2014 | L | U | H | U | L | U | L |
| Fukuda2011 | L | L | L | L | L | U | L |
| Øiestad2023 | L | L | U | L | L | U | L |
| Lv2019 | L | L | L | L | L | U | L |
| Takacs2017 | L | L | H | L | L | U | L |
| Rheumatology2021 | L | L | H | L | L | U | L |

**Table S3.2:** The Cochrane bias risk Tool 2.0 was used to assess the bias risk of randomized clinical trials with VAS outcome.

| ID | Random sequence generation | Allocation concealment | Blinding of participants and personnel | Blinding of outcome assessment | Incomplete outcome data | Selection of the  reported result | other bias |
| --- | --- | --- | --- | --- | --- | --- | --- |
| Sadeghi2023 | L | L | L | L | L | U | L |
| YURTKURAN2007 | L | U | L | L | L | U | L |
| TASCIOGLU2010 | U | L | L | L | L | U | L |
| Lund2008 | U | L | U | U | H | U | U |
| McIlroy 2017 | L | U | U | L | L | U | L |
| ÖZGÖNENEL2009 | L | U | L | L | L | U | L |
| Zhong2019 | L | U | L | L | L | U | L |
| Atamaz2012 | L | U | L | L | H | U | L |
| Kuş2023 | L | L | U | U | L | U | U |
| Øiestad2023 | L | L | U | L | L | U | L |
| Ince2023 | L | L | L | L | L | U | L |
| Karakaş2020 | L | U | L | L | H | U | L |
| Assar2020 | L | L | L | L | L | U | L |
| YILDIZ2015 | L | L | L | U | L | U | L |
| Siriratna2022 | L | L | L | U | L | U | L |
| Jorge2015 | L | L | H | L | L | U | L |
| Tascioglu2004 | L | U | U | L | U | U | L |
| Nazari2019 | L | U | L | L | L | U | L |
| Cho2016 | L | L | L | L | L | U | L |
| Mascarin2012 | U | U | U | L | L | U | L |
| Weng2009 | L | L | L | L | H | U | L |
| Lv2019 | L | L | L | L | L | U | L |
| Özgönenel2018 | L | U | L | L | U | U | L |
| Cheing2002 | U | U | U | U | L | U | U |
| Alghadir2014 | L | L | H | U | L | U | L |
| Mostafa2022 | L | L | L | L | L | U | L |
| Artuç2023 | L | U | L | L | L | U | L |
| Hammam2020 | L | L | U | U | L | U | L |
| Law2004 | L | U | H | L | U | U | L |
| Elerian2016 | L | U | U | U | U | U | U |
| Bennell2014 | L | L | H | L | H | U | L |
| Robbins2022 | L | L | H | L | L | U | L |
| Reichenbach  2022 | L | L | L | L | L | U | L |
| Külcü2009 | U | U | H | H | L | U | L |
| Gerontología2012 | L | L | L | U | L | U | L |
| Nambi 2017 | L | L | U | L | L | U | L |
| Cherian2015 | L | L | H | U | L | U | L |
| Itoh2008 | L | L | U | U | L | U | L |
| Gomiero2017 | L | L | H | L | L | U | L |
| Helianthi2016 | L | L | L | L | L | U | L |
| Gundog2012 | U | U | U | L | L | U | L |
| Samut2015 | U | U | U | U | L | U | L |
| ELGENDY2020 | L | L | U | U | L | U | L |
| Brisme´e2007 | U | U | H | L | L | U | L |
| Taglietti2018 | L | L | H | L | H | U | L |

**Table S3.3:** The Cochrane bias risk Tool 2.0 was used to assess the bias risk of randomized clinical trials with WOMAC outcome.

| ID | Random sequence generation | Allocation concealment | Blinding of participants and personnel | Blinding of outcome assessment | Incomplete outcome data | Selection of the  reported result | other bias |
| --- | --- | --- | --- | --- | --- | --- | --- |
| YURTKURAN2007 | L | U | L | L | L | U | L |
| TASCIOGLU2010 | U | L | L | L | L | U | L |
| McIlroy2017 | L | U | U | L | L | U | L |
| ÖZGÖNENEL2009 | L | U | L | L | L | U | L |
| Zhong2019 | L | U | L | L | L | U | L |
| Hinman2014 | L | H | L | L | H | U | U |
| Atamaz2012 | L | U | L | L | H | U | L |
| Kuş2023 | L | L | U | U | L | U | U |
| Pazit2018 | L | L | U | U | L | U | L |
| Ye2020 | L | L | L | L | L | U | L |
| Karakaş2020 | L | U | L | L | H | U | L |
| Siriratna2022 | L | L | L | U | L | U | L |
| Jorge2015 | L | L | H | L | L | U | L |
| Foroughi2011 | L | L | L | L | L | U | L |
| Tascioglu2004 | L | U | U | L | U | U | L |
| Diasa2017 | L | L | L | L | L | U | L |
| Nazari2019 | L | U | L | L | L | U | L |
| Dadalto2013 | L | H | L | L | U | U | L |
| Wang2020 | L | U | L | L | L | U | L |
| Mascarin2012 | U | U | U | L | L | U | L |
| LIN2009 | L | L | H | L | L | U | L |
| Lv2019 | L | L | L | L | L | U | L |
| Reichenbach2022 | L | L | L | L | L | U | L |
| Özgönenel2018 | L | U | L | L | U | U | L |
| Draper2018 | L | U | L | L | L | U | L |
| Alghadir2014 | L | L | H | U | L | U | L |
| Mostafa2022 | L | L | L | L | L | U | L |
| Artuç2023 | L | U | L | L | L | U | L |
| AN2008 | U | U | U | U | H | U | L |
| DeVita2018 | L | U | U | U | L | U | L |
| Tsauo2008 | L | H | U | L | H | U | L |
| Chang2012 | U | U | U | U | L | U | L |
| Elerian2016 | L | U | U | U | U | U | U |
| Bennell2014 | L | L | H | L | H | U | L |
| Rogers2012 | L | U | L | U | L | U | L |
| Messier2021 | U | U | U | U | L | U | L |
| Assar2020 | L | L | H | L | L | U | L |
| Foroughi2011 | U | U | L | L | H | U | L |
| Zhao2013 | L | H | L | L | L | U | L |
| Takacs2017 | L | L | H | L | L | U | L |
| GIN2017 | U | U | U | L | L | U | L |
| BAKER2021 | L | L | U | L | L | U | L |
| Külcü2009 | U | U | H | H | L | U | L |
| Gerontología2012 | L | L | L | U | L | U | L |
| Almeida2021 | L | L | U | L | L | U | L |
| Itoh2008 | L | L | U | U | L | U | L |
| Jan-15 | L | L | H | L | L | U | L |
| Lee2009 | L | L | H | L | L | U | L |
| Gomiero2017 | L | L | H | L | L | U | L |
| Gundog2012 | U | U | U | L | L | U | L |
| Samut2015 | U | U | U | U | L | U | L |
| Song2022 | L | L | H | L | L | U | L |
| Zhu 2017 | L | L | U | U | L | U | L |
| Rheumatology2021 | L | L | H | L | L | U | L |
| Bruce-Brand2012 | L | L | H | L | H | U | L |
| Brisme´e2007 | U | U | H | L | L | U | L |
| Taglietti2018 | L | L | H | L | H | U | L |
| Topp2002 | U | U | U | U | L | U | L |

**Appendix 4: CINeMA Assessment**

We use the CINeMA framework to evidence certainty, assessing it for each network estimate based on the following criteria:

**Within study bias:** We classified the overall risk of bias for each study as low risk of bias, the risk of bias as moderate when none of the four assessed risk of bias items were rated as high risk, and the risk of bias as high when one or both items were rated as high risk.

**Imprecision:** We use the CINeMA website to grade the accuracy of each comparison.

**Inconsistency:** For inconsistency, we looked at the results for node splitting (Appendix 5).

**Abbreviations:** TENS, Transcutaneous Electrical Nerve Stimulation; NEXA, Neuromuscular exercise.

**Heterogeneity:** We assessed the degree of worry by comparing clinical reasoning based on 95% confidence intervals (CIs) while applying the same clinical reasoning framework as for inaccuracy.

**Table S4.1:** CINeMA results of VAS pain during walking at less than 1 month.

| **Comparison** | **Within-study bias** | **Reporting bias** | **Indirectness** | **Imprecision** | **Heterogeneity** | **Incoherence** | **Confidence rating** |
| --- | --- | --- | --- | --- | --- | --- | --- |
| Aerobic exercise: General nursing | Some concerns | Low risk | No concerns | Some concerns | Some concerns | No concerns | Low |
| Aquatic sports: General nursing | Some concerns | Low risk | No concerns | Some concerns | Some concerns | No concerns | Low |
| Aquatic sports: Placebo | Some concerns | Low risk | No concerns | Some concerns | Some concerns | No concerns | Low |
| Aquatic sports: Strength training | No concerns | Low risk | No concerns | Major concerns | No concerns | No concerns | Low |
| General nursing: Laser | Some concerns | Low risk | No concerns | No concerns | Major concerns | No concerns | Low |
| Laser: Placebo | No concerns | Low risk | No concerns | No concerns | Some concerns | No concerns | Moderate |
| Placebo: Shockwave | No concerns | Low risk | No concerns | Some concerns | Some concerns | No concerns | Low |
| Placebo: Strength training | Some concerns | Low risk | No concerns | No concerns | Major concerns | No concerns | Low |
| Placebo: TENS | Some concerns | Low risk | No concerns | Some concerns | Some concerns | No concerns | Low |
| Shockwave: TENS | Some concerns | Low risk | No concerns | Major concerns | No concerns | No concerns | Low |
| Aerobic exercise: Aquatic sports | Some concerns | Low risk | No concerns | Major concerns | No concerns | No concerns | Low |
| Aerobic exercise: Laser | Some concerns | Low risk | No concerns | Major concerns | No concerns | No concerns | Low |
| Aerobic exercise: Placebo | Some concerns | Low risk | No concerns | Some concerns | Some concerns | No concerns | Low |
| Aerobic exercise: Shockwave | Some concerns | Low risk | No concerns | Major concerns | No concerns | No concerns | Low |
| Aerobic exercise: Strength training | Some concerns | Low risk | No concerns | Major concerns | No concerns | No concerns | Low |

**Table S4.2:** CINeMA results of VAS pain during walking at 1-3 months.

| **Comparison** | **Within-study bias** | **Reporting bias** | **Indirectness** | **Imprecision** | **Heterogeneity** | **Incoherence** | **Confidence rating** |
| --- | --- | --- | --- | --- | --- | --- | --- |
| Aerobic exercise: General nursing | Some concerns | Low risk | No concerns | Major concerns | No concerns | No concerns | Low |
| Aquatic sports: General nursing | Some concerns | Low risk | No concerns | Major concerns | No concerns | No concerns | Low |
| Aquatic sports: Placebo | Some concerns | Low risk | No concerns | Major concerns | No concerns | No concerns | Low |
| Aquatic sports: Strength training | No concerns | Low risk | No concerns | Major concerns | No concerns | No concerns | Low |
| General nursing: Laser | Some concerns | Low risk | No concerns | No concerns | Major concerns | No concerns | Low |
| Laser: Placebo | No concerns | Low risk | No concerns | No concerns | Major concerns | No concerns | Low |
| Placebo: Shockwave | No concerns | Low risk | No concerns | Major concerns | No concerns | No concerns | Low |
| Placebo: Strength training | Some concerns | Low risk | No concerns | No concerns | Major concerns | No concerns | Low |
| Placebo: TENS | Some concerns | Low risk | No concerns | Major concerns | No concerns | No concerns | Low |
| Shockwave: TENS | Some concerns | Low risk | No concerns | Major concerns | No concerns | No concerns | Low |
| Aerobic exercise: Aquatic sports | Some concerns | Low risk | No concerns | Major concerns | No concerns | No concerns | Low |
| Aerobic exercise: Laser | Some concerns | Low risk | No concerns | Major concerns | No concerns | No concerns | Low |
| Aerobic exercise: Placebo | Some concerns | Low risk | No concerns | Major concerns | No concerns | No concerns | Low |
| Aerobic exercise: Shockwave | Some concerns | Low risk | No concerns | Major concerns | No concerns | No concerns | Low |
| Aerobic exercise: Strength training | Some concerns | Low risk | No concerns | Major concerns | No concerns | No concerns | Low |
| Aerobic exercise: TENS | Some concerns | Low risk | No concerns | Major concerns | No concerns | No concerns | Low |
| Aquatic sports: Laser | Some concerns | Low risk | No concerns | Major concerns | No concerns | No concerns | Low |
| Aquatic sports: Shockwave | Some concerns | Low risk | No concerns | Major concerns | No concerns | No concerns | Low |
| Aquatic sports: TENS | Some concerns | Low risk | No concerns | Major concerns | No concerns | No concerns | Low |
| General nursing: Placebo | Some concerns | Low risk | No concerns | Major concerns | No concerns | No concerns | Low |
| General nursing: Shockwave | No concerns | Low risk | No concerns | Major concerns | No concerns | No concerns | Low |
| General nursing: Strength training | Some concerns | Low risk | No concerns | Major concerns | No concerns | No concerns | Low |
| General nursing: TENS | Some concerns | Low risk | No concerns | Major concerns | No concerns | No concerns | Low |
| Laser: Shockwave | No concerns | Low risk | No concerns | Major concerns | No concerns | No concerns | Low |
| Laser: Strength training | No concerns | Low risk | No concerns | Major concerns | No concerns | No concerns | Low |
| Laser: TENS | No concerns | Low risk | No concerns | Major concerns | No concerns | No concerns | Low |
| Shockwave: Strength training | No concerns | Low risk | No concerns | Major concerns | No concerns | No concerns | Low |
| Strength training: TENS | Some concerns | Low risk | No concerns | Major concerns | No concerns | No concerns | Low |

**Table S4.3:** CINeMA results of VAS pain during walking at more than 3 months.

| **Comparison** | **Within-study bias** | **Reporting bias** | **Indirectness** | **Imprecision** | **Heterogeneity** | **Incoherence** | **Confidence rating** |
| --- | --- | --- | --- | --- | --- | --- | --- |
| Aerobic exercise: General nursing | No concerns | Low risk | No concerns | No concerns | No concerns | No concerns | High |
| Aerobic exercise: Strength training | No concerns | Low risk | No concerns | No concerns | Some concerns | No concerns | Moderate |
| Aquatic sports: General nursing | Major concerns | Low risk | No concerns | No concerns | No concerns | No concerns | Low |
| Aquatic sports: Placebo | Some concerns | Low risk | No concerns | No concerns | Some concerns | No concerns | Low |
| Balance training: Strength training | Some concerns | Low risk | No concerns | Some concerns | No concerns | Some concerns | Low |
| General nursing: Strength training | No concerns | Low risk | No concerns | No concerns | Some concerns | No concerns | Moderate |
| General nursing: TENS | Some concerns | Low risk | No concerns | No concerns | Some concerns | Major concerns | Low |
| Laser: Placebo | Some concerns | Low risk | No concerns | No concerns | Some concerns | Some concerns | Low |
| NEXA: Strength training | Some concerns | Low risk | No concerns | No concerns | Some concerns | Some concerns | Low |
| Placebo: Strength training | Some concerns | Low risk | No concerns | No concerns | Some concerns | No concerns | Low |
| Placebo: TENS | No concerns | Low risk | No concerns | No concerns | No concerns | Some concerns | Moderate |
| Aerobic exercise: Aquatic sports | Some concerns | Low risk | No concerns | No concerns | Some concerns | Some concerns | Low |
| Aerobic exercise: Balance training | Some concerns | Low risk | No concerns | Some concerns | Some concerns | Some concerns | Low |
| Aerobic exercise: Laser | Some concerns | Low risk | No concerns | Some concerns | Some concerns | Some concerns | Low |
| Aerobic exercise: NEXA | Some concerns | Low risk | No concerns | Some concerns | No concerns | Some concerns | Low |
| Aerobic exercise: Placebo | Some concerns | Low risk | No concerns | Some concerns | No concerns | Some concerns | Low |
| Aerobic exercise: TENS | Some concerns | Low risk | No concerns | No concerns | Some concerns | Some concerns | Low |
| Aquatic sports: Balance training | Some concerns | Low risk | No concerns | Some concerns | No concerns | Some concerns | Low |
| Aquatic sports: Laser | Some concerns | Low risk | No concerns | Some concerns | No concerns | Some concerns | Low |
| Aquatic sports: NEXA | Some concerns | Low risk | No concerns | Some concerns | Some concerns | Some concerns | Low |
| Aquatic sports: Strength training | Some concerns | Low risk | No concerns | No concerns | No concerns | Some concerns | Low |
| Aquatic sports: TENS | Some concerns | Low risk | No concerns | Some concerns | No concerns | Some concerns | Low |
| Balance training: General nursing | Some concerns | Low risk | No concerns | Some concerns | Some concerns | Some concerns | Low |
| Balance training: Laser | Some concerns | Low risk | No concerns | Major concerns | No concerns | Some concerns | Low |
| Balance training: NEXA | Some concerns | Low risk | No concerns | Some concerns | No concerns | Some concerns | Low |
| Balance training: Placebo | Some concerns | Low risk | No concerns | Some concerns | Some concerns | Some concerns | Low |
| Balance training: TENS | Some concerns | Low risk | No concerns | Some concerns | Some concerns | Some concerns | Low |
| General nursing: Laser | Some concerns | Low risk | No concerns | Some concerns | No concerns | Some concerns | Low |
| General nursing: NEXA | Some concerns | Low risk | No concerns | Some concerns | No concerns | Some concerns | Low |
| General nursing: Placebo | Some concerns | Low risk | No concerns | Some concerns | No concerns | Some concerns | Low |
| Laser: NEXA | Some concerns | Low risk | No concerns | Some concerns | No concerns | Some concerns | Low |
| Laser: Strength training | Some concerns | Low risk | No concerns | Some concerns | No concerns | Some concerns | Low |
| Laser: TENS | Some concerns | Low risk | No concerns | No concerns | Major concerns | Some concerns | Low |
| NEXA: Placebo | Some concerns | Low risk | No concerns | No concerns | Some concerns | Some concerns | Low |
| NEXA: TENS | Some concerns | Low risk | No concerns | Some concerns | No concerns | Some concerns | Low |
| Strength training: TENS | Some concerns | Low risk | No concerns | Some concerns | No concerns | Some concerns | Low |

`

**Table S4.4:** CINeMA results of WOMAC pain <1 month.

| **Comparison** | **Within-study bias** | **Reporting bias** | **Indirectness** | **Imprecision** | **Heterogeneity** | **Incoherence** | **Confidence rating** |
| --- | --- | --- | --- | --- | --- | --- | --- |
| Aerobic exercise: General nursing | Some concerns | Low risk | No concerns | Some concerns | Some concerns | No concerns | Low |
| Aerobic exercise: Placebo | No concerns | Low risk | No concerns | Some concerns | Some concerns | No concerns | Low |
| General nursing: Laser | No concerns | Low risk | No concerns | Major concerns | No concerns | No concerns | Low |
| General nursing: Strength training | No concerns | Low risk | No concerns | Major concerns | No concerns | No concerns | Low |
| Laser: Placebo | Some concerns | Low risk | No concerns | Some concerns | Some concerns | No concerns | Low |
| Placebo: Shockwave | Some concerns | Low risk | No concerns | No concerns | Some concerns | No concerns | Low |
| Placebo: TENS | Some concerns | Low risk | No concerns | No concerns | Major concerns | No concerns | Low |
| Aerobic exercise: Laser | No concerns | Low risk | No concerns | Major concerns | No concerns | No concerns | Low |
| Aerobic exercise: Shockwave | Some concerns | Low risk | No concerns | Major concerns | No concerns | No concerns | Low |
| Aerobic exercise: Strength training | No concerns | Low risk | No concerns | Major concerns | No concerns | No concerns | Low |
| Aerobic exercise: TENS | No concerns | Low risk | No concerns | Major concerns | No concerns | No concerns | Low |
| General nursing: Placebo | No concerns | Low risk | No concerns | Major concerns | No concerns | No concerns | Low |
| General nursing: Shockwave | Some concerns | Low risk | No concerns | Some concerns | Some concerns | No concerns | Low |
| General nursing: TENS | No concerns | Low risk | No concerns | Major concerns | No concerns | No concerns | Low |
| Laser: Shockwave | Some concerns | Low risk | No concerns | Some concerns | Some concerns | No concerns | Low |
| Laser: Strength training | No concerns | Low risk | No concerns | Major concerns | No concerns | No concerns | Low |
| Laser: TENS | Some concerns | Low risk | No concerns | Some concerns | Some concerns | No concerns | Low |
| Placebo: Strength training | No concerns | Low risk | No concerns | Major concerns | No concerns | No concerns | Low |
| Shockwave: Strength training | No concerns | Low risk | No concerns | Major concerns | No concerns | No concerns | Low |
| Shockwave: TENS | Some concerns | Low risk | No concerns | Major concerns | No concerns | No concerns | Low |
| Strength training: TENS | No concerns | Low risk | No concerns | Major concerns | No concerns | No concerns | Low |

**Table S4.5:** CINeMA results of WOMAC pain 1-3 months.

| **Comparison** | **Within-study bias** | **Reporting bias** | **Indirectness** | **Imprecision** | **Heterogeneity** | **Incoherence** | **Confidence rating** |
| --- | --- | --- | --- | --- | --- | --- | --- |
| Aerobic exercise: General nursing | Some concerns | Low risk | No concerns | Some concerns | Some concerns | No concerns | Low |
| Aerobic exercise: Placebo | Some concerns | Low risk | No concerns | No concerns | Major concerns | No concerns | Low |
| Aquatic sports: General nursing | Some concerns | Low risk | No concerns | No concerns | Some concerns | No concerns | Low |
| Balance training: General nursing | Some concerns | Low risk | No concerns | Some concerns | Some concerns | No concerns | Low |
| Balance training: Strength training | Some concerns | Low risk | No concerns | Major concerns | No concerns | No concerns | Low |
| General nursing: Laser | No concerns | Low risk | No concerns | Major concerns | No concerns | No concerns | Low |
| General nursing: Strength training | Some concerns | Low risk | No concerns | No concerns | Major concerns | No concerns | Low |
| Laser: Placebo | Some concerns | Low risk | No concerns | Major concerns | No concerns | No concerns | Low |
| Placebo: Shockwave | Some concerns | Low risk | No concerns | No concerns | Major concerns | No concerns | Low |
| Placebo: Strength training | Some concerns | Low risk | No concerns | No concerns | Some concerns | No concerns | Low |
| Placebo: TENS | Some concerns | Low risk | No concerns | No concerns | Some concerns | No concerns | Low |
| Shockwave: TENS | Some concerns | Low risk | No concerns | Some concerns | Some concerns | No concerns | Low |
| Aerobic exercise: Aquatic sports | Some concerns | Low risk | No concerns | Some concerns | Some concerns | No concerns | Low |
| Aerobic exercise: Balance training | Some concerns | Low risk | No concerns | Major concerns | No concerns | No concerns | Low |
| Aerobic exercise: Laser | Some concerns | Low risk | No concerns | Some concerns | Some concerns | No concerns | Low |
| Aerobic exercise: Shockwave | Some concerns | Low risk | No concerns | Major concerns | No concerns | No concerns | Low |
| Aerobic exercise: Strength training | Some concerns | Low risk | No concerns | Major concerns | No concerns | No concerns | Low |
| Aerobic exercise: TENS | Some concerns | Low risk | No concerns | Some concerns | Some concerns | No concerns | Low |
| Aquatic sports: Balance training | Some concerns | Low risk | No concerns | Some concerns | Some concerns | No concerns | Low |
| Aquatic sports: Laser | Some concerns | Low risk | No concerns | No concerns | Some concerns | No concerns | Low |
| Aquatic sports: Placebo | Some concerns | Low risk | No concerns | No concerns | Some concerns | No concerns | Low |
| Aquatic sports: Shockwave | Some concerns | Low risk | No concerns | Some concerns | Some concerns | No concerns | Low |
| Aquatic sports: Strength training | Some concerns | Low risk | No concerns | Some concerns | Some concerns | No concerns | Low |
| Aquatic sports: TENS | Some concerns | Low risk | No concerns | Major concerns | No concerns | No concerns | Low |
| Balance training: Laser | Some concerns | Low risk | No concerns | Major concerns | No concerns | No concerns | Low |
| Balance training: Placebo | Some concerns | Low risk | No concerns | Some concerns | Some concerns | No concerns | Low |
| Balance training: Shockwave | Some concerns | Low risk | No concerns | Major concerns | No concerns | No concerns | Low |
| Balance training: TENS | Some concerns | Low risk | No concerns | Some concerns | Some concerns | No concerns | Low |
| General nursing: Placebo | Some concerns | Low risk | No concerns | Some concerns | Some concerns | No concerns | Low |
| General nursing: Shockwave | Some concerns | Low risk | No concerns | Some concerns | Some concerns | No concerns | Low |
| General nursing: TENS | Some concerns | Low risk | No concerns | No concerns | Some concerns | No concerns | Low |
| Laser: Shockwave | Some concerns | Low risk | No concerns | Some concerns | Some concerns | No concerns | Low |
| Laser: Strength training | Some concerns | Low risk | No concerns | Some concerns | Some concerns | No concerns | Low |
| Laser: TENS | Some concerns | Low risk | No concerns | No concerns | Some concerns | No concerns | Low |
| Shockwave: Strength training | Some concerns | Low risk | No concerns | Some concerns | Some concerns | No concerns | Low |
| Strength training: TENS | Some concerns | Low risk | No concerns | Some concerns | Some concerns | No concerns | Low |

**Table S4.6:** CINeMA results of WOMAC pain >3 months.

| **Comparison** | **Within-study bias** | **Reporting bias** | **Indirectness** | **Imprecision** | **Heterogeneity** | **Incoherence** | **Confidence rating** |
| --- | --- | --- | --- | --- | --- | --- | --- |
| Aerobic exercise: General nursing | Some concerns | Low risk | No concerns | Some concerns | Some concerns | No concerns | Low |
| Aquatic training: General nursing | Major concerns | Low risk | No concerns | No concerns | No concerns | No concerns | Low |
| General nursing: Strength training | Some concerns | Low risk | No concerns | No concerns | Some concerns | No concerns | Low |
| Laser: Placebo | Major concerns | Low risk | No concerns | Some concerns | Some concerns | No concerns | Low |
| NEXA: Strength training | Some concerns | Low risk | No concerns | Some concerns | Some concerns | No concerns | Low |
| Placebo: Strength training | Some concerns | Low risk | No concerns | Some concerns | No concerns | No concerns | Low |
| Placebo: TENS | Some concerns | Low risk | No concerns | Some concerns | Some concerns | No concerns | Low |
| Aerobic exercise: Aquatic training | Some concerns | Low risk | No concerns | No concerns | Some concerns | No concerns | Low |
| Aerobic exercise: Laser | Some concerns | Low risk | No concerns | Major concerns | No concerns | No concerns | Low |
| Aerobic exercise: NEXA | Some concerns | Low risk | No concerns | Major concerns | No concerns | No concerns | Low |
| Aerobic exercise: Placebo | Some concerns | Low risk | No concerns | Major concerns | No concerns | No concerns | Low |
| Aerobic exercise: Strength training | Some concerns | Low risk | No concerns | Some concerns | Some concerns | No concerns | Low |
| Aerobic exercise: TENS | Some concerns | Low risk | No concerns | Major concerns | No concerns | No concerns | Low |
| Aquatic training: Laser | Some concerns | Low risk | No concerns | Some concerns | Some concerns | No concerns | Low |
| Aquatic training: NEXA | Some concerns | Low risk | No concerns | Some concerns | Some concerns | No concerns | Low |
| Aquatic training: Placebo | Some concerns | Low risk | No concerns | Some concerns | No concerns | No concerns | Low |
| Aquatic training: Strength training | Some concerns | Low risk | No concerns | Some concerns | Some concerns | No concerns | Low |
| Aquatic training: TENS | Some concerns | Low risk | No concerns | Some concerns | Some concerns | No concerns | Low |
| General nursing: Laser | Some concerns | Low risk | No concerns | Some concerns | Some concerns | No concerns | Low |
| General nursing: NEXA | Some concerns | Low risk | No concerns | Some concerns | Some concerns | No concerns | Low |
| General nursing: Placebo | Some concerns | Low risk | No concerns | Some concerns | Some concerns | No concerns | Low |
| General nursing: TENS | Some concerns | Low risk | No concerns | Some concerns | No concerns | No concerns | Low |
| Laser: NEXA | Some concerns | Low risk | No concerns | Major concerns | No concerns | No concerns | Low |
| Laser: Strength training | Some concerns | Low risk | No concerns | Some concerns | Some concerns | No concerns | Low |
| Laser: TENS | Some concerns | Low risk | No concerns | Some concerns | Some concerns | No concerns | Low |
| NEXA: Placebo | Some concerns | Low risk | No concerns | Major concerns | No concerns | No concerns | Low |
| NEXA: TENS | Some concerns | Low risk | No concerns | Major concerns | No concerns | No concerns | Low |
| Strength training: TENS | Some concerns | Low risk | No concerns | Some concerns | Some concerns | No concerns | Low |

**Table S4.7:** CINeMA results of WOMAC stiffness <1 month.

| **Comparison** | **Within-study bias** | **Reporting bias** | **Indirectness** | **Imprecision** | **Heterogeneity** | **Incoherence** | **Confidence rating** |
| --- | --- | --- | --- | --- | --- | --- | --- |
| Aerobic exercise: General nursing | Some concerns | Low risk | No concerns | Major concerns | No concerns | No concerns | Low |
| Aerobic exercise: Placebo | No concerns | Low risk | No concerns | Major concerns | No concerns | No concerns | Low |
| General nursing: Laser | No concerns | Low risk | No concerns | Major concerns | No concerns | No concerns | Low |
| General nursing: Strength training | No concerns | Low risk | No concerns | Major concerns | No concerns | No concerns | Low |
| Laser: Placebo | Some concerns | Low risk | No concerns | Major concerns | No concerns | No concerns | Low |
| Placebo: Shockwave | Some concerns | Low risk | No concerns | Some concerns | Some concerns | No concerns | Low |
| Placebo: TENS | Some concerns | Low risk | No concerns | Some concerns | Some concerns | No concerns | Low |
| Aerobic exercise: Laser | No concerns | Low risk | No concerns | Major concerns | No concerns | No concerns | Low |
| Aerobic exercise: Shockwave | Some concerns | Low risk | No concerns | Major concerns | No concerns | No concerns | Low |
| Aerobic exercise: Strength training | No concerns | Low risk | No concerns | Major concerns | No concerns | No concerns | Low |
| Aerobic exercise: TENS | Some concerns | Low risk | No concerns | Major concerns | No concerns | No concerns | Low |
| General nursing: Placebo | No concerns | Low risk | No concerns | Major concerns | No concerns | No concerns | Low |
| General nursing: Shockwave | Some concerns | Low risk | No concerns | Major concerns | No concerns | No concerns | Low |
| General nursing: TENS | Some concerns | Low risk | No concerns | Major concerns | No concerns | No concerns | Low |
| Laser: Shockwave | Some concerns | Low risk | No concerns | Major concerns | No concerns | No concerns | Low |
| Laser: Strength training | No concerns | Low risk | No concerns | Major concerns | No concerns | No concerns | Low |
| Laser: TENS | Some concerns | Low risk | No concerns | Major concerns | No concerns | No concerns | Low |
| Placebo: Strength training | No concerns | Low risk | No concerns | Major concerns | No concerns | No concerns | Low |
| Shockwave: Strength training | No concerns | Low risk | No concerns | Major concerns | No concerns | No concerns | Low |
| Shockwave: TENS | Some concerns | Low risk | No concerns | Major concerns | No concerns | No concerns | Low |
| Strength training: TENS | No concerns | Low risk | No concerns | Major concerns | No concerns | No concerns | Low |

**Table S4.8:** CINeMA results of WOMAC stiffness 1-3 months.

| **Comparison** | **Within-study bias** | **Reporting bias** | **Indirectness** | **Imprecision** | **Heterogeneity** | **Incoherence** | **Confidence rating** |
| --- | --- | --- | --- | --- | --- | --- | --- |
| Aerobic exercise: General nursing | Some concerns | Low risk | No concerns | No concerns | Major concerns | No concerns | Low |
| Aerobic exercise: Placebo | Some concerns | Low risk | No concerns | No concerns | Some concerns | No concerns | Low |
| Aquatic sports: General nursing | No concerns | Low risk | No concerns | Some concerns | Some concerns | No concerns | Low |
| Aquatic sports: Strength training | Some concerns | Low risk | No concerns | Major concerns | No concerns | No concerns | Low |
| Balance training: General nursing | Major concerns | Low risk | No concerns | Major concerns | No concerns | No concerns | Low |
| General nursing: Laser | No concerns | Low risk | No concerns | Major concerns | No concerns | No concerns | Low |
| General nursing: Strength training | Some concerns | Low risk | No concerns | Some concerns | Some concerns | No concerns | Low |
| Laser: Placebo | No concerns | Low risk | No concerns | Major concerns | No concerns | No concerns | Low |
| Placebo: Shockwave | No concerns | Low risk | No concerns | Some concerns | Some concerns | No concerns | Low |
| Placebo: Strength training | Some concerns | Low risk | No concerns | Some concerns | Some concerns | No concerns | Low |
| Placebo: TENS | Some concerns | Low risk | No concerns | No concerns | Some concerns | No concerns | Low |
| Shockwave: TENS | Some concerns | Low risk | No concerns | Some concerns | Some concerns | No concerns | Low |
| Aerobic exercise: Aquatic sports | Some concerns | Low risk | No concerns | Major concerns | No concerns | No concerns | Low |
| Aerobic exercise: Balance training | Some concerns | Low risk | No concerns | Major concerns | No concerns | No concerns | Low |
| Aerobic exercise: Laser | No concerns | Low risk | No concerns | Some concerns | Some concerns | No concerns | Low |
| Aerobic exercise: Shockwave | Some concerns | Low risk | No concerns | Some concerns | Some concerns | No concerns | Low |
| Aerobic exercise: Strength training | Some concerns | Low risk | No concerns | Major concerns | No concerns | No concerns | Low |
| Aerobic exercise: TENS | Some concerns | Low risk | No concerns | Major concerns | No concerns | No concerns | Low |
| Aquatic sports: Balance training | Some concerns | Low risk | No concerns | Major concerns | No concerns | No concerns | Low |
| Aquatic sports: Laser | No concerns | Low risk | No concerns | Major concerns | No concerns | No concerns | Low |
| Aquatic sports: Placebo | Some concerns | Low risk | No concerns | Some concerns | Some concerns | No concerns | Low |
| Aquatic sports: Shockwave | Some concerns | Low risk | No concerns | Major concerns | No concerns | No concerns | Low |
| Aquatic sports: TENS | Some concerns | Low risk | No concerns | Major concerns | No concerns | No concerns | Low |
| Balance training: Laser | Some concerns | Low risk | No concerns | Major concerns | No concerns | No concerns | Low |
| Balance training: Placebo | Some concerns | Low risk | No concerns | Major concerns | No concerns | No concerns | Low |
| Balance training: Shockwave | Some concerns | Low risk | No concerns | Major concerns | No concerns | No concerns | Low |
| Balance training: Strength training | Some concerns | Low risk | No concerns | Major concerns | No concerns | No concerns | Low |
| Balance training: TENS | Some concerns | Low risk | No concerns | Major concerns | No concerns | No concerns | Low |
| General nursing: Placebo | Some concerns | Low risk | No concerns | Major concerns | No concerns | No concerns | Low |
| General nursing: Shockwave | Some concerns | Low risk | No concerns | Major concerns | No concerns | No concerns | Low |
| General nursing: TENS | Some concerns | Low risk | No concerns | Some concerns | Some concerns | No concerns | Low |
| Laser: Shockwave | No concerns | Low risk | No concerns | Major concerns | No concerns | No concerns | Low |
| Laser: Strength training | No concerns | Low risk | No concerns | Major concerns | No concerns | No concerns | Low |
| Laser: TENS | Some concerns | Low risk | No concerns | Major concerns | No concerns | No concerns | Low |
| Shockwave: Strength training | Some concerns | Low risk | No concerns | Major concerns | No concerns | No concerns | Low |
| Strength training: TENS | Some concerns | Low risk | No concerns | Major concerns | No concerns | No concerns | Low |

**Table S4.9:** CINeMA results of WOMAC stiffness >3 months.

| **Comparison** | **Within-study bias** | **Reporting bias** | **Indirectness** | **Imprecision** | **Heterogeneity** | **Incoherence** | **Confidence rating** |
| --- | --- | --- | --- | --- | --- | --- | --- |
| Aerobic exercise: General nursing | Some concerns | Low risk | No concerns | Some concerns | Some concerns | No concerns | Low |
| Aerobic exercise: Placebo | Some concerns | Low risk | No concerns | No concerns | Major concerns | No concerns | Low |
| Aquatic sports: General nursing | Some concerns | Low risk | No concerns | No concerns | Some concerns | No concerns | Low |
| Balance training: General nursing | Some concerns | Low risk | No concerns | Some concerns | Some concerns | No concerns | Low |
| Balance training: Strength training | Some concerns | Low risk | No concerns | Major concerns | No concerns | No concerns | Low |
| General nursing: Laser | No concerns | Low risk | No concerns | Major concerns | No concerns | No concerns | Low |
| General nursing: Strength training | Some concerns | Low risk | No concerns | No concerns | Major concerns | No concerns | Low |
| Laser: Placebo | Some concerns | Low risk | No concerns | Major concerns | No concerns | No concerns | Low |
| Placebo: Shockwave | Some concerns | Low risk | No concerns | No concerns | Major concerns | No concerns | Low |
| Placebo: Strength training | Some concerns | Low risk | No concerns | No concerns | Some concerns | No concerns | Low |
| Placebo: TENS | Some concerns | Low risk | No concerns | No concerns | Some concerns | No concerns | Low |
| Shockwave: TENS | Some concerns | Low risk | No concerns | Some concerns | Some concerns | No concerns | Low |
| Aerobic exercise: Aquatic sports | Some concerns | Low risk | No concerns | Some concerns | Some concerns | No concerns | Low |
| Aerobic exercise: Balance training | Some concerns | Low risk | No concerns | Major concerns | No concerns | No concerns | Low |
| Aerobic exercise: Laser | Some concerns | Low risk | No concerns | Some concerns | Some concerns | No concerns | Low |
| Aerobic exercise: Shockwave | Some concerns | Low risk | No concerns | Major concerns | No concerns | No concerns | Low |
| Aerobic exercise: Strength training | Some concerns | Low risk | No concerns | Major concerns | No concerns | No concerns | Low |
| Aerobic exercise: TENS | Some concerns | Low risk | No concerns | Some concerns | Some concerns | No concerns | Low |
| Aquatic sports: Balance training | Some concerns | Low risk | No concerns | Some concerns | Some concerns | No concerns | Low |
| Aquatic sports: Laser | Some concerns | Low risk | No concerns | No concerns | Some concerns | No concerns | Low |
| Aquatic sports: Placebo | Some concerns | Low risk | No concerns | No concerns | Some concerns | No concerns | Low |

**Table S4.10:** CINeMA results of WOMAC function <1 month.

| **Comparison** | **Within-study bias** | **Reporting bias** | **Indirectness** | **Imprecision** | **Heterogeneity** | **Incoherence** | **Confidence rating** |
| --- | --- | --- | --- | --- | --- | --- | --- |
| Aerobic exercise: General nursing | Some concerns | Low risk | No concerns | Major concerns | No concerns | No concerns | Low |
| Aerobic exercise: Placebo | No concerns | Low risk | No concerns | Major concerns | No concerns | No concerns | Low |
| General nursing: Laser | No concerns | Low risk | No concerns | Major concerns | No concerns | No concerns | Low |
| General nursing: Strength training | No concerns | Low risk | No concerns | Major concerns | No concerns | No concerns | Low |
| Laser: Placebo | Some concerns | Low risk | No concerns | Major concerns | No concerns | No concerns | Low |
| Placebo: Shockwave | Major concerns | Low risk | No concerns | Some concerns | Some concerns | No concerns | Low |
| Placebo: TENS | Some concerns | Low risk | No concerns | Some concerns | Some concerns | No concerns | Low |
| Aerobic exercise: Laser | No concerns | Low risk | No concerns | Major concerns | No concerns | No concerns | Low |
| Aerobic exercise: Shockwave | Some concerns | Low risk | No concerns | Major concerns | No concerns | No concerns | Low |
| Aerobic exercise: Strength training | No concerns | Low risk | No concerns | Major concerns | No concerns | No concerns | Low |
| Aerobic exercise: TENS | Some concerns | Low risk | No concerns | Major concerns | No concerns | No concerns | Low |
| General nursing: Placebo | No concerns | Low risk | No concerns | Major concerns | No concerns | No concerns | Low |
| General nursing: Shockwave | Some concerns | Low risk | No concerns | Major concerns | No concerns | No concerns | Low |
| General nursing: TENS | Some concerns | Low risk | No concerns | Major concerns | No concerns | No concerns | Low |
| Laser: Shockwave | Some concerns | Low risk | No concerns | Major concerns | No concerns | No concerns | Low |
| Laser: Strength training | No concerns | Low risk | No concerns | Major concerns | No concerns | No concerns | Low |
| Laser: TENS | Some concerns | Low risk | No concerns | Major concerns | No concerns | No concerns | Low |
| Placebo: Strength training | No concerns | Low risk | No concerns | Major concerns | No concerns | No concerns | Low |
| Shockwave: Strength training | Some concerns | Low risk | No concerns | Major concerns | No concerns | No concerns | Low |
| Shockwave: TENS | Some concerns | Low risk | No concerns | Major concerns | No concerns | No concerns | Low |
| Strength training: TENS | No concerns | Low risk | No concerns | Major concerns | No concerns | No concerns | Low |

**Table S4.11:** CINeMA results of WOMAC function 1-3 months.

| **Comparison** | **Within-study bias** | **Reporting bias** | **Indirectness** | **Imprecision** | **Heterogeneity** | **Incoherence** | **Confidence rating** |
| --- | --- | --- | --- | --- | --- | --- | --- |
| Aerobic exercise: General nursing | Some concerns | Low risk | No concerns | Major concerns | No concerns | No concerns | Low |
| Aerobic exercise: Placebo | Some concerns | Low risk | No concerns | Some concerns | Some concerns | No concerns | Low |
| Aquatic sports: General nursing | Some concerns | Low risk | No concerns | No concerns | Major concerns | No concerns | Low |
| Balance training: General nursing | No concerns | Low risk | No concerns | Some concerns | Some concerns | No concerns | Low |
| Balance training: Placebo | Some concerns | Low risk | No concerns | Some concerns | Some concerns | No concerns | Low |
| Balance training: Strength training | Some concerns | Low risk | No concerns | Some concerns | Some concerns | No concerns | Low |
| General nursing: Laser | Some concerns | Low risk | No concerns | Major concerns | No concerns | No concerns | Low |
| General nursing: Strength training | Some concerns | Low risk | No concerns | Major concerns | No concerns | No concerns | Low |
| Laser: Placebo | Some concerns | Low risk | No concerns | No concerns | Major concerns | No concerns | Low |
| Placebo: Shockwave | Some concerns | Low risk | No concerns | Major concerns | No concerns | No concerns | Low |
| Placebo: Strength training | Some concerns | Low risk | No concerns | No concerns | Major concerns | No concerns | Low |
| Placebo: TENS | Some concerns | Low risk | No concerns | No concerns | Some concerns | No concerns | Low |
| Shockwave: TENS | Some concerns | Low risk | No concerns | No concerns | Some concerns | No concerns | Low |
| Aerobic exercise: Aquatic sports | Some concerns | Low risk | No concerns | Some concerns | Some concerns | No concerns | Low |
| Aerobic exercise: Balance training | Some concerns | Low risk | No concerns | Major concerns | No concerns | No concerns | Low |
| Aerobic exercise: Laser | Some concerns | Low risk | No concerns | Major concerns | No concerns | No concerns | Low |
| Aerobic exercise: Shockwave | Some concerns | Low risk | No concerns | Major concerns | No concerns | No concerns | Low |
| Aerobic exercise: Strength training | Some concerns | Low risk | No concerns | Major concerns | No concerns | No concerns | Low |
| Aerobic exercise: TENS | Some concerns | Low risk | No concerns | Major concerns | No concerns | No concerns | Low |
| Aquatic sports: Balance training | Some concerns | Low risk | No concerns | Major concerns | No concerns | No concerns | Low |
| Aquatic sports: Laser | Some concerns | Low risk | No concerns | Major concerns | No concerns | No concerns | Low |
| Aquatic sports: Placebo | Some concerns | Low risk | No concerns | Major concerns | No concerns | No concerns | Low |
| Aquatic sports: Shockwave | Some concerns | Low risk | No concerns | Major concerns | No concerns | No concerns | Low |
| Aquatic sports: Strength training | Some concerns | Low risk | No concerns | Major concerns | No concerns | No concerns | Low |
| Aquatic sports: TENS | Some concerns | Low risk | No concerns | Some concerns | Some concerns | No concerns | Low |
| Balance training: Laser | Some concerns | Low risk | No concerns | Major concerns | No concerns | No concerns | Low |
| Balance training: Shockwave | Some concerns | Low risk | No concerns | Major concerns | No concerns | No concerns | Low |
| Balance training: TENS | Some concerns | Low risk | No concerns | Major concerns | No concerns | No concerns | Low |
| General nursing: Placebo | Some concerns | Low risk | No concerns | Major concerns | No concerns | No concerns | Low |
| General nursing: Shockwave | Some concerns | Low risk | No concerns | Some concerns | Some concerns | No concerns | Low |
| General nursing: TENS | Some concerns | Low risk | No concerns | Some concerns | Some concerns | No concerns | Low |
| Laser: Shockwave | Some concerns | Low risk | No concerns | Major concerns | No concerns | No concerns | Low |
| Laser: Strength training | Some concerns | Low risk | No concerns | Major concerns | No concerns | No concerns | Low |
| Laser: TENS | Some concerns | Low risk | No concerns | Major concerns | No concerns | No concerns | Low |
| Shockwave: Strength training | Some concerns | Low risk | No concerns | Major concerns | No concerns | No concerns | Low |
| Strength training: TENS | Some concerns | Low risk | No concerns | Major concerns | No concerns | No concerns | Low |

**Table S4.12:** CINeMA results of WOMAC function >3 months.

| **Comparison** | **Within-study bias** | **Reporting bias** | **Indirectness** | **Imprecision** | **Heterogeneity** | **Incoherence** | **Confidence rating** |
| --- | --- | --- | --- | --- | --- | --- | --- |
| Aerobic exercise: General nursing | Some concerns | Low risk | No concerns | Some concerns | No concerns | No concerns | Low |
| General nursing: Strength training | No concerns | Low risk | No concerns | No concerns | Some concerns | No concerns | Moderate |
| Laser: Placebo | Major concerns | Low risk | No concerns | Some concerns | Some concerns | No concerns | Low |
| NEXA: Strength training | Some concerns | Low risk | No concerns | Major concerns | No concerns | No concerns | Low |
| Placebo: Strength training | Some concerns | Low risk | No concerns | Some concerns | Some concerns | No concerns | Low |
| Placebo: TENS | Some concerns | Low risk | No concerns | Some concerns | Some concerns | No concerns | Low |
| Aerobic exercise: Laser | Some concerns | Low risk | No concerns | Major concerns | No concerns | No concerns | Low |
| Aerobic exercise: NEXA | Some concerns | Low risk | No concerns | Major concerns | No concerns | No concerns | Low |
| Aerobic exercise: Placebo | Some concerns | Low risk | No concerns | Major concerns | No concerns | No concerns | Low |
| Aerobic exercise: Strength training | Some concerns | Low risk | No concerns | Some concerns | Some concerns | No concerns | Low |
| Aerobic exercise: TENS | Some concerns | Low risk | No concerns | Major concerns | No concerns | No concerns | Low |
| General nursing: Laser | Some concerns | Low risk | No concerns | Major concerns | No concerns | No concerns | Low |
| General nursing: NEXA | Some concerns | Low risk | No concerns | Some concerns | Some concerns | No concerns | Low |
| General nursing: Placebo | Some concerns | Low risk | No concerns | Some concerns | Some concerns | No concerns | Low |
| General nursing: TENS | Some concerns | Low risk | No concerns | Some concerns | Some concerns | No concerns | Low |
| Laser: NEXA | Some concerns | Low risk | No concerns | Major concerns | No concerns | No concerns | Low |
| Laser: Strength training | Some concerns | Low risk | No concerns | Some concerns | Some concerns | No concerns | Low |
| Laser: TENS | Some concerns | Low risk | No concerns | Major concerns | No concerns | No concerns | Low |
| NEXA: Placebo | Some concerns | Low risk | No concerns | Some concerns | Some concerns | No concerns | Low |
| NEXA: TENS | Some concerns | Low risk | No concerns | Major concerns | No concerns | No concerns | Low |
| Strength training: TENS | Some concerns | Low risk | No concerns | Some concerns | Some concerns | No concerns | Low |

**Table S4.13:** CINeMA results of WOMAC total score <1 month.

| **Comparison** | **Within-study bias** | **Reporting bias** | **Indirectness** | **Imprecision** | **Heterogeneity** | **Incoherence** | **Confidence rating** |
| --- | --- | --- | --- | --- | --- | --- | --- |
| Aerobic exercise: General nursing | Some concerns | Low risk | No concerns | Major concerns | No concerns | No concerns | Low |
| Aerobic exercise: Placebo | No concerns | Low risk | No concerns | Some concerns | Some concerns | No concerns | Low |
| Balance training: Strength training | Some concerns | Low risk | No concerns | Major concerns | No concerns | No concerns | Low |
| General nursing: Laser | No concerns | Low risk | No concerns | Major concerns | No concerns | No concerns | Low |
| General nursing: Strength training | No concerns | Low risk | No concerns | Major concerns | No concerns | No concerns | Low |
| Laser: Placebo | Some concerns | Low risk | No concerns | Some concerns | Some concerns | Some concerns | Low |
| Laser: Shockwave | No concerns | Low risk | No concerns | Some concerns | Some concerns | Some concerns | Low |
| Placebo: Shockwave | No concerns | Low risk | No concerns | No concerns | Major concerns | Some concerns | Low |
| Placebo: TENS | No concerns | Low risk | No concerns | Some concerns | Some concerns | No concerns | Low |
| Aerobic exercise: Balance training | Some concerns | Low risk | No concerns | Major concerns | No concerns | No concerns | Low |
| Aerobic exercise: Laser | No concerns | Low risk | No concerns | Major concerns | No concerns | No concerns | Low |
| Aerobic exercise: Shockwave | No concerns | Low risk | No concerns | Major concerns | No concerns | No concerns | Low |
| Aerobic exercise: Strength training | No concerns | Low risk | No concerns | Major concerns | No concerns | No concerns | Low |
| Aerobic exercise: TENS | No concerns | Low risk | No concerns | Major concerns | No concerns | No concerns | Low |
| Balance training: General nursing | Some concerns | Low risk | No concerns | Major concerns | No concerns | No concerns | Low |
| Balance training: Laser | No concerns | Low risk | No concerns | Major concerns | No concerns | No concerns | Low |
| Balance training: Placebo | No concerns | Low risk | No concerns | Major concerns | No concerns | No concerns | Low |
| Balance training: Shockwave | No concerns | Low risk | No concerns | Major concerns | No concerns | No concerns | Low |
| Balance training: TENS | No concerns | Low risk | No concerns | Major concerns | No concerns | No concerns | Low |
| General nursing: Placebo | No concerns | Low risk | No concerns | Major concerns | No concerns | No concerns | Low |
| General nursing: Shockwave | No concerns | Low risk | No concerns | Major concerns | No concerns | No concerns | Low |
| General nursing: TENS | No concerns | Low risk | No concerns | Major concerns | No concerns | No concerns | Low |
| Laser: Strength training | No concerns | Low risk | No concerns | Major concerns | No concerns | No concerns | Low |
| Laser: TENS | No concerns | Low risk | No concerns | Major concerns | No concerns | No concerns | Low |
| Placebo: Strength training | No concerns | Low risk | No concerns | Major concerns | No concerns | No concerns | Low |
| Shockwave: Strength training | No concerns | Low risk | No concerns | Major concerns | No concerns | No concerns | Low |
| Shockwave: TENS | No concerns | Low risk | No concerns | Major concerns | No concerns | No concerns | Low |
| Strength training: TENS | No concerns | Low risk | No concerns | Major concerns | No concerns | No concerns | Low |

**Table S4.14:** CINeMA results of WOMAC total score 1-3 months.

| **Comparison** | **Within-study bias** | **Reporting bias** | **Indirectness** | **Imprecision** | **Heterogeneity** | **Incoherence** | **Confidence rating** |
| --- | --- | --- | --- | --- | --- | --- | --- |
| Aerobic exercise: General nursing | Some concerns | Low risk | No concerns | No concerns | Major concerns | No concerns | Low |
| Aerobic exercise: Placebo | Some concerns | Low risk | No concerns | No concerns | Some concerns | No concerns | Low |
| Aquatic sports: General nursing | Some concerns | Low risk | No concerns | No concerns | Major concerns | No concerns | Low |
| Balance training: General nursing | Some concerns | Low risk | No concerns | Some concerns | Some concerns | No concerns | Low |
| Balance training: Strength training | Some concerns | Low risk | No concerns | Major concerns | No concerns | No concerns | Low |
| General nursing: Laser | No concerns | Low risk | No concerns | Major concerns | No concerns | No concerns | Low |
| General nursing: Strength training | Some concerns | Low risk | No concerns | No concerns | Major concerns | No concerns | Low |
| Laser: Placebo | No concerns | Low risk | No concerns | Some concerns | Some concerns | No concerns | Low |
| Placebo: Shockwave | No concerns | Low risk | No concerns | No concerns | Some concerns | No concerns | Moderate |
| Placebo: Strength training | Some concerns | Low risk | No concerns | No concerns | Some concerns | No concerns | Low |
| Placebo: TENS | Some concerns | Low risk | No concerns | No concerns | Major concerns | No concerns | Low |
| Shockwave: TENS | Some concerns | Low risk | No concerns | Major concerns | No concerns | No concerns | Low |
| Aerobic exercise: Aquatic sports | Some concerns | Low risk | No concerns | Major concerns | No concerns | No concerns | Low |
| Aerobic exercise: Balance training | Some concerns | Low risk | No concerns | Major concerns | No concerns | No concerns | Low |
| Aerobic exercise: Laser | No concerns | Low risk | No concerns | Some concerns | Some concerns | No concerns | Low |
| Aerobic exercise: Shockwave | Some concerns | Low risk | No concerns | Major concerns | No concerns | No concerns | Low |
| Aerobic exercise: Strength training | Some concerns | Low risk | No concerns | Major concerns | No concerns | No concerns | Low |
| Aerobic exercise: TENS | Some concerns | Low risk | No concerns | Major concerns | No concerns | No concerns | Low |
| Aquatic sports: Balance training | Some concerns | Low risk | No concerns | Major concerns | No concerns | No concerns | Low |
| Aquatic sports: Laser | Some concerns | Low risk | No concerns | Major concerns | No concerns | No concerns | Low |
| Aquatic sports: Placebo | Some concerns | Low risk | No concerns | No concerns | Some concerns | No concerns | Low |
| Aquatic sports: Shockwave | Some concerns | Low risk | No concerns | Major concerns | No concerns | No concerns | Low |
| Aquatic sports: Strength training | Some concerns | Low risk | No concerns | Major concerns | No concerns | No concerns | Low |
| Aquatic sports: TENS | Some concerns | Low risk | No concerns | Major concerns | No concerns | No concerns | Low |
| Balance training: Laser | Some concerns | Low risk | No concerns | Major concerns | No concerns | No concerns | Low |
| Balance training: Placebo | Some concerns | Low risk | No concerns | Some concerns | Some concerns | No concerns | Low |
| Balance training: Shockwave | Some concerns | Low risk | No concerns | Major concerns | No concerns | No concerns | Low |
| Balance training: TENS | Some concerns | Low risk | No concerns | Major concerns | No concerns | No concerns | Low |
| General nursing: Placebo | Some concerns | Low risk | No concerns | Some concerns | Some concerns | No concerns | Low |
| General nursing: Shockwave | Some concerns | Low risk | No concerns | Some concerns | Some concerns | No concerns | Low |
| General nursing: TENS | Some concerns | Low risk | No concerns | Major concerns | No concerns | No concerns | Low |
| Laser: Shockwave | No concerns | Low risk | No concerns | Some concerns | Some concerns | No concerns | Low |
| Laser: Strength training | No concerns | Low risk | No concerns | Some concerns | Some concerns | No concerns | Low |
| Laser: TENS | No concerns | Low risk | No concerns | Major concerns | No concerns | No concerns | Low |
| Shockwave: Strength training | Some concerns | Low risk | No concerns | Major concerns | No concerns | No concerns | Low |
| Strength training: TENS | Some concerns | Low risk | No concerns | Major concerns | No concerns | No concerns | Low |

**Table S4.15:** CINeMA results of WOMAC total score >3 months.

| **Comparison** | **Within-study bias** | **Reporting bias** | **Indirectness** | **Imprecision** | **Heterogeneity** | **Incoherence** | **Confidence rating** |
| --- | --- | --- | --- | --- | --- | --- | --- |
| Aerobic exercise: General nursing | Some concerns | Low risk | No concerns | Some concerns | Some concerns | No concerns | Low |
| Aquatic sports: General nursing | Major concerns | Low risk | No concerns | Some concerns | No concerns | No concerns | Low |
| Balance training: Strength training | Some concerns | Low risk | No concerns | Major concerns | No concerns | No concerns | Low |
| General nursing: Strength training | Some concerns | Low risk | No concerns | No concerns | Some concerns | No concerns | Low |
| Laser: Placebo | Some concerns | Low risk | No concerns | Major concerns | No concerns | No concerns | Low |
| NEXA: Strength training | Some concerns | Low risk | No concerns | Major concerns | No concerns | No concerns | Low |
| Placebo: Shockwave | Some concerns | Low risk | No concerns | No concerns | No concerns | No concerns | Moderate |
| Placebo: Strength training | Some concerns | Low risk | No concerns | No concerns | Some concerns | No concerns | Moderate |
| Placebo: TENS | No concerns | Low risk | No concerns | Some concerns | Some concerns | No concerns | Low |
| Aerobic exercise: Aquatic sports | Some concerns | Low risk | No concerns | Major concerns | No concerns | No concerns | Low |
| Aerobic exercise: Balance training | Some concerns | Low risk | No concerns | Major concerns | No concerns | No concerns | Low |
| Aerobic exercise: Laser | Some concerns | Low risk | No concerns | Major concerns | No concerns | No concerns | Low |
| Aerobic exercise: NEXA | Some concerns | Low risk | No concerns | Major concerns | No concerns | No concerns | Low |
| Aerobic exercise: Placebo | Some concerns | Low risk | No concerns | Major concerns | No concerns | No concerns | Low |
| Aerobic exercise: Shockwave | Some concerns | Low risk | No concerns | No concerns | No concerns | No concerns | Moderate |
| Aerobic exercise: Strength training | Some concerns | Low risk | No concerns | Some concerns | Some concerns | No concerns | Low |
| Aerobic exercise: TENS | Some concerns | Low risk | No concerns | Major concerns | No concerns | No concerns | Low |
| Aquatic sports: Balance training | Some concerns | Low risk | No concerns | Major concerns | No concerns | No concerns | Low |
| Aquatic sports: Laser | Some concerns | Low risk | No concerns | Major concerns | No concerns | No concerns | Low |
| Aquatic sports: NEXA | Some concerns | Low risk | No concerns | Major concerns | No concerns | No concerns | Low |
| Aquatic sports: Placebo | Some concerns | Low risk | No concerns | Major concerns | No concerns | No concerns | Low |
| Aquatic sports: Shockwave | Some concerns | Low risk | No concerns | No concerns | No concerns | No concerns | Moderate |
| Aquatic sports: Strength training | Some concerns | Low risk | No concerns | Some concerns | Some concerns | No concerns | Low |
| Aquatic sports: TENS | Some concerns | Low risk | No concerns | Major concerns | No concerns | No concerns | Low |
| Balance training: General nursing | Some concerns | Low risk | No concerns | Some concerns | Some concerns | No concerns | Low |
| Balance training: Laser | Some concerns | Low risk | No concerns | Major concerns | No concerns | No concerns | Low |
| Balance training: NEXA | Some concerns | Low risk | No concerns | Major concerns | No concerns | No concerns | Low |
| Balance training: Placebo | Some concerns | Low risk | No concerns | Major concerns | No concerns | No concerns | Low |
| Balance training: Shockwave | Some concerns | Low risk | No concerns | No concerns | No concerns | No concerns | Moderate |
| Balance training: TENS | Some concerns | Low risk | No concerns | Major concerns | No concerns | No concerns | Low |
| General nursing: Laser | Some concerns | Low risk | No concerns | Major concerns | No concerns | No concerns | Low |
| General nursing: NEXA | Some concerns | Low risk | No concerns | Some concerns | Some concerns | No concerns | Low |
| General nursing: Placebo | Some concerns | Low risk | No concerns | Some concerns | Some concerns | No concerns | Low |
| General nursing: Shockwave | Some concerns | Low risk | No concerns | No concerns | No concerns | No concerns | Moderate |
| General nursing: TENS | Some concerns | Low risk | No concerns | Some concerns | Some concerns | No concerns | Low |
| Laser: NEXA | Some concerns | Low risk | No concerns | Major concerns | No concerns | No concerns | Low |
| Laser: Shockwave | Some concerns | Low risk | No concerns | No concerns | No concerns | No concerns | Moderate |
| Laser: Strength training | Some concerns | Low risk | No concerns | Some concerns | Some concerns | No concerns | Low |
| Laser: TENS | Some concerns | Low risk | No concerns | Major concerns | No concerns | No concerns | Low |
| NEXA: Placebo | Some concerns | Low risk | No concerns | Some concerns | Some concerns | No concerns | Low |
| NEXA: Shockwave | Some concerns | Low risk | No concerns | No concerns | No concerns | No concerns | Moderate |
| NEXA: TENS | Some concerns | Low risk | No concerns | Major concerns | No concerns | No concerns | Low |
| Shockwave: Strength training | Some concerns | Low risk | No concerns | No concerns | No concerns | No concerns | Moderate |
| Shockwave: TENS | Some concerns | Low risk | No concerns | No concerns | No concerns | No concerns | Moderate |
| Strength training: TENS | Some concerns | Low risk | No concerns | Some concerns | Some concerns | No concerns | Low |

**Appendix 5: Evaluation of inconsistency and heterogeneity**

**Table S5.1:** Global consistency.

| **Clinical outcome** | **Chi square** | **P value** |
| --- | --- | --- |
| VAS pain during walking at less than 1 month | 1.64 | 0.6505 |
| VAS pain during walking at 1-3 months | 2.45 | 0.4839 |
| VAS pain during walking at more than 3 months | 6.59 | 0.0863 |
| WOMAC pain <1month | 0.28 | 0.5956 |
| WOMAC pain 1-3 months | 1.38 | 0.8475 |
| WOMAC stiffness <1month | 1.50 | 0.2206 |
| WOMAC stiffness 1-3 months | 1.20 | 0.8785 |
| WOMAC function <1month | 0.09 | 0.7653 |
| WOMAC function 1-3 months | 2.04 | 0.9226 |
| WOMAC total score <1month | 5.17 | 0.0753 |
| WOMAC total score 1-3 months | 3.29 | 0.5101 |
| WOMAC total score >3 months | 0.05 | 0.8166 |

**Table S5.2:** Side-splitting of VAS pain during walking at less than 1 month. Inconsistency test between direct and indirect treatment comparisons in mixed treatment comparison.

| Comparison | Direct | | Indirect | | Difference | |  | tau |
| --- | --- | --- | --- | --- | --- | --- | --- | --- |
|  | Coef. | Std. Err. | Coef. | Std. Err. | Coef. | Std. Err. | P>\|z\| |  |
| Placebo vs TENS | . | . | . | . | . | . | . | . |
| Placebo VS Shockwave | -1.589329 | .6957495 | -2.344353 | 1.500774 | .7550235 | 1.654341 | 0.648 | 1.62 |
| Placebo VS Laser | -2.627452 | .8397003 | -2.11105 | 1.45401 | -.5164018 | 1.679306 | 0.758 | 1.62 |
| Placebo VS Aerobic exercise | -2.68 | 1.831919 | -1.930341 | 2.21988 | -.7496593 | 2.878158 | 0.795 | 1.62 |
| Shockwave VS Laser | -1.54 | 1.672561 | -.5111377 | .9835893 | -1.028862 | 1.940337 | 0.596 | 1.62 |
| Shockwave VS General nursing | 2.36 | 1.560479 | -.4925274 | 1.496416 | 2.852528 | 2.162026 | 0.187 | 1.53 |
| Laser VS General nursing | .44 | 1.566309 | 2.843484 | 1.555276 | -2.403484 | 2.207308 | 0.276 | 1.56 |
| General nursing VS Aerobic exercise | -1.23 | 1.813505 | -1.979647 | 2.234953 | .7496473 | 2.878162 | 0.795 | 1.62 |

Abbreviations: TENS, Transcutaneous Electrical Nerve Stimulation;

**Table S5.3:** Side-splitting of VAS pain during walking at 1-3 months. Inconsistency test between direct and indirect treatment comparisons in mixed treatment comparison.

| Comparison | Direct | | Indirect | | Difference | |  | tau |
| --- | --- | --- | --- | --- | --- | --- | --- | --- |
|  | Coef. | Std. Err. | Coef. | Std. Err. | Coef. | Std. Err. | P>\|z\| |  |
| Placebo vs Aquatic sports | -1.36 | 2.049351 | -2.111071 | 1.700164 | .751071 | 2.66278 | 0.778 | 1.93 |
| Placebo vs Shockwave | -1.989212 | .9417142 | 2.163349 | 2.382627 | -4.152561 | 2.561939 | 0.105 | 1.79 |
| Placebo vs Strength training | -2.313328 | 1.153975 | -1.880902 | 2.650933 | -.4324263 | 2.891093 | 0.881 | 1.93 |
| Placebo vs Laser | -2.559623 | 1.166368 | -2.120309 | 2.59874 | -.4393137 | 2.847219 | 0.877 | 1.94 |
| Placebo vs TENS | -1.13665 | .9938139 | -5.289198 | 2.36137 | 4.152548 | 2.561938 | 0.105 | 1.79 |
| Aquatic sports vs Strength training | -.1999998 | 2.15136 | -.6324126 | 1.931351 | .4324128 | 2.891101 | 0.881 | 1.93 |
| Aquatic sports vs General nursing | 1.734578 | 1.533513 | 1.295735 | 2.402203 | .4388433 | 2.847482 | 0.878 | 1.94 |
| Shockwave vs TENS | -3.3 | 2.165469 | .8525628 | 1.369046 | -4.152563 | 2.561941 | 0.105 | 1.79 |
| Laser vs General nursing | 2.183839 | 1.390409 | 2.62303 | 2.48533 | -.439191 | 2.847405 | 0.877 | 1.94 |
| General nursing vs Aerobic exercise | -1.997401 | 1.441998 | .6057778 | 997.8931 | -2.603179 | 997.8941 | 0.998 | 1.86 |

Abbreviations: TENS, Transcutaneous Electrical Nerve Stimulation;

**Table S5.4:** Side-splitting of VAS pain during walking at more than 3 months. Inconsistency test between direct and indirect treatment comparisons in mixed treatment comparison.

| Comparison | Direct | | Indirect | | Difference | |  | tau |
| --- | --- | --- | --- | --- | --- | --- | --- | --- |
|  | Coef. | Std. Err. | Coef. | Std. Err. | Coef. | Std. Err. | P>\|z\| |  |
| Placebo vs Strength training | -1.6 | .5292807 | -.5632006 | .8432961 | -1.036799 | .9956337 | 0.298 | 0.33 |
| Placebo vs Aquatic sports | -1.67 | .8145977 | -.8038057 | .910019 | -.8661943 | 1.221353 | 0.478 | 0.45 |
| Placebo vs TENS | -.0229919 | .2268821 | -2.720305 | 1.064896 | 2.697313 | 1.088797 | 0.013 | 8.77e-08 |
| Placebo vs Laser | . | . | . | . | . | . | . | . |
| NEXA vs Strength training | .46 | .5602143 | -2.58316 | 1421.021 | 3.04316 | 1421.021 | 0.998 | 0.25 |
| Strength training vs Aerobic exercise | .499234 | .5026623 | 1.114544 | 1.311306 | -.6153098 | 1.404303 | 0.661 | 0.37 |
| Strength training vs General nursing | .100611 | .4609784 | 1.212444 | .7953317 | -1.111833 | .9215782 | 0.228 | 0.29 |
| Strength training vs Balance training | .8999999 | .6475618 | 2.511494 | 1459.303 | -1.611494 | 1459.303 | 0.999 | 0.25 |
| Aquatic sports vs General nursing | .2999999 | .4822337 | 1.166179 | 1.122114 | -.866179 | 1.221348 | 0.478 | 0.45 |
| TENS vs General nursing | 1.3 | .9747576 | -1.397314 | .4851058 | 2.697314 | 1.088797 | 0.013 | 7.79e-06 |
| Aerobic exercise vs General nursing | -.2677138 | .4477808 | 1.805872 | 1.963886 | -2.073586 | 1.991284 | 0.298 | 0.33 |

Abbreviations: NEXA, Neuromuscular exercise; TENS, Transcutaneous Electrical Nerve Stimulation.

**Table S5.5:** Side-splitting of WOMAC pain <1 month. Inconsistency test between direct and indirect treatment comparisons in mixed treatment comparison.

| Comparison | Direct | | Indirect | | Difference | |  | tau |
| --- | --- | --- | --- | --- | --- | --- | --- | --- |
|  | Coef. | Std. Err. | Coef. | Std. Err. | Coef. | Std. Err. | P>\|z\| |  |
| Placebo vs Strength training | .89 | 2.791589 | 3.309031 | 3.604698 | -2.419031 | 4.559256 | 0.596 | 2.17 |
| Placebo vs Shockwave | 3.19 | 2.48243 | .7703 | 3.823913 | 2.4197 | 4.559032 | 0.596 | 2.17 |
| Strength training vs Laser | -.7700001 | 2.179847 | 1.649523 | 4.00463 | -2.419523 | 4.559473 | 0.596 | 2.17 |
| Strength training vs Aerobic exercise | 6.22e-0.9 | 2.474419 | -3.623127 | 1730.824 | 3.623127 | 1730.826 | 0.998 | 2.06 |
| Laser vs Shockwave | .6503102 | 1.442926 | 3.069962 | 4.324961 | -2.419652 | 4.559312 | 0.596 | 2.17 |
| Shockwave vs TENS | -3.138761 | 1.31275 | -4.768707 | 1149.697 | 1.629946 | 1149.698 | 0.999 | 2.06 |
| Shockwave vs General nursing | -2.213144 | 1.220076 | -4.894793 | 710.9944 | 2.681648 | 710.9954 | 0.997 | 2.06 |

Abbreviations: TENS, Transcutaneous Electrical Nerve Stimulation.

**Table S5.6:** Side-splitting of WOMAC pain 1-3 months. Inconsistency test between direct and indirect treatment comparisons in mixed treatment comparison.

| Comparison | Direct | | Indirect | | Difference | |  | tau |
| --- | --- | --- | --- | --- | --- | --- | --- | --- |
|  | Coef. | Std. Err. | Coef. | Std. Err. | Coef. | Std. Err. | P>\|z\| |  |
| Placebo vs Strength training | -3.357473 | 1.064479 | -1.212813 | 1.766883 | -2.14466 | 2.063044 | 0.299 | 1.70 |
| Placebo vs Shockwave | -1.933188 | .7726057 | -.5282463 | 2.515086 | -1.404942 | 2.631017 | 0.593 | 1.74 |
| Placebo vs Laser | .1607867 | 1.45361 | -1.789984 | 2.106851 | 1.950771 | 2.559704 | 0.446 | 1.74 |
| Placebo vs Aerobic exercise | -2.132129 | 1.340333 | -3.061132 | 1.767934 | .9290029 | 2.218563 | 0.675 | 1.75 |
| Placebo vs TENS | -4.028253 | 1.101262 | -5.433178 | 2.389518 | 1.404925 | 2.631017 | 0.593 | 1.75 |
| General nursing vs Strength training | -1.441877 | 1.190371 | -3.309087 | 1.479066 | 1.86721 | 1.899101 | 0.326 | 1.71 |
| General nursing vs Balance training | -1.4 | 2.053713 | -1.260226 | 2.174931 | -.1397742 | 2.991331 | 0.963 | 1.76 |
| General nursing vs Laser | -.77 | 1.751616 | 1.180827 | 1.866559 | -1.950827 | 2.559727 | 0.446 | 1.74 |
| General nursing vs Aquatic sports | -3.964475 | 1.157064 | 1.856703 | 578.6188 | -5.821178 | 578.6199 | 0.992 | 1.72 |
| General nursing vs Aerobic exercise | -2.136292 | 1.213311 | -1.207259 | 1.857744 | -.9290326 | 2.218571 | 0.675 | 1.75 |
| Strength training vs Balance training | .8999999 | 1.927862 | .7602429 | 2.287226 | .139757 | 2.99133 | 0.963 | 1.76 |
| Shockwave vs TENS | -3.499999 | 2.26117 | -2.095064 | 1.345129 | -1.404936 | 2.631019 | 0.593 | 1.75 |

Abbreviations: TENS, Transcutaneous Electrical Nerve Stimulation.

**Table S5.7:** Side-splitting of WOMAC pain >3 months. Inconsistency test between direct and indirect treatment comparisons in mixed treatment comparison.

| Comparison | Direct | | Indirect | | Difference | |  | tau |
| --- | --- | --- | --- | --- | --- | --- | --- | --- |
|  | Coef. | Std. Err. | Coef. | Std. Err. | Coef. | Std. Err. | P>\|z\| |  |
| Placebo vs General nursing | -1.151384 | .7652324 | -.7219448 | 217.9748 | -.4294392 | 217.9762 | 0.998 | 1.09 |
| Placebo vs Laser | . | . | . | . | . | . | . | . |
| Placebo vs TENS | . | . | . | . | . | . | . | . |
| Strength training vs General nursing | -2.27882 | .7357579 | -2.761947 | 246.3796 | .4831265 | 246.3808 | 0.998 | 1.09 |
| Strength training vs Aerobic exercise | -.8561131 | .9438365 | -2.27678 | 887.348 | 1.420667 | 887.3486 | 0.999 | 1.09 |
| Strength training vs Aquatic sports | -3.9 | 1.134188 | -1.697728 | 622.8822 | -2.202272 | 622.8833 | 0.997 | 1.09 |
| General nursing vs NEXA | .7 | 1.267877 | 2.364934 | 1855.97 | -1.664934 | -1.664934 | 0.999 | 1.09 |

Abbreviations: NEXA, Neuromuscular exercise; TENS, Transcutaneous Electrical Nerve Stimulation.

**Table S5.8:** Side-splitting of WOMAC stiffness <1 month. Inconsistency test between direct and indirect treatment comparisons in mixed treatment comparison.

| Comparison | Direct | | Indirect | | Difference | |  | tau |
| --- | --- | --- | --- | --- | --- | --- | --- | --- |
|  | Coef. | Std. Err. | Coef. | Std. Err. | Coef. | Std. Err. | P>\|z\| |  |
| Placebo vs Laser | -.1049922 | .5368971 | -2.0399 | 1.485452 | 1.934908 | 1.579502 | 0.221 | 0.70 |
| Placebo vs Shockwave | . | . | . | . | . | . | . | . |
| Placebo vs Aerobic exercise | -1.29 | .9661857 | .6450031 | 1.24956 | -1.935003 | 1.57953 | 0.221 | 0.70 |
| Placebo vs TENS | . | . | . | . | . | . | . | . |
| General nursing vs Strength training | -1.01 | .491845 | .491845 | 729.2569 | -1.501845 | 729.2572 | 0.998 | 0.73 |
| General nursing vs Laser | -.5 | 1.434961 | 1.403168 | 1.403168 | -1.934961 | 1.579524 | 0.221 | 0.70 |
| General nursing vs Aerobic exercise | .2500001 | -1.685007 | -1.685007 | 1.322036 | 1.935007 | 1.579553 | 0.221 | 0.70 |

Abbreviations: TENS, Transcutaneous Electrical Nerve Stimulation.

**Table S5.9:** Side-splitting of WOMAC stiffness 1-3 months. Inconsistency test between direct and indirect treatment comparisons in mixed treatment comparison.

| Comparison | Direct | | Indirect | | Difference | |  | tau |
| --- | --- | --- | --- | --- | --- | --- | --- | --- |
|  | Coef. | Std. Err. | Coef. | Std. Err. | Coef. | Std. Err. | P>\|z\| |  |
| Placebo vs Balance training | 1.211354 | .5979729 | 2.407293 | 1.051737 | -1.209894 | 1.209894 | 0.288 | 0.85 |
| Placebo vs Laser | 2.138774 | .6123147 | .852648 | 1.043489 | 1.286126 | 1.20988 | 0.288 | 0.85 |
| General nursing vs Balance training | 2.199999 | 1.418761 | 1.22258 | 1.340896 | .977419 | 1.952148 | 0.617 | 0.88 |
| General nursing vs Aerobic exercise | .4 | 1.241291 | 1.377422 | 1.506679 | -.9774225 | 1.952149 | 0.617 | 0.88 |
| Strength training vs Balance training | -.7600001 | 1.011831 | 2.844635 | 904.1643 | -2.084635 | 904.1648 | 0.998 | 0.86 |
| Balance training vs Shockwave | -.6700001 | .8977343 | .4617331 | 1.247965 | -1.131733 | 1.537317 | 0.462 | 0.88 |
| Balance training vs Aerobic exercise | -.9224195 | .5617719 | -.7752236 | 1.046799 | -.1471959 | 1.187867 | 0.901 | 0.89 |
| Shockwave vs Laser | .1400001 | 1.039819 | 1.271766 | 1.132307 | -1.131766 | 1.537317 | 0.462 | 0.88 |
| Laser vs Aquatic sports | -.6202782 | .3876627 | -.5232769 | 1.371652 | -.0970013 | 1.425496 | 0.946 | 0.89 |
| Laser vs Aerobic exercise | -.8999999 | 1.005936 | -1.56437 | .9168567 | .6643698 | 1.361078 | 0.625 | 0.89 |
| Laser vs TENS | -1.623206 | .6589859 | -1.720297 | 1.263913 | .097091 | 1.425505 | 0.946 | 0.89 |
| Aquatic sports vs TWNS | -1.1 | 1.203 | -1.002926 | .76477 | -.97074 | 1.425511 | 0.946 | 0.89 |

Abbreviations: TENS, Transcutaneous Electrical Nerve Stimulation.

**Table S5.10:** Side-splitting of WOMAC stiffness >3 months. Inconsistency test between direct and indirect treatment comparisons in mixed treatment comparison.

| Comparison | Direct | | Indirect | | Difference | |  | tau |
| --- | --- | --- | --- | --- | --- | --- | --- | --- |
|  | Coef. | Std. Err. | Coef. | Std. Err. | Coef. | Std. Err. | P>\|z\| |  |
| Placebo vs General nursing | .228431 | .3006845 | .7071423 | 193.9803 | -.4787122 | 193.9806 | 0.998 | 1.70e-06 |
| General nursing vs Aerobic exercise | -.9298231 | .5451707 | -.4086464 | 211.1861 | -.5211767 | 211.1869 | 0.998 | 5.89e-07 |
| NEXA vs Laser | -.02 | .7810409 | -.4669821 | 1566.371 | .4469821 | 1566.371 | 1.000 | 4.38e-08 |
| Strength training vs Aerobic exercise | .1999994 | .3556114 | -1.41279 | 1049.237 | 1.61279 | 1049.238 | 0.999 | .00 |
| Laser vs Aerobic exercise | -.4679382 | .2892783 | -1.027724 | 332.5804 | .5597861 | 332.5806 | 0.999 | 3.92e-06 |
| Laser vs TENS | -.3 | .1659495 | .8342514 | 942.7168 | -1.134251 | 942.7168 | 0.999 | 4.59e-06 |

Abbreviations: NEXA, Neuromuscular exercise; D, Strength training;TENS, Transcutaneous Electrical Nerve Stimulation.

**Table S5.11:** Side-splitting of WOMAC function <1 month. Inconsistency test between direct and indirect treatment comparisons in mixed treatment comparison.

| Comparison | Direct | | Indirect | | Difference | |  | tau |
| --- | --- | --- | --- | --- | --- | --- | --- | --- |
|  | Coef. | Std. Err. | Coef. | Std. Err. | Coef. | Std. Err. | P>\|z\| |  |
| Placebo vs Laser | -.7373772 | 4.505813 | -4.978748 | 13.47572 | 4.241371 | 14.20906 | 0.765 | 7.01 |
| Placebo vs Shockwave | . | . | . | . | . | . | . | . |
| Placebo vs Aerobic exercise | -6.85 | 7.989037 | -2.607441 | 11.75282 | -4.242559 | 14.21103 | 0.765 | 7.01 |
| Placebo vs TENS | . | . | . | . | . | . | . | . |
| General nursing vs Strength training | .3800002 | 7.873297 | -.8720823 | 5647.853 | 1.252083 | 5647.857 | 1.000 | 6.36 |
| General nursing vs Laser | -2.609999 | 7.035152 | 1.632378 | 12.34732 | -4.242377 | 14.2109 | 0.765 | 7.01 |
| General nursing vs Aerobic exercise | -4.48 | 8.266663 | -8.722555 | 11.55927 | 4.242556 | 14.21106 | 0.765 | 7.01 |

Abbreviations: TENS, Transcutaneous Electrical Nerve Stimulation.

**Table S5.12:** Side-splitting of WOMAC function 1-3 months. Inconsistency test between direct and indirect treatment comparisons in mixed treatment comparison.

| Comparison | Direct | | Indirect | | Difference | |  | tau |
| --- | --- | --- | --- | --- | --- | --- | --- | --- |
|  | Coef. | Std. Err. | Coef. | Std. Err. | Coef. | Std. Err. | P>\|z\| |  |
| Placebo vs Strength training | -10.78796 | 2.922136 | -6.751611 | 5.762148 | -4.036352 | 6.461489 | 0.532 | 6.06 |
| Placebo vs Balance training | -8.263736 | 4.758904 | -5.230513 | 7.037555 | -3.033223 | 8.511992 | 0.722 | 6.09 |
| Placebo vs Shockwave | -6.545271 | 2.684953 | -8.632629 | 8.088671 | 2.087358 | 8.522636 | 0.807 | 6.10 |
| Placebo vs Laser | -1.010975 | 4.880976 | -5.228004 | 7.073791 | 4.207029 | 8.59447 | 0.624 | 6.09 |
| Placebo vs Aerobic exercise | -8.527017 | 4.238234 | -9.312785 | 6.099175 | .7857679 | 7.425282 | 0.916 | 6.12 |
| Placebo vs TENS | -12.63268 | 3.778923 | -10.54524 | 7.639072 | -2.087445 | 8.522642 | 0.807 | 6.10 |
| General nursing vs Strength training | -6.346339 | 3.820798 | -11.75007 | 4.953864 | 5.403735 | 6.255432 | 0.388 | 6.06 |
| General nursing vs Balance training | -8.960001 | 7.107409 | -3.845983 | 5.363961 | -5.114018 | 8.904343 | 0.566 | 6.07 |
| General nursing vs Laser | -2.900001 | 6.113779 | 1.30729 | 6.040583 | -4.20729 | 8.594587 | 0.624 | 6.09 |
| General nursing vs Aquatic sports | -9.987318 | 5.210248 | 3.827456 | 4990.562 | -13.81477 | 4900.565 | 0.998 | 5.96 |
| General nursing vs Aerobic exercise | -7.501563 | 4.784663 | -6.71556 | 5.676831 | -.7860032 | 7.425354 | 0.916 | 6.12 |
| Strength training vs Balance training | 8.714701 | 6.272437 | -1.518258 | 5.230803 | 10.23296 | 8.163463 | 0.210 | 5.88 |
| Shockwave vs TENS | -3.999999 | 7.151683 | -6.087406 | 4.635622 | 2.087407 | 8.52265 | 0.807 | 6.10 |

Abbreviations: TENS, Transcutaneous Electrical Nerve Stimulation.

**Table S5.13:** Side-splitting of WOMAC function >3 months. Inconsistency test between direct and indirect treatment comparisons in mixed treatment comparison.

| Comparison | Direct | | Indirect | | Difference | |  | tau |
| --- | --- | --- | --- | --- | --- | --- | --- | --- |
|  | Coef. | Std. Err. | Coef. | Std. Err. | Coef. | Std. Err. | P>\|z\| |  |
| Placebo vs Strength training | -4.214809 | 2.51464 | -3.790515 | 551.0052 | -.4242944 | 551.0111 | 0.999 | 3.48 |
| Placebo vs Laser | . | . | . | . | . | . | . | . |
| Placebo vs TENS | . | . | . | . | . | . | . | . |
| General nursing vs Strength training | -7.111361 | 2.267083 | -7.311091 | 745.8266 | .1997294 | 745.83 | 1.000 | 3.48 |
| General nursing vs Aerobic exercise | -5.348999 | 2.869497 | -5.709214 | 1736.352 | .3602152 | 1736.354 | 1.000 | 3.48 |
| Strength training vs NEXA | .400001 | 4.080935 | 8.342465 | 6129.022 | -7.942464 | 6129.024 | 0.999 | 3.48 |

Abbreviations: NEXA, Neuromuscular movement; TENS, Transcutaneous Electrical Nerve Stimulation.

**Table S5.14:** Side-splitting of WOMAC total score <1 month. Inconsistency test between direct and indirect treatment comparisons in mixed treatment comparison.

| Comparison | Direct | | Indirect | | Difference | |  | tau |
| --- | --- | --- | --- | --- | --- | --- | --- | --- |
|  | Coef. | Std. Err. | Coef. | Std. Err. | Coef. | Std. Err. | P>\|z\| |  |
| Placebo vs Shockwave | -12.60141 | 3.383979 | 6.752787 | 8.253613 | -19.3542 | 8.915172 | 0.030 | 6.00 |
| Placebo vs Laser | -1.510175 | 4.175088 | -19.23635 | 6.751794 | 17.72618 | 7.931726 | 0.025 | 5.96 |
| Placebo vs TENS | . | . | . | . | . | . | . | . |
| Placebo vs Aerobic exercise | -11.28 | 8.618064 | -4.713531 | 13.23584 | -6.566469 | 15.79426 | 0.678 | 7.60 |
| General nursing vs Strength training | -2.130001 | 9.377922 | 3.447569 | 3908.163 | -5.57757 | 3908.173 | 0.999 | 7.24 |
| General nursing vs Laser | -3.859999 | 7.648932 | 2.705588 | 13.81834 | -6.565587 | 15.79407 | 0.678 | 7.60 |
| General nursing vs Aerobic exercise | -2.830001 | 9.854253 | -9.39642 | 12.34319 | 6.566419 | 15.79432 | 0.678 | 7.60 |
| Strength training vs Balance training | 4.41 | 8.392652 | 12.09853 | 9127.707 | -7.688527 | 9127.71 | 0.999 | 7.24 |
| Shockwave vs Laser | -9.300001 | 7.228216 | 10.05421 | 5.218563 | -19.35421 | 8.915184 | 0.030 | 6.00 |

Abbreviations: TENS, Transcutaneous Electrical Nerve Stimulation.

**Table S5.15:** Side-splitting of WOMAC total score 1-3 months. Inconsistency test between direct and indirect treatment comparisons in mixed treatment comparison.

| Comparison | Direct | | Indirect | | Difference | |  | tau |
| --- | --- | --- | --- | --- | --- | --- | --- | --- |
|  | Coef. | Std. Err. | Coef. | Std. Err. | Coef. | Std. Err. | P>\|z\| |  |
| Placebo vs Strength training | -13.1 | 8.005871 | -14.06854 | 6.756908 | .9685374 | 10.47615 | 0.926 | 7.05 |
| Placebo VS Shockwave | -16.84965 | 2.771418 | 2.47241 | 9.56451 | -19.32206 | 9.959716 | 0.052 | 6.17 |
| Placebo vs Laser | -7.140688 | 6.14636 | -8.34695 | 8.977633 | 1.206261 | 10.87931 | 0.912 | 7.07 |
| Placebo VS Aerobic exercise | -14.26346 | 4.890222 | -12.55915 | 8.075612 | -1.704312 | 9.440944 | 0.857 | 7.04 |
| Placebo vs TENS | -5.427595 | 5.151097 | -24.7496 | 8.522146 | 19.322 | 9.959725 | 0.052 | 6.17 |
| General nursing vs Strength training | -9.629525 | 4.066332 | -11.02747 | 7.437048 | 1.397949 | 8.475737 | 0.869 | 7.04 |
| General nursing VS Balance training; | -11.12 | 8.019344 | -6.961641 | 9.039741 | -4.158362 | 12.08415 | 0.731 | 7.01 |
| General nursing vs Laser | -4.33 | 7.124962 | -3.123433 | 8.221718 | -1.206568 | 10.87942 | 0.912 | 7.07 |
| General nursing vs Aquatic sports | -12.79995 | 5.625804 | 8.869641 | 2179.392 | -21.66959 | 2179.399 | 0.992 | 6.80 |
| General nursing vs Aerobic exercise | -9.639114 | 4.866759 | -11.34307 | 8.09026 | 1.703954 | 9.441108 | 0.857 | 7.04 |
| Strength training vs Balance training; | 2.589999 | 8.228475 | -1.568261 | 8.849806 | 4.15826 | 12.08416 | 0.731 | 7.01 |
| Shockwave vs TENS | -7.899996 | 8.058921 | 11.42207 | 5.852334 | -19.32206 | 9.959721 | 0.052 | 6.17 |

Abbreviations: TENS, Transcutaneous Electrical Nerve Stimulation.

**Table S5.16:** Side-splitting of WOMAC total score >3 months. Inconsistency test between direct and indirect treatment comparisons in mixed treatment comparison.

| Comparison | Direct | | Indirect | | Difference | |  | tau |
| --- | --- | --- | --- | --- | --- | --- | --- | --- |
|  | Coef. | Std. Err. | Coef. | Std. Err. | Coef. | Std. Err. | P>\|z\| |  |
| Placebo vs Strength training | -5.9524 | 3.578713 | -8.484189 | 734.1088 | 2.531789 | 734.1157 | 0.997 | 4.92 |
| Placebo vs Shockwave | . | . | . | . | . | . | . | . |
| Placebo vs TENS | . | . | . | . | . | . | . | . |
| Placebo vs Laser | . | . | . | . | . | . | . | . |
| General nursing vs Strength training | -12.63363 | 6.416826 | -9.447437 | 771.8661 | -3.186195 | 771.8859 | 0.997 | 4.92 |
| General nursing vs Aerobic exercise | -5.922289 | 3.79555 | -13.2721 | 2099.282 | 7.34981 | 2099.287 | 0.997 | 4.92 |
| General nursing vs Aquatic sports | -7.1 | 5.03061 | -12.34794 | 2297.353 | 5.247937 | 2297.358 | 0.998 | 4.92 |
| Strength training vs NEXA | .8999996 | 5.408305 | 11.96942 | 6465.608 | -11.06942 | 6465.611 | 0.999 | 4.92 |
| Strength training vs Balance training | 3.1 | 6.608542 | 11.73501 | 10550.27 | -8.63501 | 10550.27 | 0.999 | 4.92 |

Abbreviations: NEXA, Neuromuscular exercise; TENS, Transcutaneous Electrical Nerve Stimulation.

**Appendix 6: Network maps**

The size of the nodes was proportional to the number of participants included in the trial, and the thickness of lines between the interventions relates to the number of studies for that comparison.

Abbreviations: NEXA, Neuromuscular exercise; TENS, Transcutaneous Electrical Nerve Stimulation.

**Figure S6.1:** Network map of VAS pain at rest.


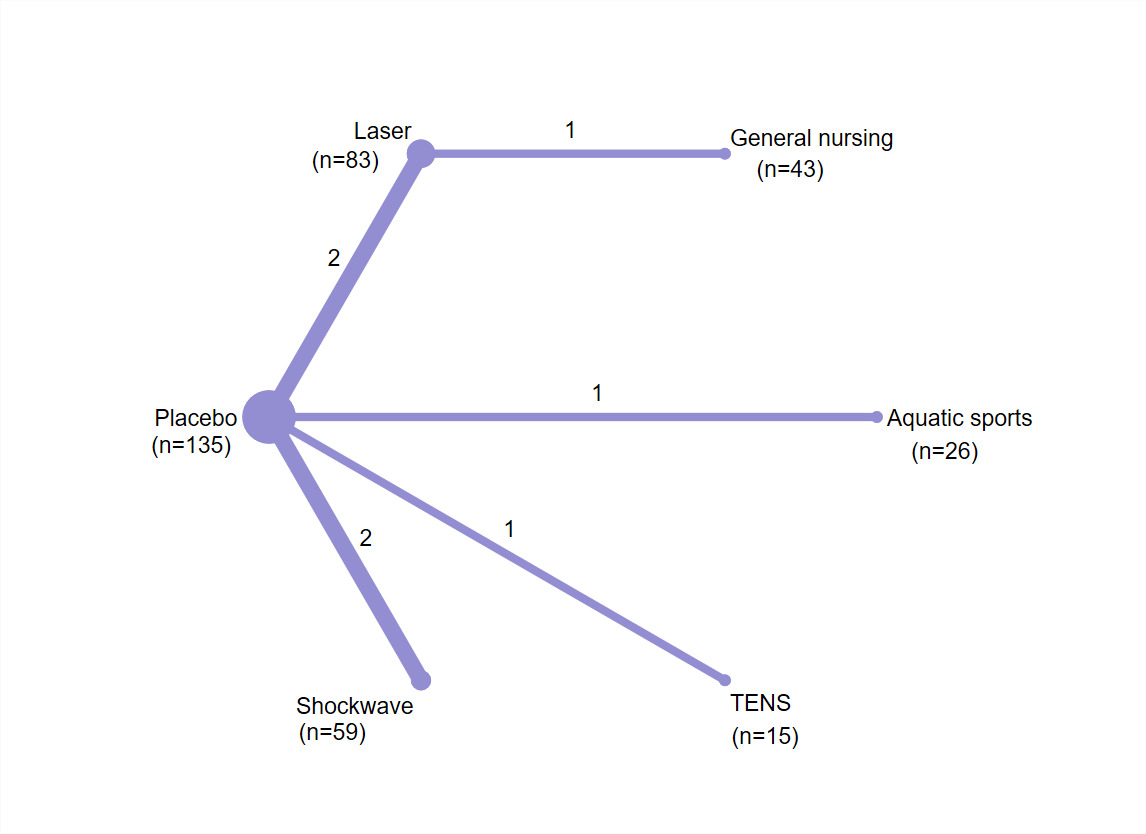


**Figure S6.2:** Network map of NRS.


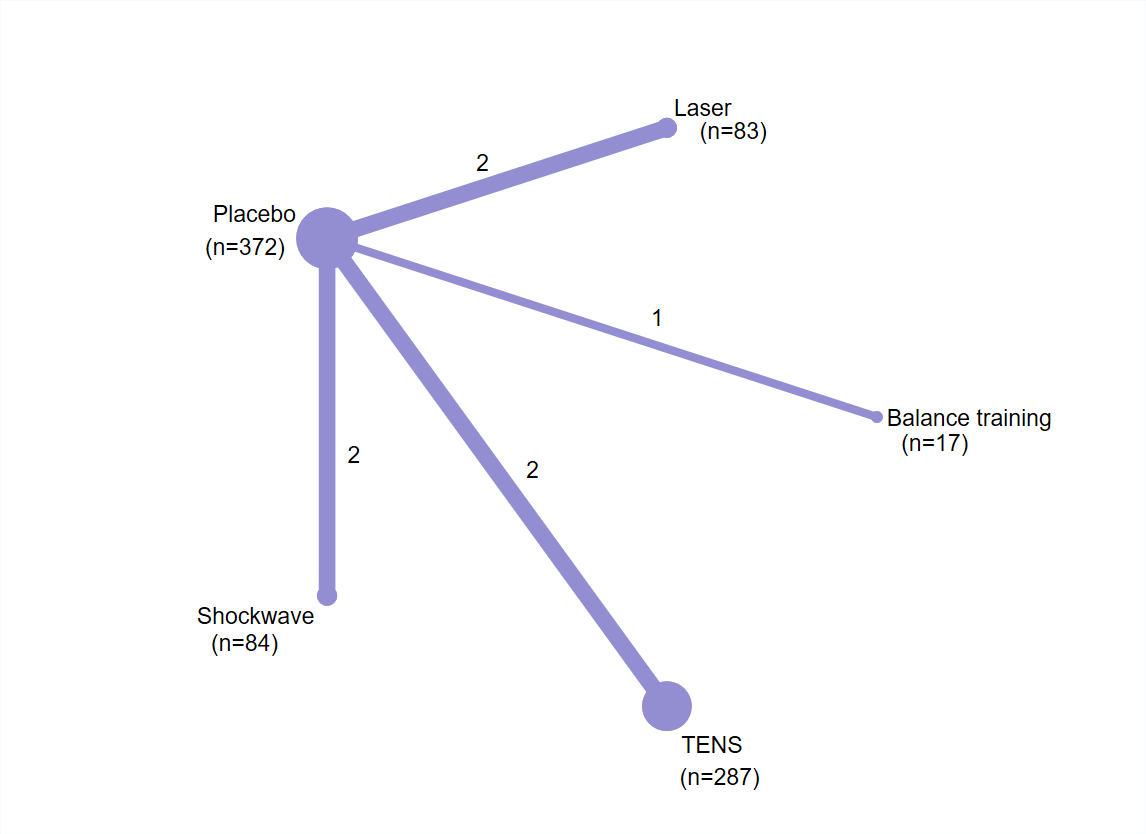


**Figure S6.3:** Network map of VAS pain during walking at less than 1 month.


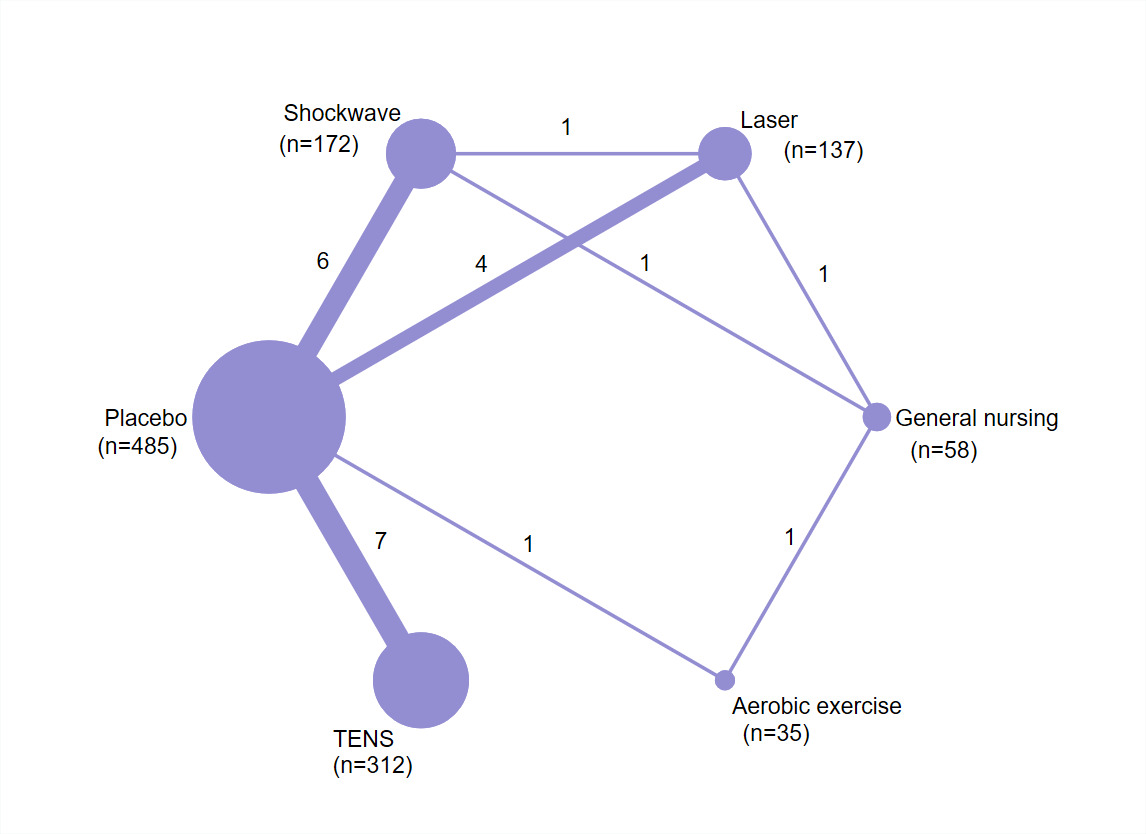


**Figure S6.4:** Network map of VAS pain during walking at 1-3 months.


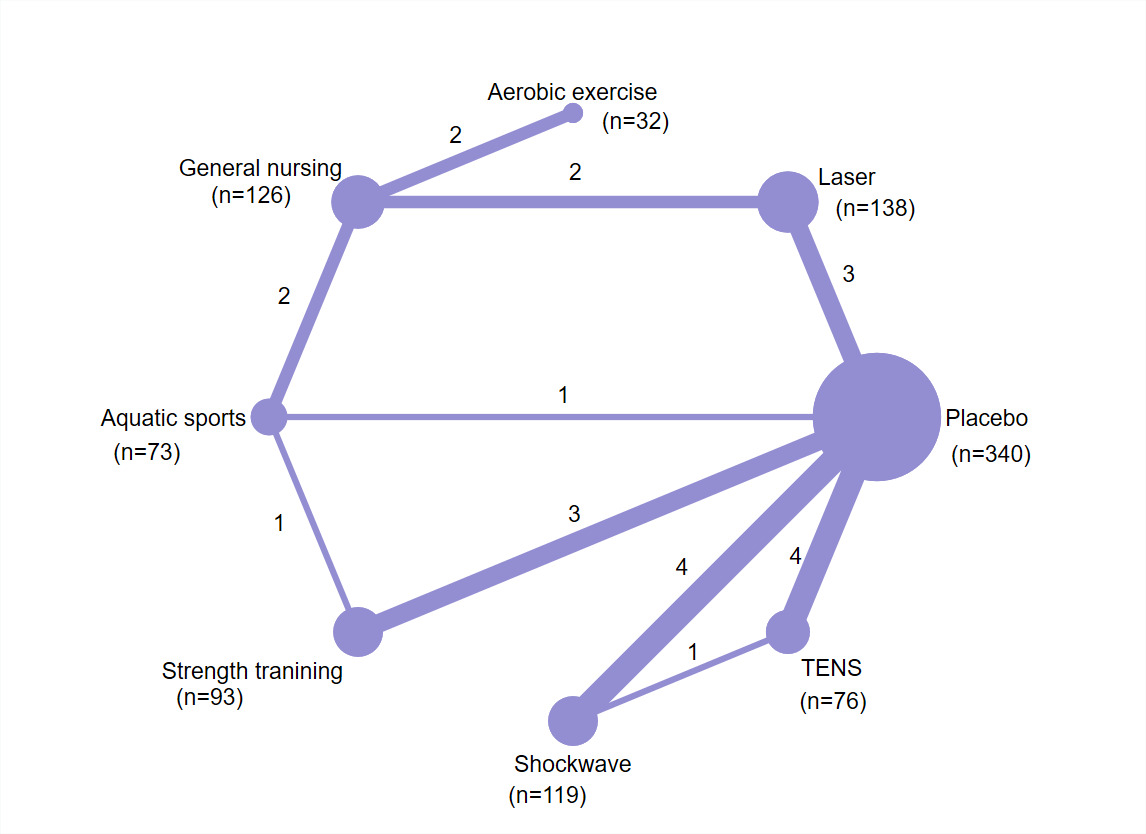


**Figure S6.5:** Network map of VAS pain during walking at more than 3 months.


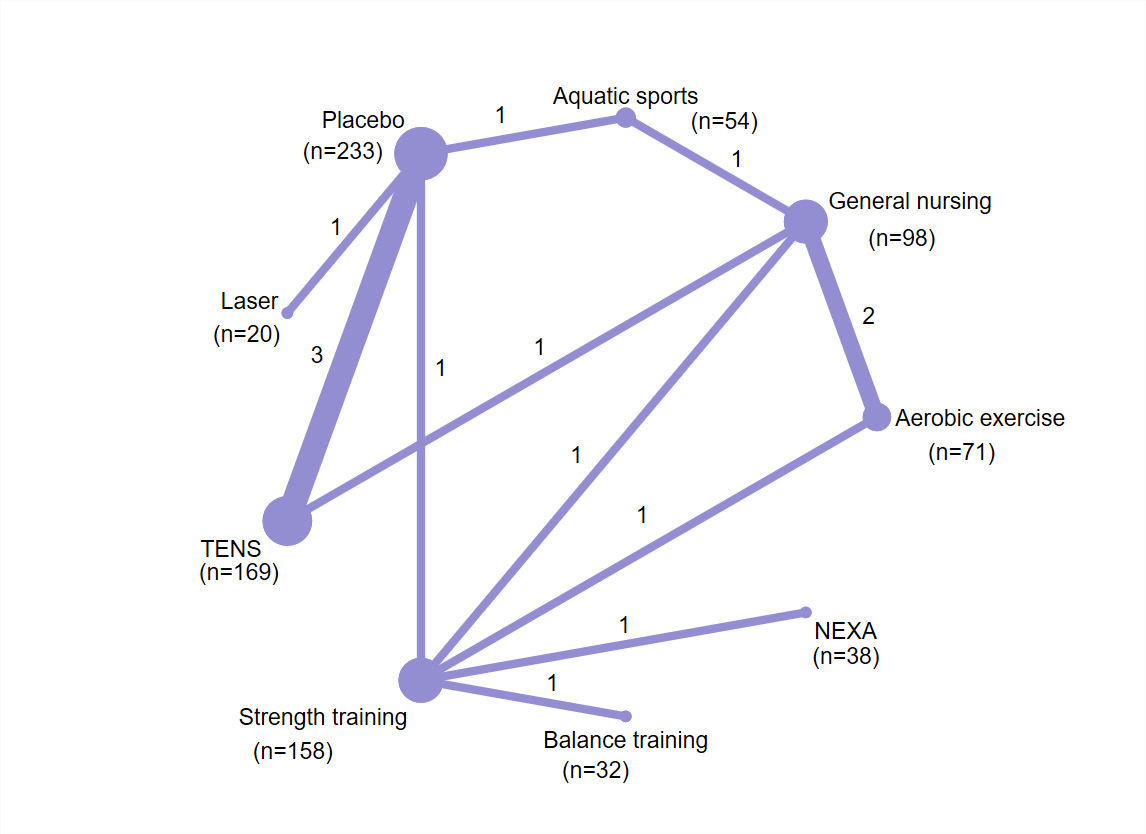


**Figure S6.6:** Network map of WOMAC pain at < 1 month.


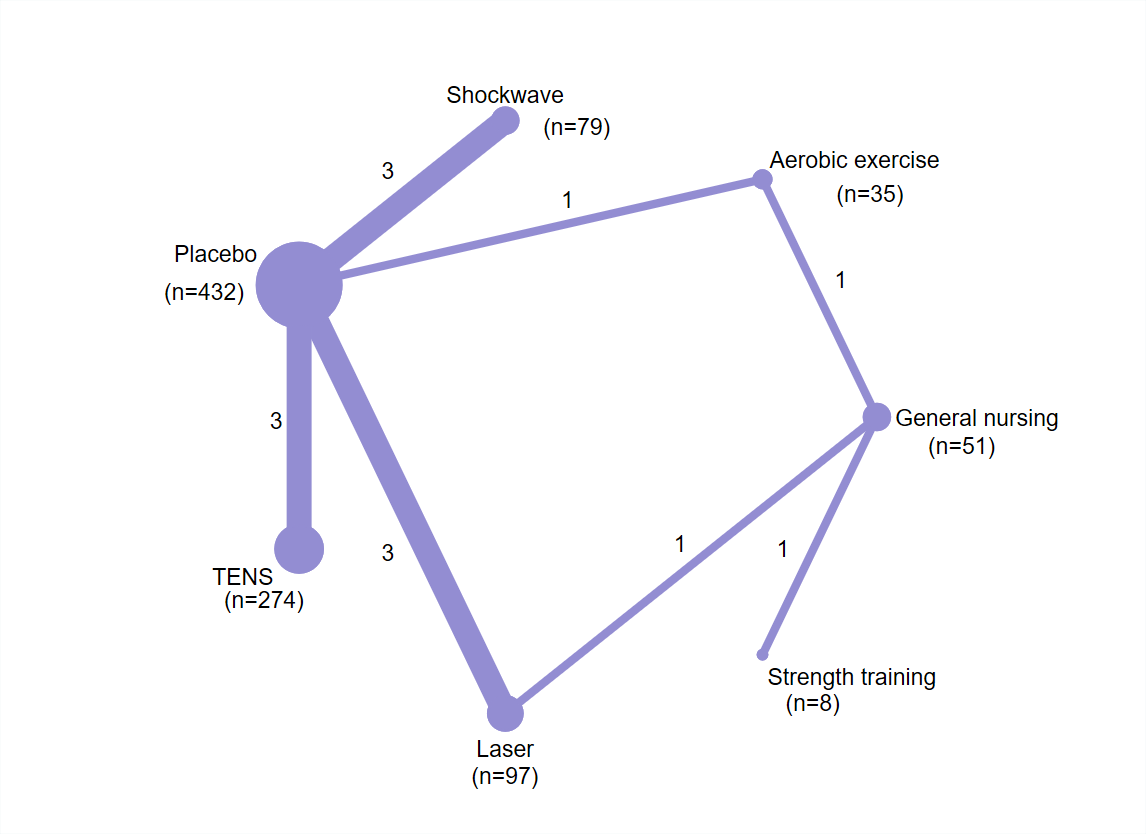


**Figure S6.7:** Network map of WOMAC pain at 1-3 months.


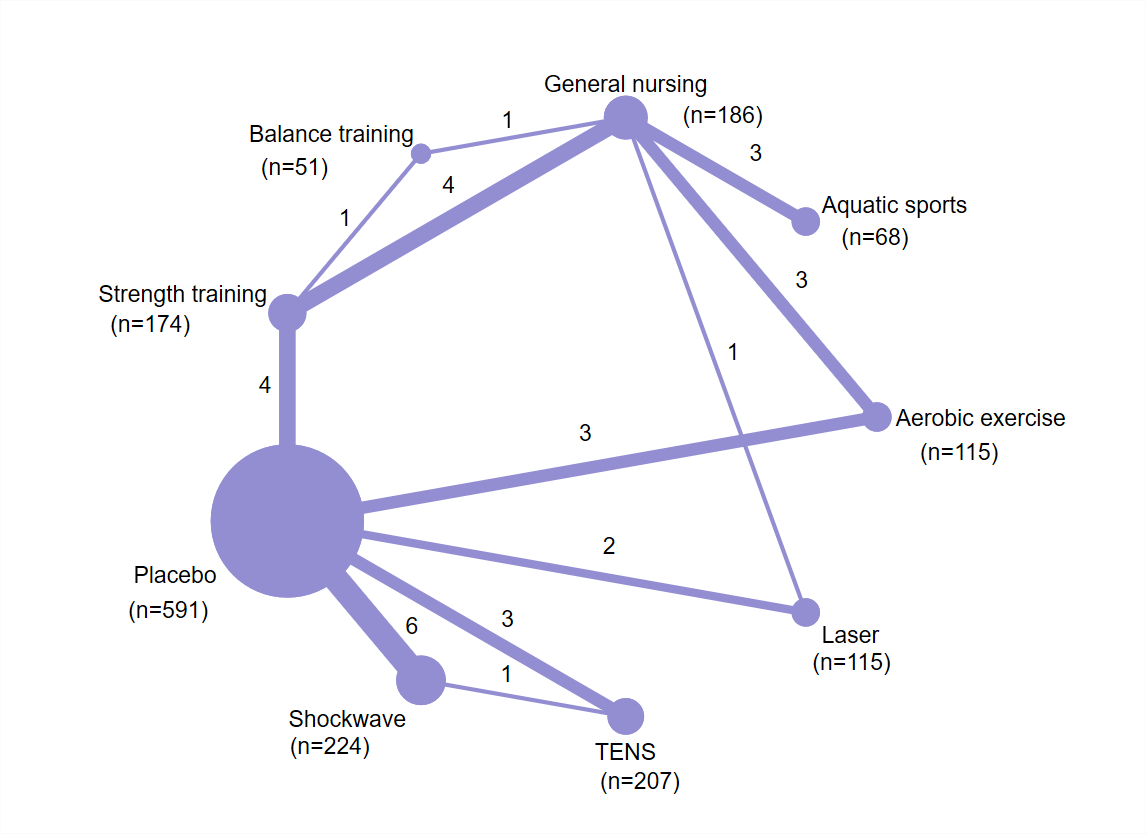


**Figure S6.8:** Network map of WOMAC pain at >3 months.


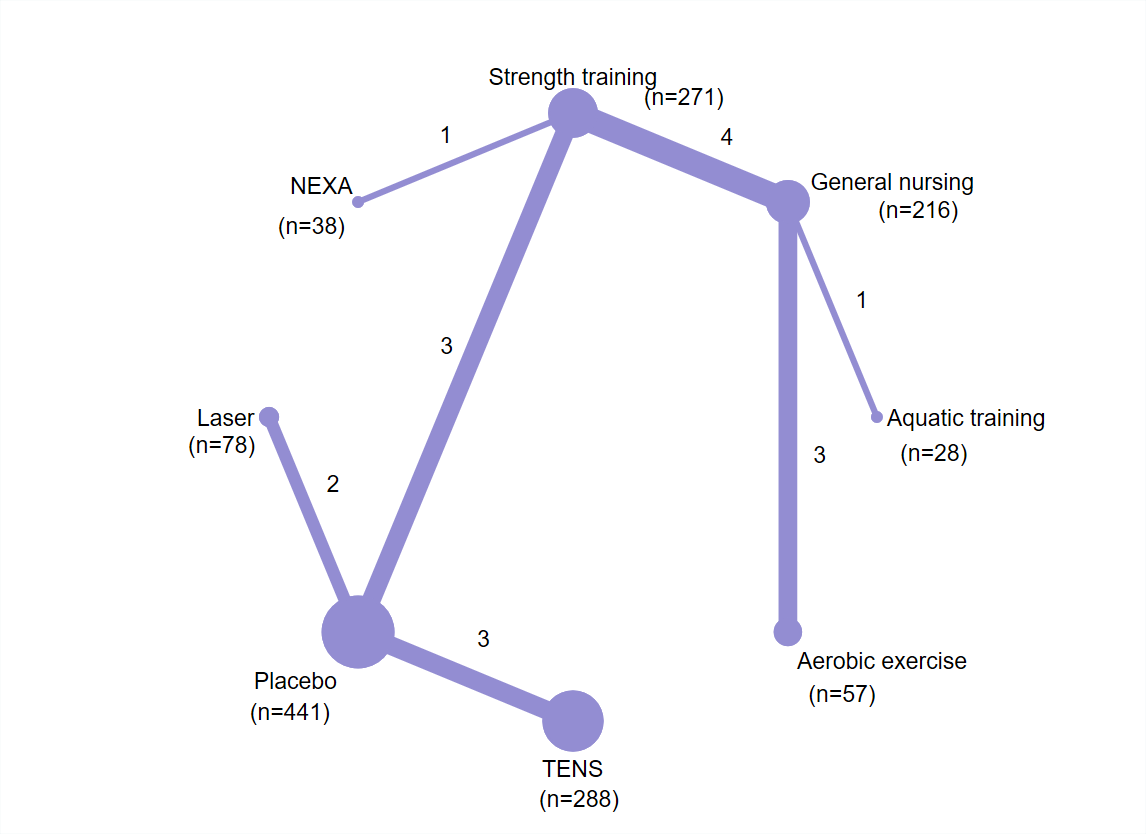


**Figure S6.9:** Network map of WOMAC stiffness at <1 month.


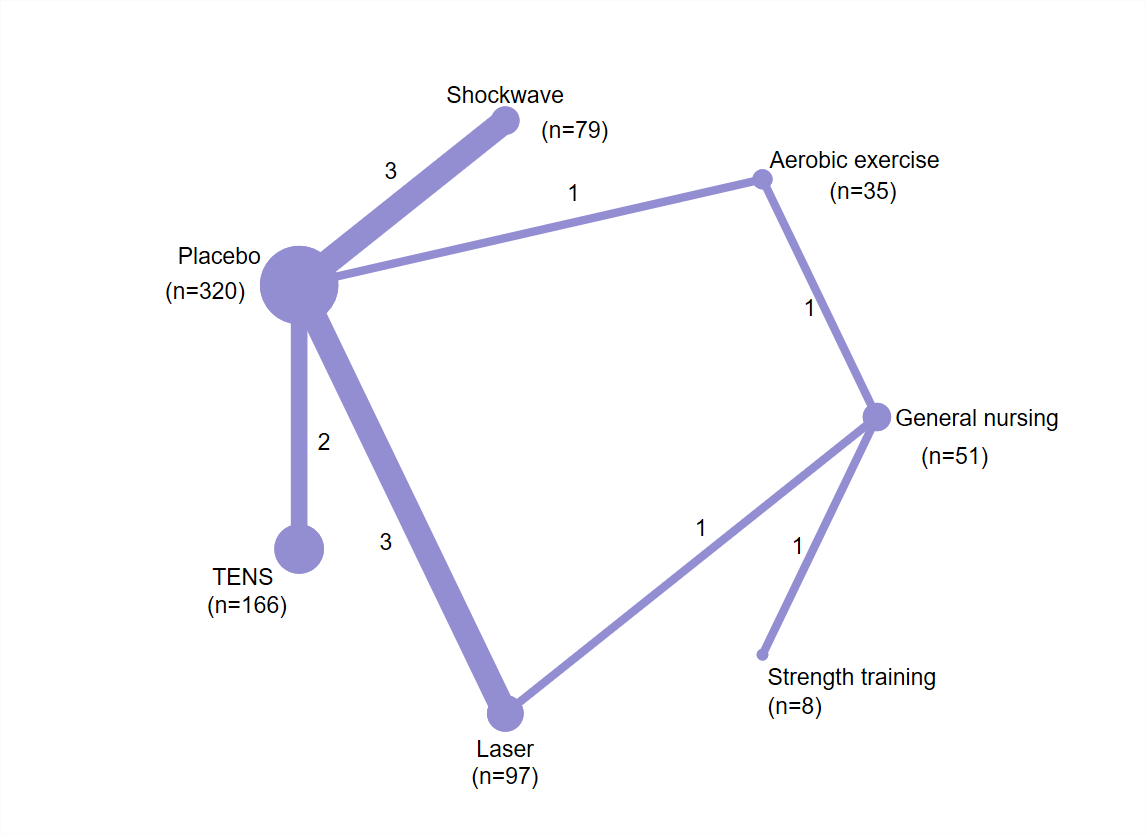


**Figure S6.10:** Network map of WOMAC stiffness at 1-3 months.


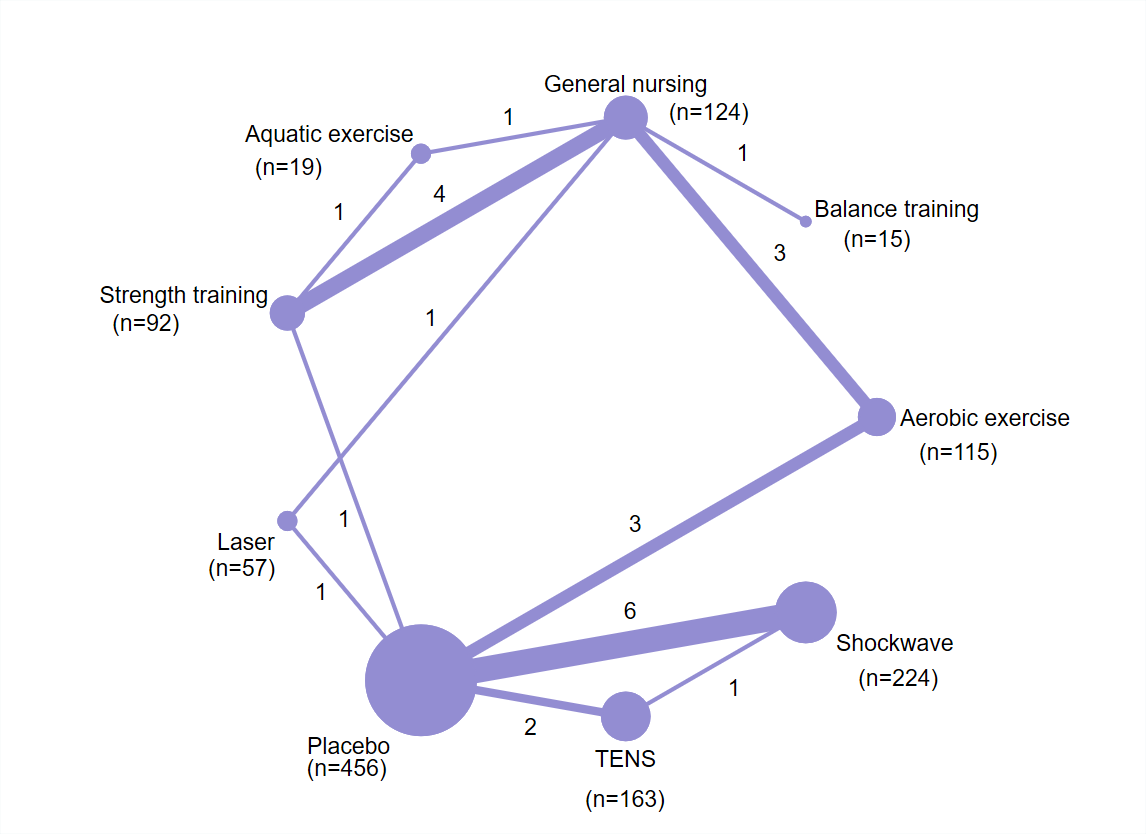


**Figure S6.11:** Network map of WOMAC stiffness at >3 months.


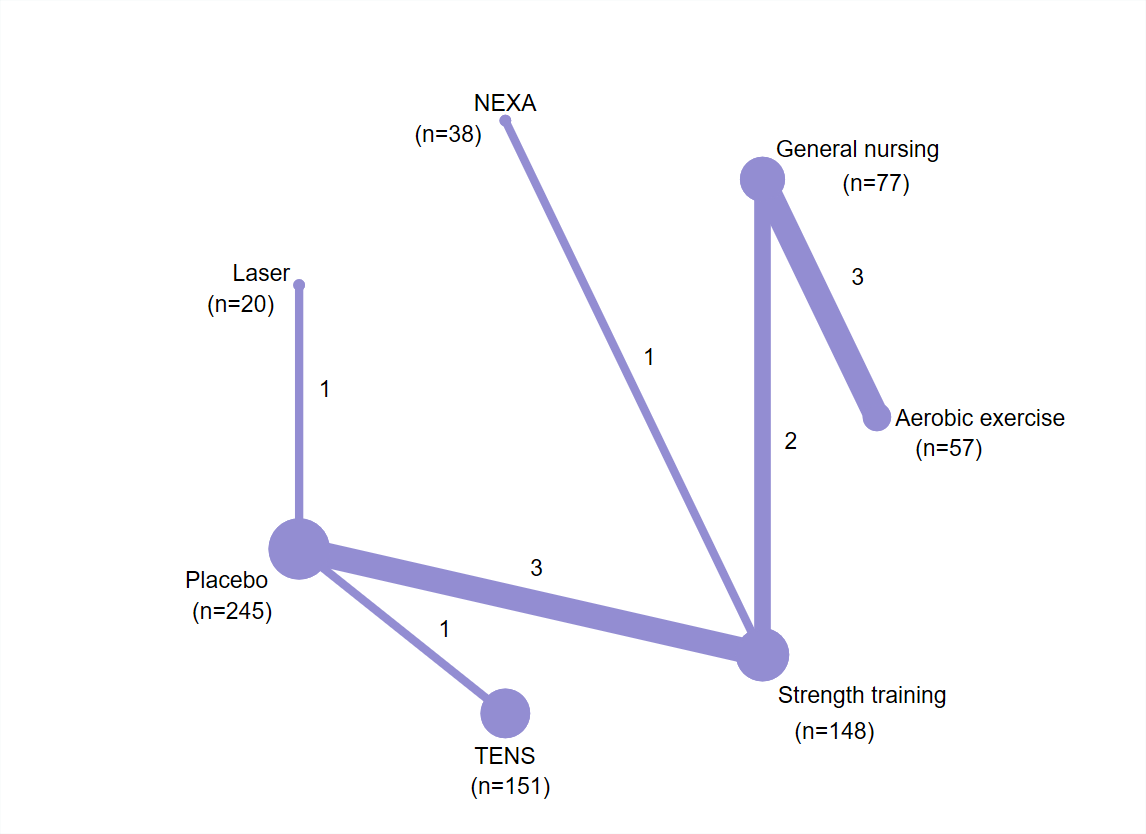


**Figure S6.12:** Network map of WOMAC function at <1 month.


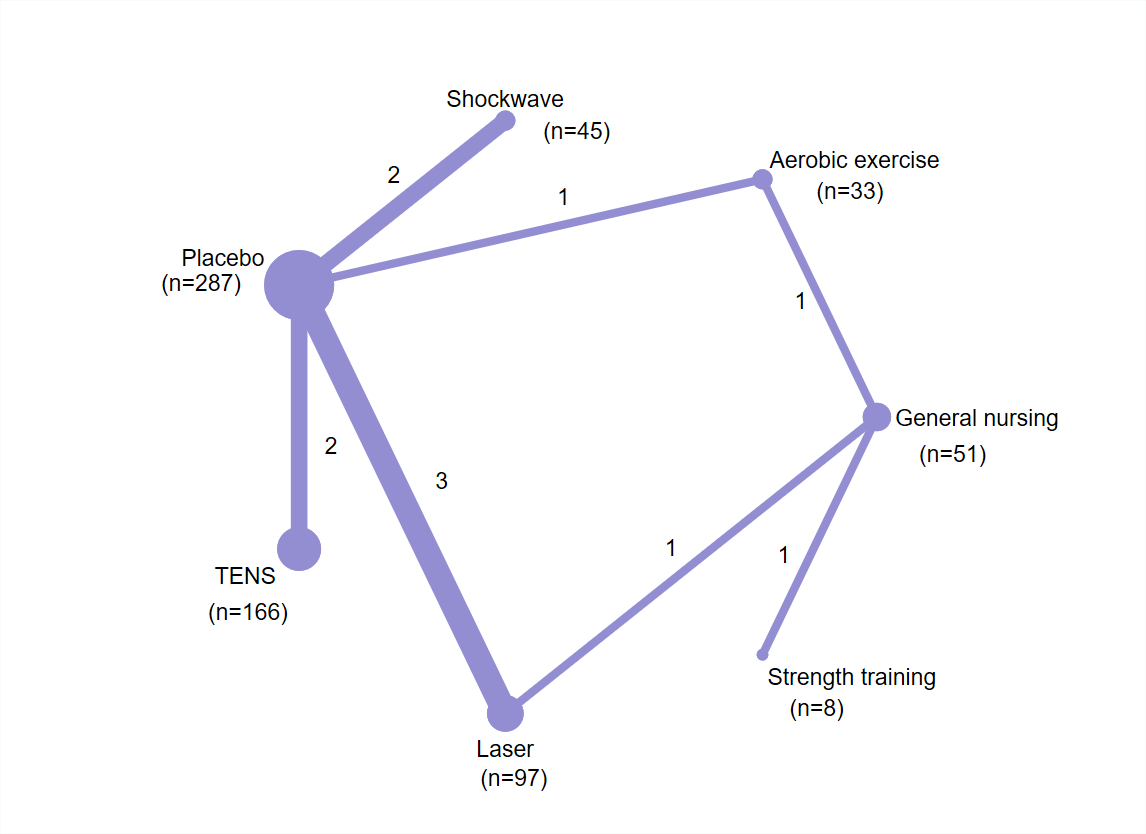


**Figure S6.13:** Network map of WOMAC function at 1-3 months.


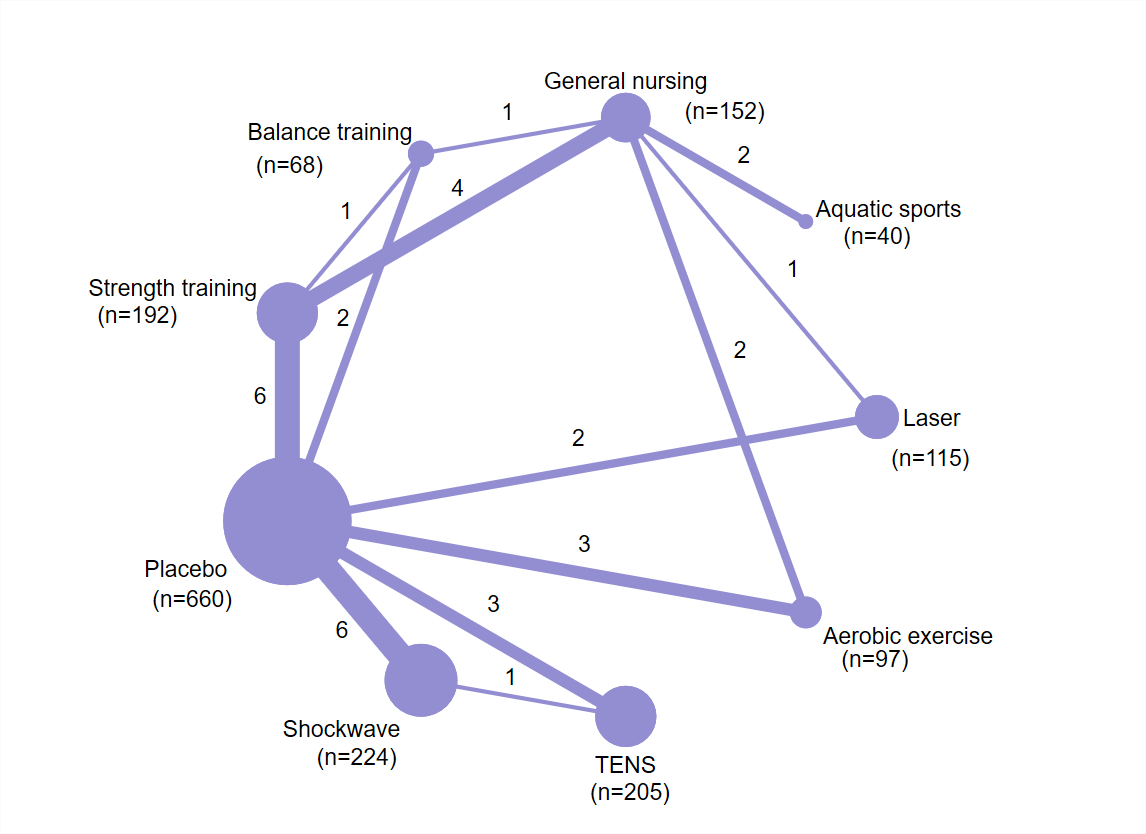


**Figure S6.14:** Network map of WOMAC function at >3 months.


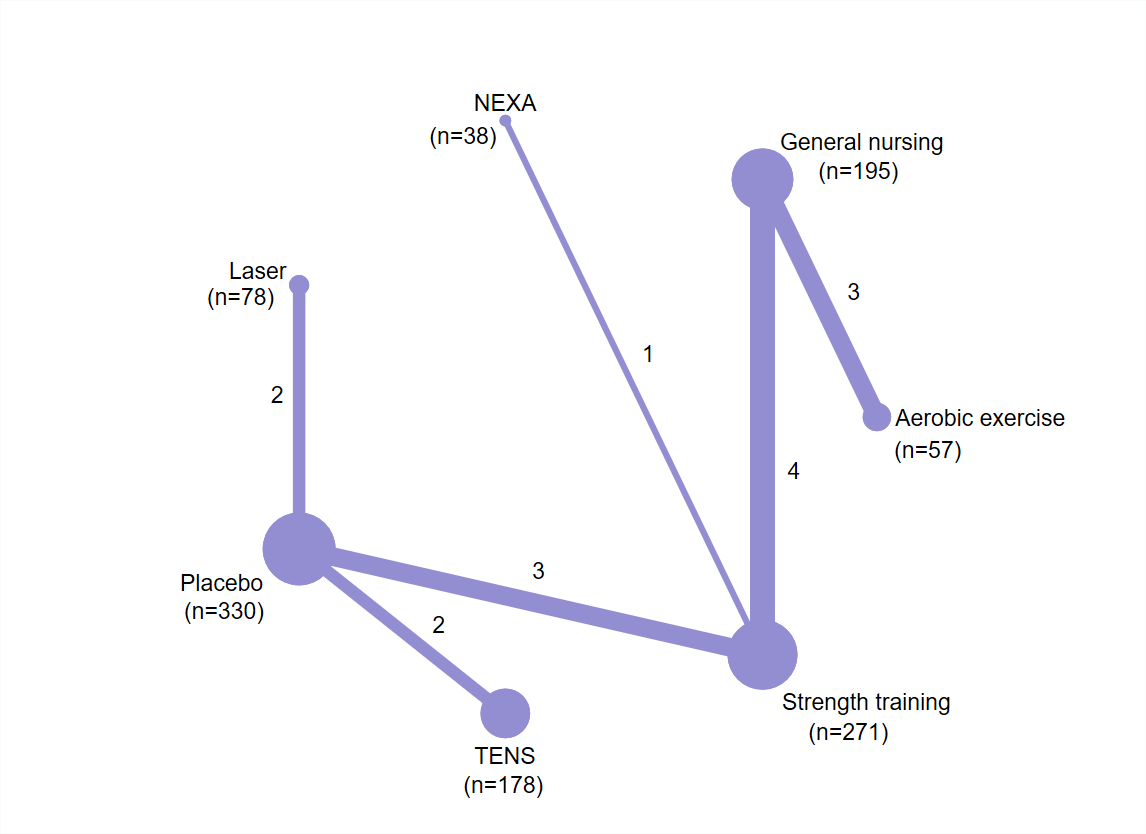


**Figure S6.15:** Network map of WOMAC total score at <1 month.


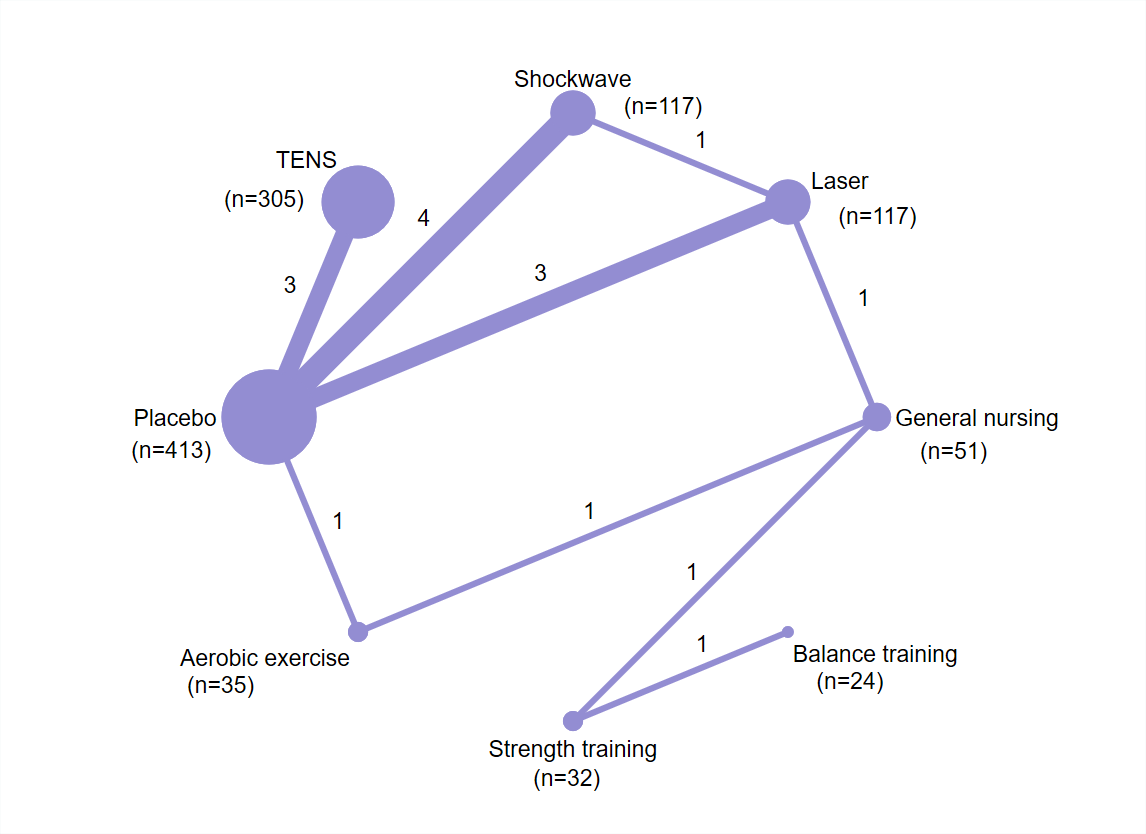


**Figure S6.16:** Network map of WOMAC total score at 1-3 months.


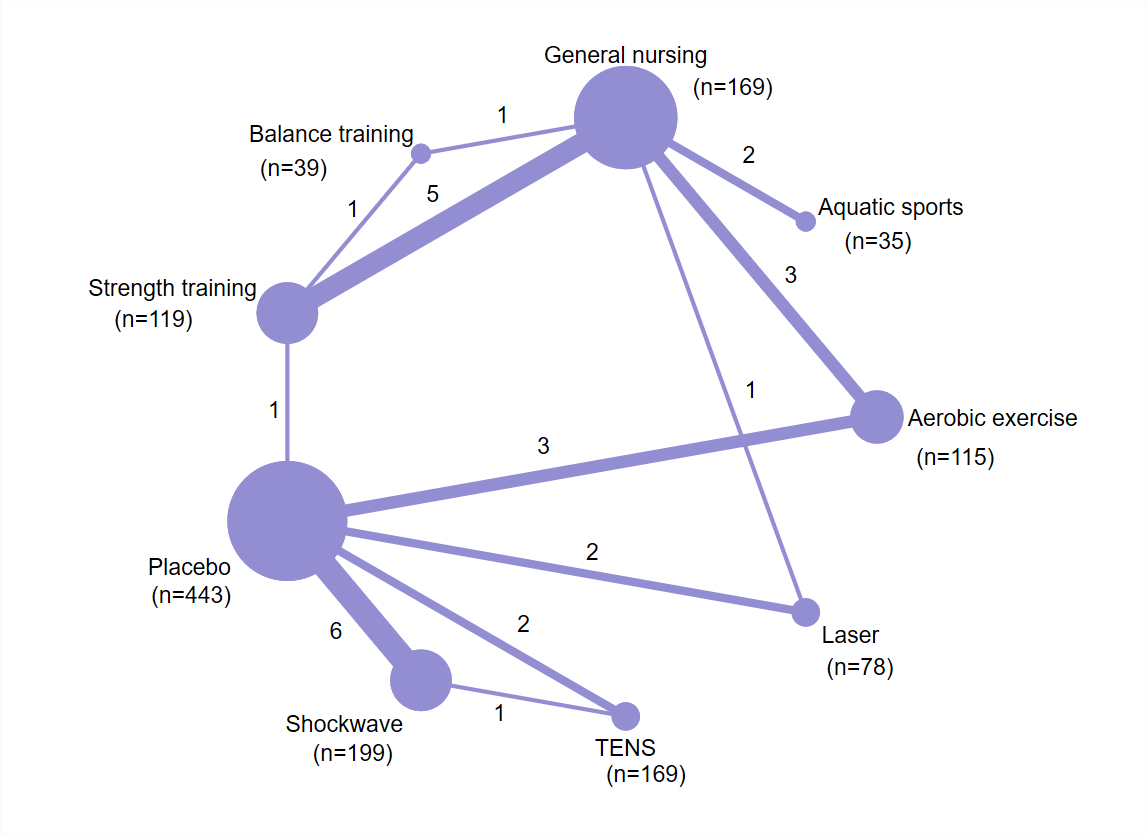


**Figure S6.17:** Network map of WOMAC total score at >3 months.


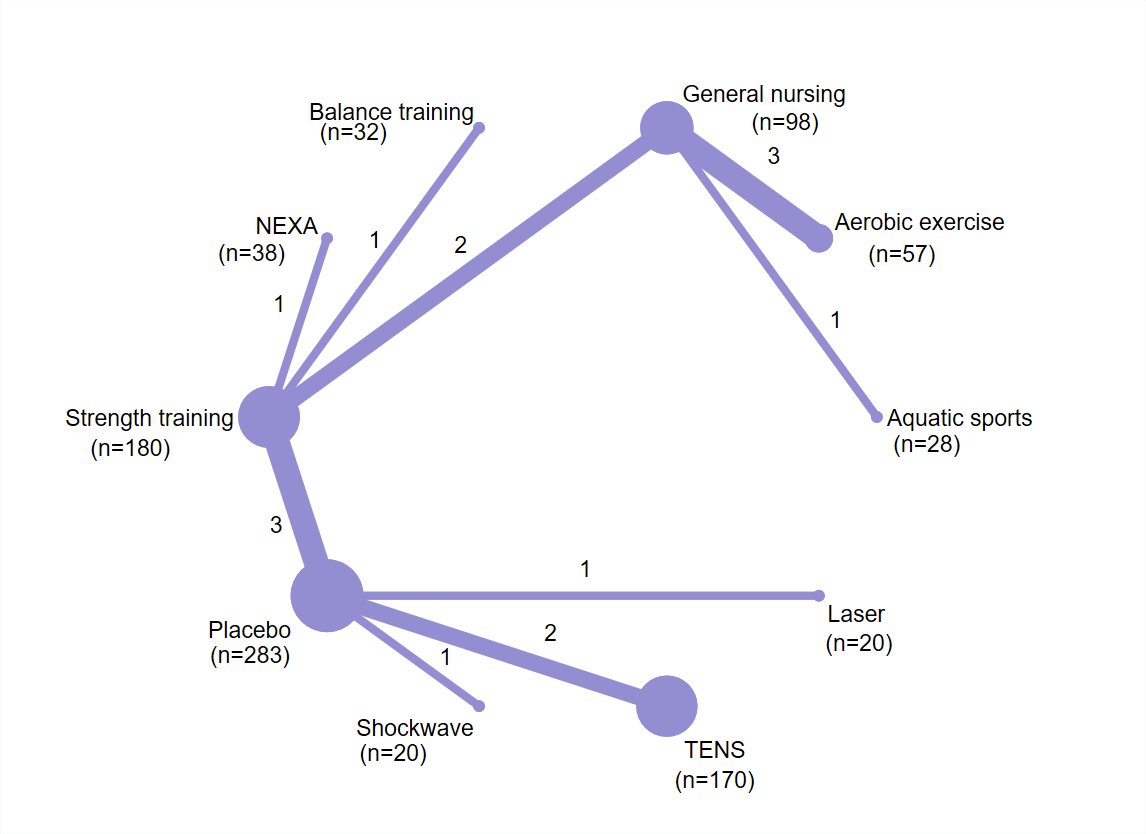


**Appendix 7: Funnel plots**

We used Begg's test to assess the symmetry of the funnel plot, if P > 0.05, the funnel plot is proved to be symmetrical and may not have publication bias; and if P < 0.05.

**Figure S7.1:** Funnel plot of VAS pain during walking at less than 1 month.


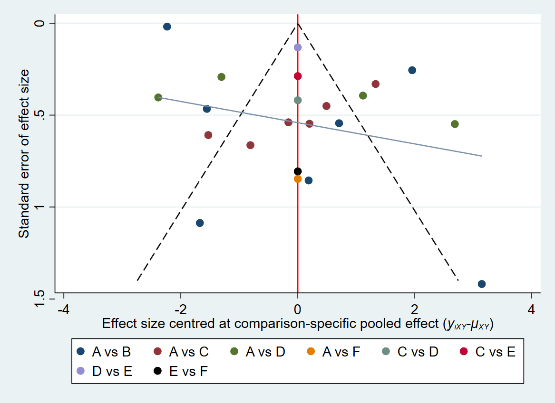


Egger's test t=4.94 P<0.0001

Abbreviations: A, Placebo; B, Transcutaneous Electrical Nerve Stimulation; C, Shockwave;

D, Laser; E, General nursing; F, Aerobic exercise.

**Figure S7.2:** Funnel plot of VAS pain during walking at 1-3 months.


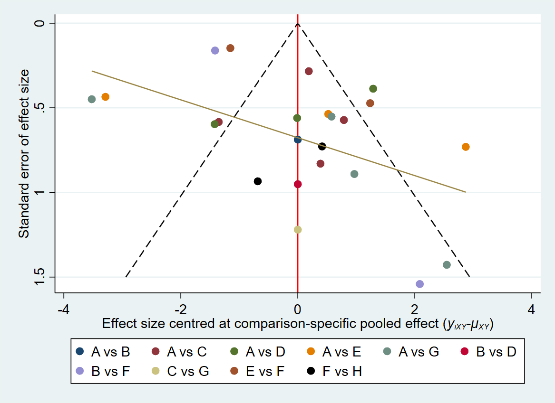


Egger's test t=-1.98 P=0.0607

Abbreviations: A, Placebo; B, Aquatic sports; C, Shockwave; D, Strength training; E, Laser; F, General nursing; G, Transcutaneous Electrical Nerve Stimulation; H, Aerobic exercise.

**Figure S7.3:** Funnel plot of VAS pain during walking at more than 3 months.


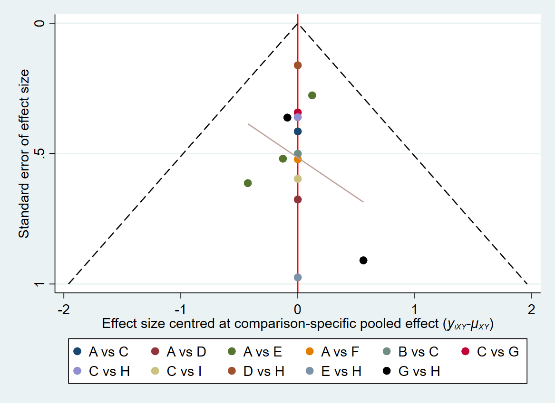


egger's test t=-1.48 P=0.1641

Abbreviations: A, Placebo; B, Neuromuscular exercise; C, Strength training; D, Aquatic sports; E, Transcutaneous Electrical Nerve Stimulation; F, Laser; G, Aerobic exercise;

H, General nursing; I, Balance training.

**Figure S7.4:** Funnel plot of WOMAC pain <1 month.


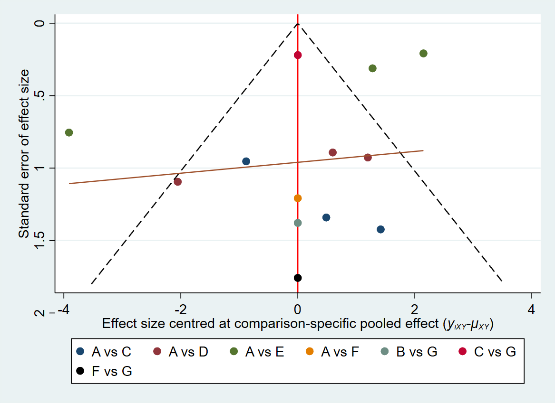


egger's test t=-1.48 P=0.1670

Abbreviations: A, Placebo; B, Strength training; C, Laser; D, Shockwave; E, Transcutaneous Electrical Nerve Stimulation; F, Aerobic exercise; G, General nursing.

**Figure S7.5:** Funnel plot of WOMAC pain 1-3 months.


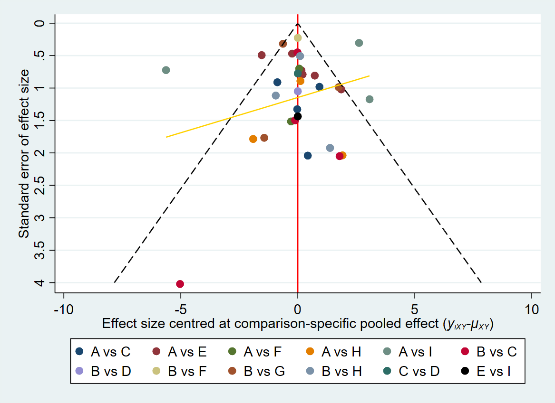


egger's test t=-0.85 P=0.4011

Abbreviations: A, Placebo; B, General nursing; C, Strength training; D, Balance training;

E, Shockwave; F, Laser; G, Aquatic sports; H, Aerobic exercise; I, Transcutaneous Electrical Nerve Stimulation.

**Figure S7.6:** Funnel plot of WOMAC pain >3 months.


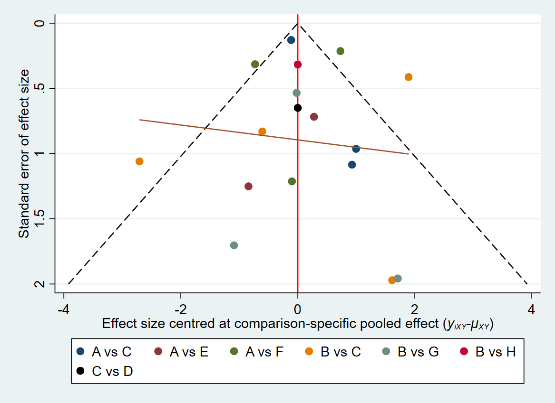


egger's test t=0.84 P=0.4130

Abbreviations: A, Placebo; B, General nursing; C, Strength training; D, Neuromuscular exercise; E, Laser; F, Transcutaneous Electrical Nerve Stimulation; G, Aerobic exercise;

H, Aquatic sports.

**Figure S7.7:** Funnel plot of WOMAC stiffness <1 month.


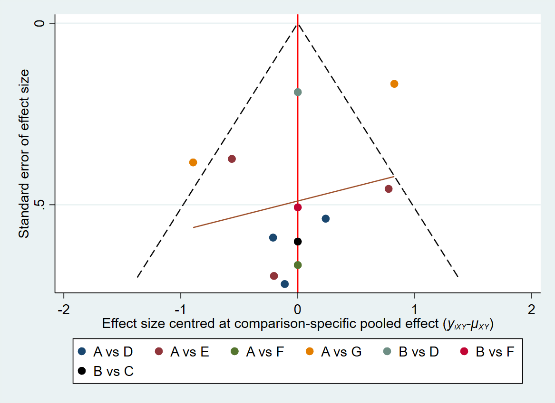


egger's test t=-1.57 P=0.1472

Abbreviations: A, Placebo; B, General nursing; C, Strength training; D, Laser;

E, Shockwave; F, Aerobic exercise; G, Transcutaneous Electrical Nerve Stimulation.

**Figure S7.8:** Funnel plot of WOMAC stiffness 1-3 months.


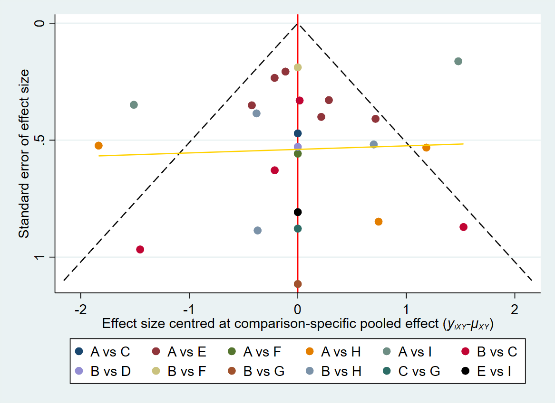


egger's test t=-1.29 P=0.2094

Abbreviations: A, Placebo; B, General nursing; C, Strength training; D, Balance training;

E, Shockwave; F, Laser; G, Aquatic sports; H, Aerobic exercise; I, Transcutaneous Electrical Nerve Stimulation.

**Figure S7.9:** Funnel plot of WOMAC stiffness >3 months.


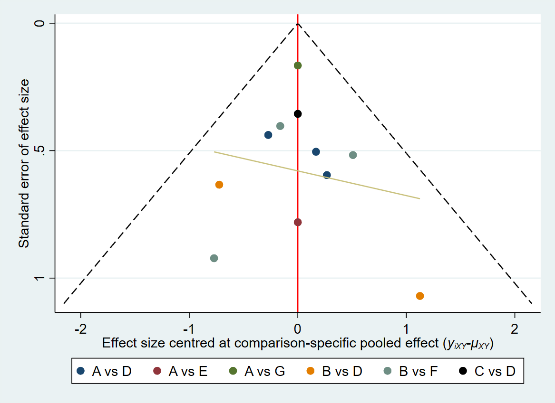


egger's test t=0.77 P=0.4587

Abbreviations: A, Placebo; B, General nursing; C, Neuromuscular exercise; D, Strength training; E, Laser; F, Aerobic exercise; G, Transcutaneous Electrical Nerve Stimulation.

**Figure S7.10:** Funnel plot of WOMAC function <1 month.


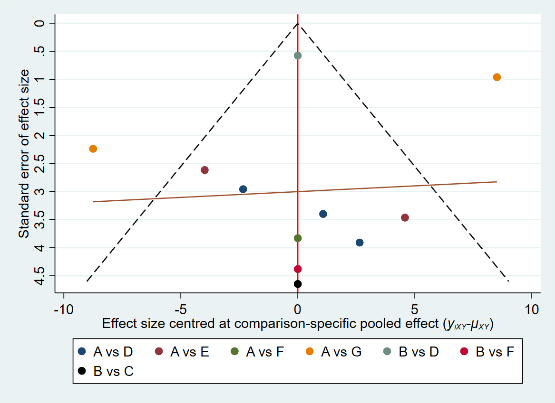


egger's test t=0.50 P=0.6293

Abbreviations: A, Placebo; B, General nursing; C, Strength training; D, Laser; E, Shockwave; F, Aerobic exercise; G, Transcutaneous Electrical Nerve Stimulation.

**Figure S7.11:** Funnel plot of WOMAC function 1-3 months.


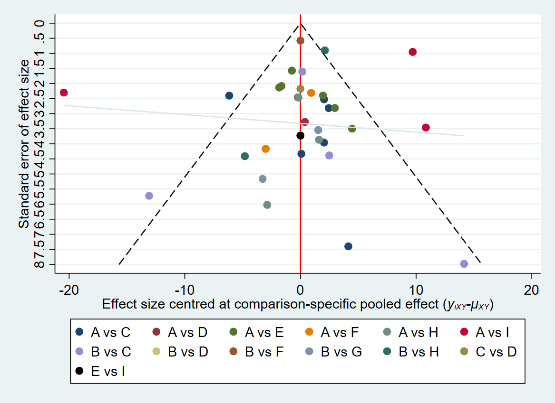


egger's test t=-0.44 P=0.6635

Abbreviations: A, Placebo; B, General nursing; C, Strength training; D, Balance training; E, Shockwave; F, Laser; G, Aquatic sports; H, Aerobic exercise; I, Transcutaneous Electrical Nerve Stimulation.

**Figure S7.12:** Funnel plot of WOMAC function >3 months.


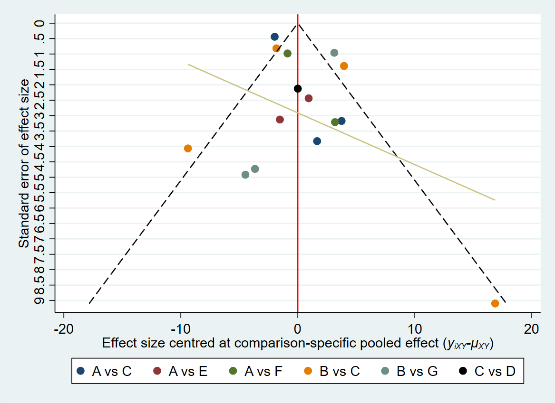


egger's test t=1.60 P=0.1336

Abbreviations: A, Placebo; B, General nursing; C, Strength training; D, Neuromuscular exercise; E, Laser; F, Transcutaneous Electrical Nerve Stimulation; G, Aerobic exercise.

**Figure S7.13:** Funnel plot of WOMAC total score <1 month.


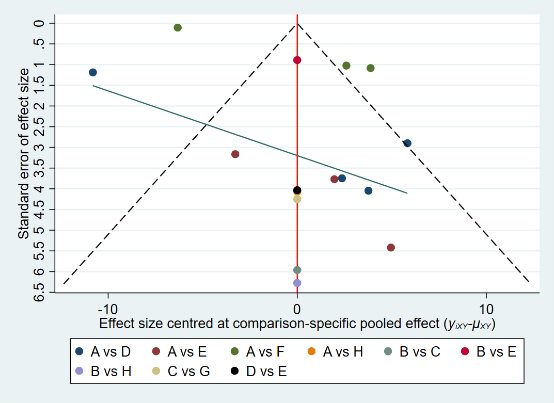


egger's test t=3.02 P=0.0092

Abbreviations: A, Placebo; B, General nursing; C, Strength training; D, Shockwave; E, Laser; F, Transcutaneous Electrical Nerve Stimulation; G, Balance training; H, Aerobic exercise.

**Figure S7.14:** Funnel plot of WOMAC total score 1-3 months.


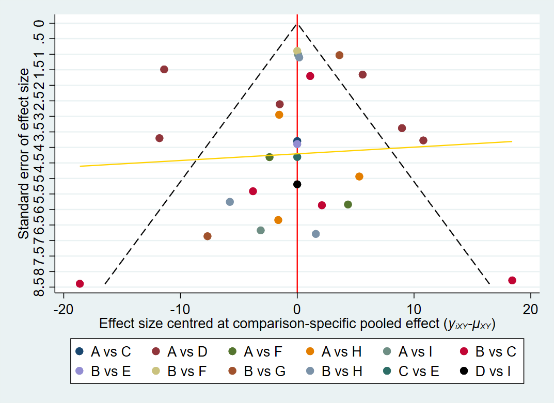


egger's test t=0.75 P=0.4604

Abbreviations: A, Placebo; B, General nursing; C, Strength training; D, Shockwave; E; Balance training; F, Laser; G, Aquatic sports; H, Aerobic exercise; I, Transcutaneous Electrical Nerve Stimulation.

**Figure S7.15:** Funnel plot of WOMAC total score >3 months.


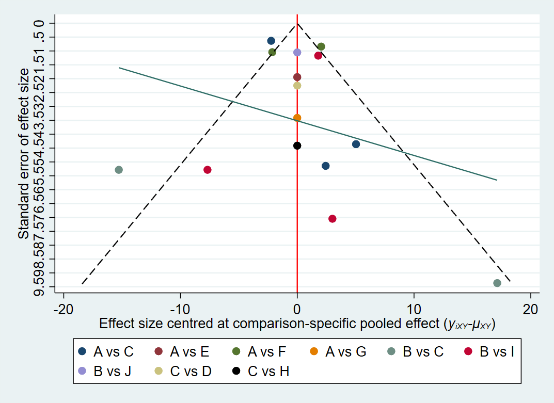


egger's test t=0.87 P=0.4000

Abbreviations: A, Placebo; B, General nursing; C, Strength training; D, Neuromuscular exercise; E, Shockwave; F, Transcutaneous Electrical Nerve Stimulation; G, Laser; H, Balance training; I, Aerobic exercise, J, Aquatic sports.

**Appendix 8: Comprehensive comparisons of different types of physical therapies**

**Figure S8.1:** NRS.


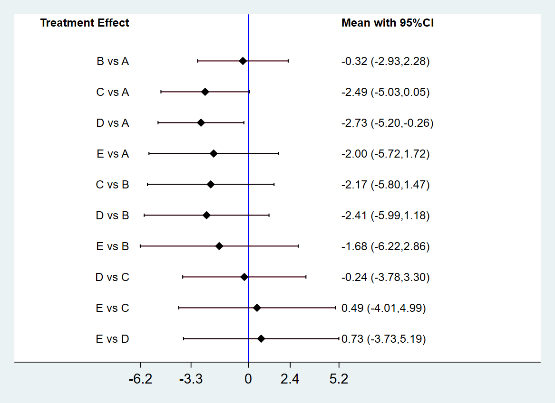


Abbreviations: A, Placebo; B, Laser; C, Shockwave; D, Transcutaneous Electrical Nerve Stimulation; E, Balance training.

**Figure S8.2:** VAS pain at rest.


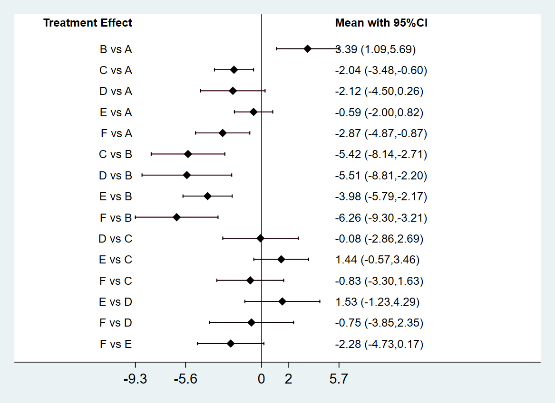


Abbreviations: A, Placebo; B, General nursing; C, Shockwave; D, Aquatic sports; E, Laser;

F, Transcutaneous Electrical Nerve Stimulation.

**Figure S8.3:** VAS pain during walking at less than 1 month.


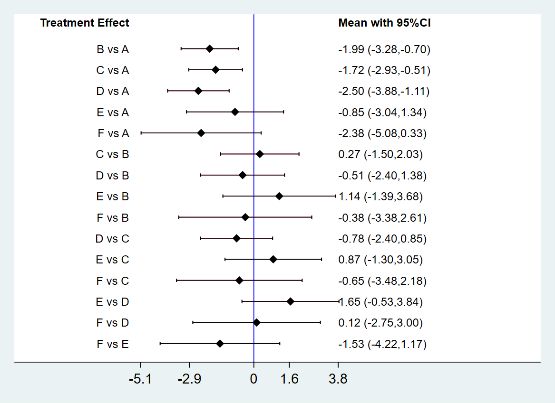


Abbreviations: A, Placebo; B, Transcutaneous Electrical Nerve Stimulation; C, Shockwave;

D, Laser; E, General nursing; F, Aerobic exercise.

**Figure S8.4:** VAS pain during walking at 1-3 months.


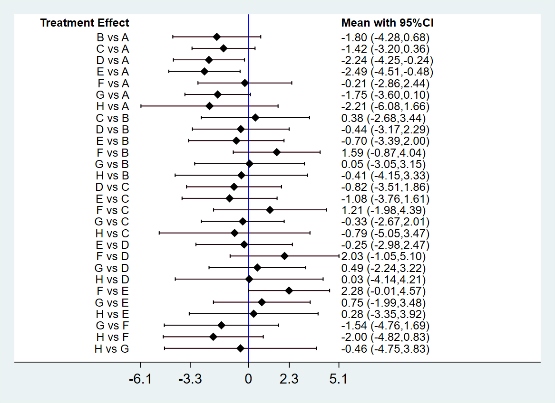


Abbreviations: A, Placebo; B, Aquatic sports; C, Shockwave; D, Strength training; E, Laser;

F, General nursing; G, Transcutaneous Electrical Nerve Stimulation; H, Aerobic exercise.

**Figure S8.5:** VAS pain during walking at more than 3 months.


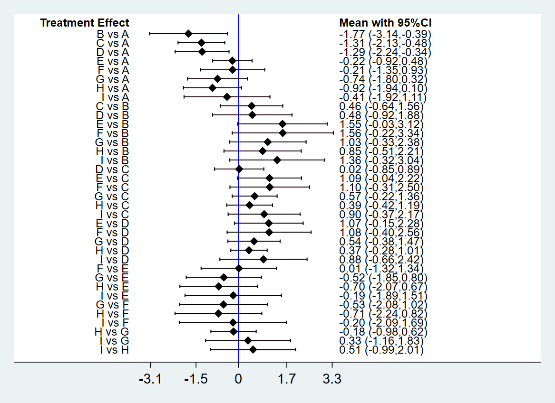


Abbreviations: A, Placebo; B, Neuromuscular exercise; C, Strength training; D, Aquatic sports; E, Transcutaneous Electrical Nerve Stimulation; F, Laser; G, Aerobic exercise;

H, General nursing; I, Balance training.

**Figure S8.6:** WOMAC pain <1 month.


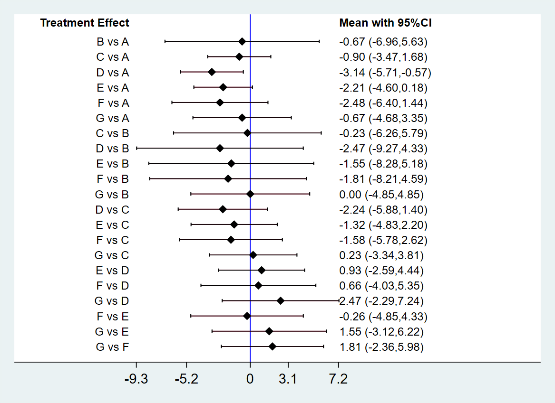


Abbreviations: A, Placebo; B, Strength training; C, Laser; D, Shockwave;

E, Transcutaneous Electrical Nerve Stimulation; F, Aerobic exercise; G, General nursing.

**Figure S8.7:** WOMAC pain 1-3 months.


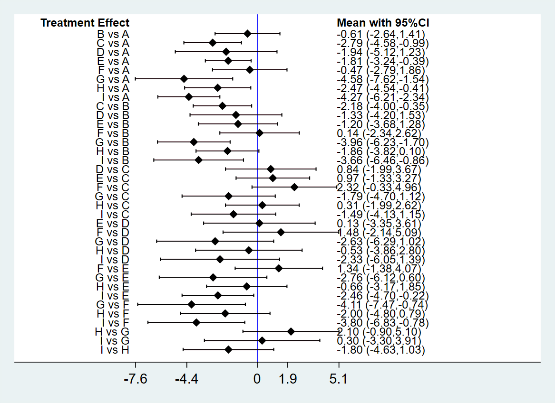


Abbreviations: A, Placebo; B, General nursing; C, Strength training; D, Balance training;

E, Shockwave; F, Laser; G, Aquatic sports; H, Aerobic exercise; I, Transcutaneous Electrical Nerve Stimulation.

**Figure S8.8:** WOMAC pain >3 months.


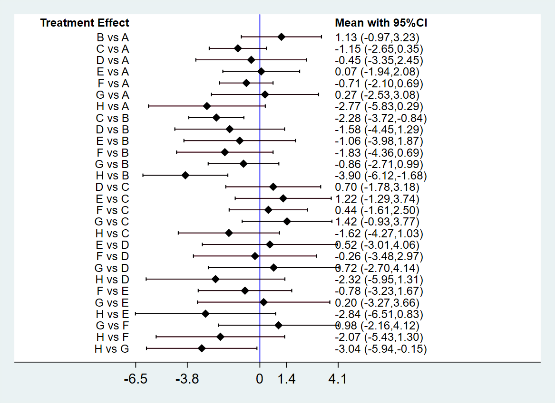


Abbreviations: A, Placebo; B, General nursing; C, Strength training; D, Neuromuscular exercise; E, Laser; F, Transcutaneous Electrical Nerve Stimulation; G, Aerobic exercise;

H, Aquatic training.

**Figure S8.9:** WOMAC stiffness <1 month.


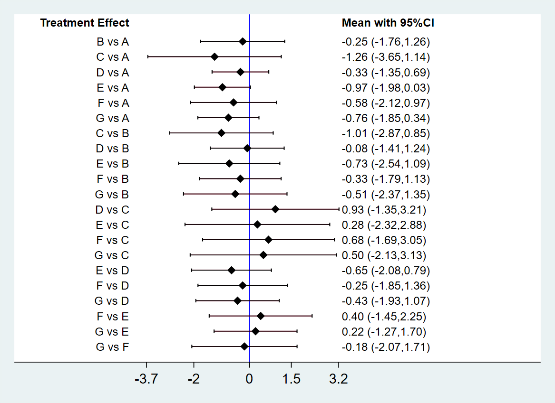


Abbreviations: A, Placebo; B, General nursing; C, Strength training; D, Laser; E, Shockwave; F, Aerobic exercise; G, Transcutaneous Electrical Nerve Stimulation.

**Figure S8.10:** WOMAC stiffness 1-3 months.


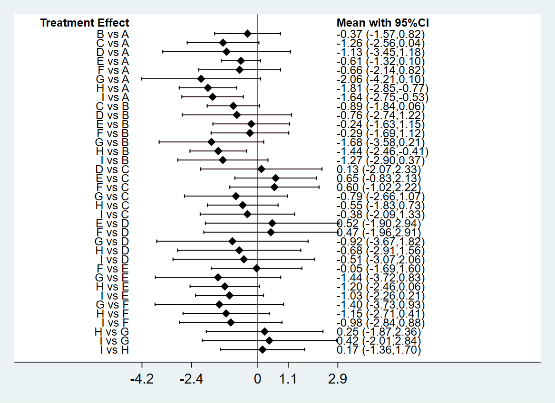


Abbreviations: A, Placebo; B, General nursing; C, Strength training; D, Balance training; E, Shockwave; F, Laser; G, Aquatic sports; H, Aerobic exercise; I, Transcutaneous Electrical Nerve Stimulation.

**Figure S8.11:** WOMAC stiffness >3 months.


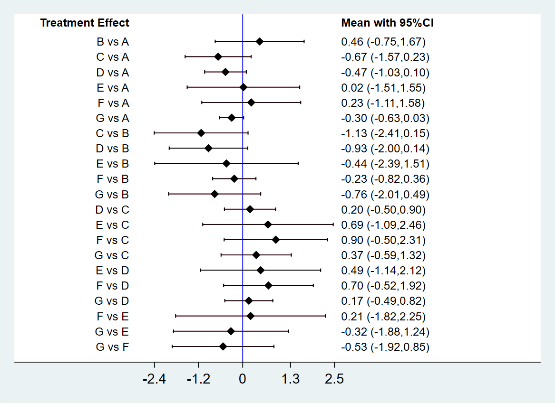


Abbreviations: A, Placebo; B, General nursing; C, Neuromuscular exercise; D, Strength training; E, Laser; F, Aerobic exercise; G, Transcutaneous Electrical Nerve Stimulation.

**Figure S8.12:** WOMAC function <1 month.


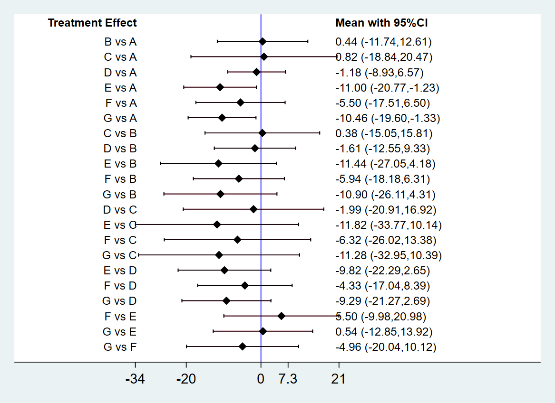


Abbreviations: A, Placebo; B, General nursing; C, Strength training; D, Laser，E, Shockwave; F, Aerobic exercise; G, Transcutaneous Electrical Nerve Stimulation.

**Figure S8.13:** WOMAC function 1-3 months.


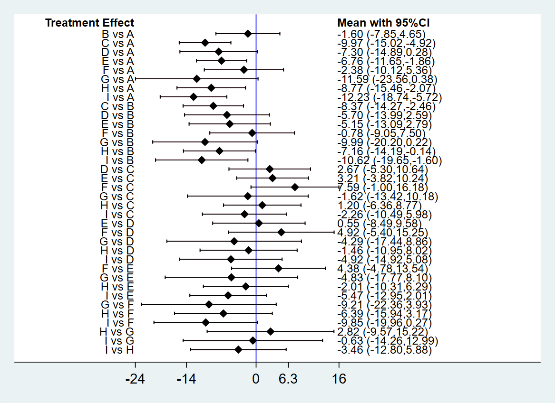


Abbreviations: A, Placebo; B, General nursing; C, Strength training; D, Balance training; E, Shockwave; F, Laser; G, Aquatic sports; H, Aerobic exercise; I, Transcutaneous Electrical Nerve Stimulation.

**Figure S8.14:** WOMAC function >3 months.


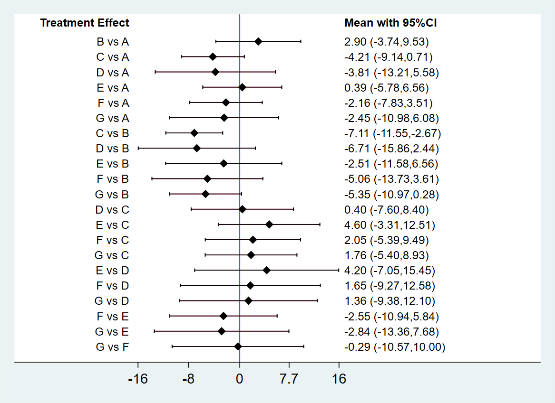


Abbreviations: A, Placebo; B, General nursing; C, Strength training; D, Neuromuscular exercise; E, Laser; F, Transcutaneous Electrical Nerve Stimulation; G, Aerobic exercise.

**Figure S8.15:** WOMAC total score <1 month.


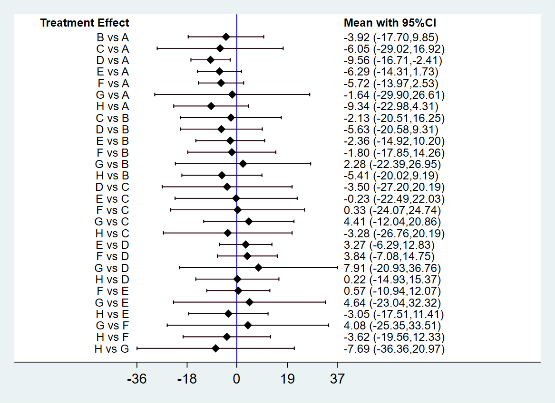


Abbreviations: A, Placebo; B, General nursing; C, Strength training; D, Shockwave; E, Laser; F, Transcutaneous Electrical Nerve Stimulation; G, Balance training; H, Aerobic exercise.

**Figure S8.16:** WOMAC total score 1-3 months.


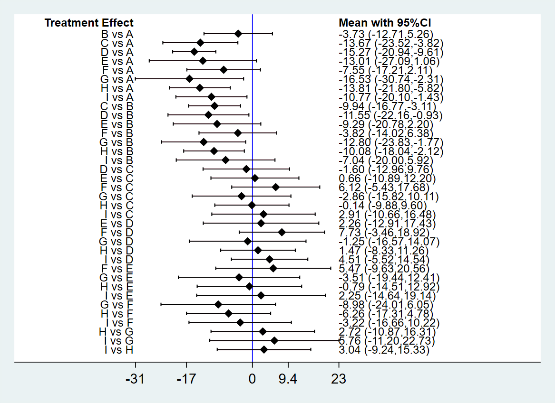


Abbreviations: A, Placebo; B, General nursing; C, Strength training; D, Shockwave; E; Balance training; F, Laser; G, Aquatic sports; H, Aerobic exercise; I, Transcutaneous Electrical Nerve Stimulation.

**Figure S8.17:** WOMAC total score >3 months.


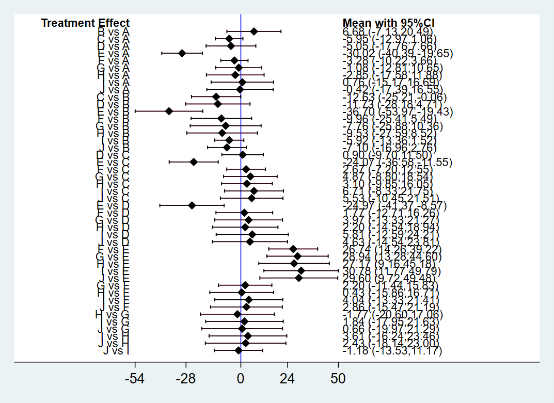


Abbreviations: A, Placebo; B, General nursing; C, Strength training; D, Neuromuscular exercise; E, Shockwave; F, Transcutaneous Electrical Nerve Stimulation; G, Laser; H, Balance training; I, Aerobic exercise; J, Aquatic sports.

**Appendix 9: Cumulative probability plots**

Sensitivity analysis is conducted by excluding literature one by one.

| **Outcome** | **tau^2^** | **Sources of heterogeneity** | **tau^2*^** |
| --- | --- | --- | --- |
| VAS pain on walking at < 1 month | 4.3318 | Lv2019、Atamaz2012、Reichenbach2022、Nambi S2017、ELGENDY2022 | 1.2123 |
| VAS pain during walking at 1-3 months | 3.1067 | YURTKURAN2007、Mascarin2012、Nambi S2017、Gundog M2012 | 1.0994 |
| VAS pain on walking at > 3 months | 0.1235 |  |  |
| WOMAC pain <1 month | 2.9911 | Külcü2009、Gundog M2012 | 0.2006 |
| WOMAC pain 1-3 months | 3.9891 | Mascarin2012、Gundog M2012 | 0.5148 |
| WOMAC pain > 3 month | 1.1944 | Song2022、Zhu2017 | 0.5093 |
| WOMAC stiffness <1month | 0.7182 | Gundog M2012 | 0.2830 |
| WOMAC stiffness 1-3 months | 0.8162 | Tu2021 | 0.2825 |
| WOMAC stiffness > 3 month | 0 |  |  |
| WOMAC function <1month | 71.9499 | Gundog M2012 | 3.9891 |
| WOMAC function 1-3 months | 47.8414 | Tu2021、Bruce-Brand2012、 Pazit2018 | 24.3007^#^ |
| WOMAC function > 3 month | 13.3063 | Messier2021、Almeida2021、Song2022、Bruce-Brand2012 | 1.3123 |
| WOMAC total score <1month | 53.8205 | Elerian2016、Mostafa2022、Lv2019 | 0 |
| WOMAC total score 1-3 months | 59.7765 | Elerian2016、Karakaş2020、Özgönenel2018、Mascarin2012、Pazit2018 | 4.7366 |
| WOMAC total score >3 months | 13.7867 | Almeida2021、Zhu2017、Topp2002 | 5.8033 |

*: Heterogeneity after sensitivity analysis.

#: When heterogeneity is smaller, global consistency cannot pass.

The larger the surface under the cumulative ranking curve, the better the effect.

Abbreviations: NEXA, Neuromuscular exercise; TENS, Transcutaneous Electrical Nerve Stimulation.

**Figure S9.1:** Cumulative ranking curve plots of NRS.


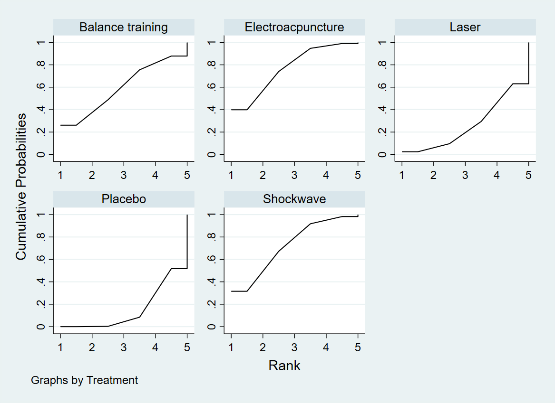


**Figure S9.2:** Cumulative ranking curve plots of VAS pain at rest.


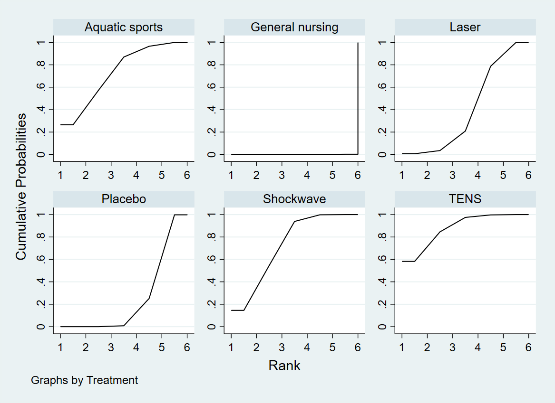


**Figure S9.3:** Cumulative ranking curve plots of VAS pain during walking at less than 1 month.


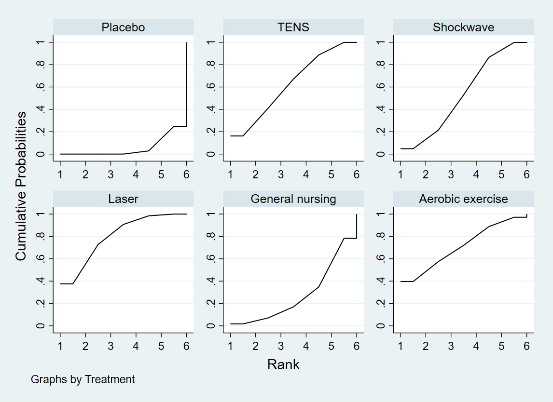


**Figure S9.4:** Cumulative ranking curve plots of VAS pain during walking at 1-3 months.
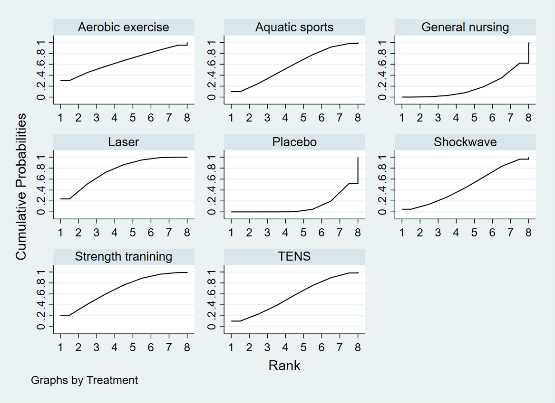


**Figure S9.5:** Cumulative ranking curve plots of VAS pain during walking at more than 3 months.
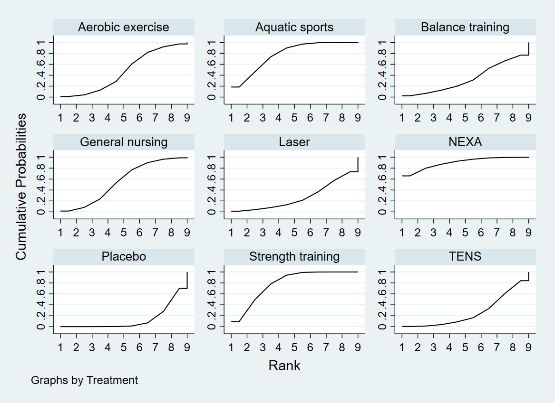


**Figure S9.6:** Cumulative ranking curve plots of WOMAC pain < 1 month.


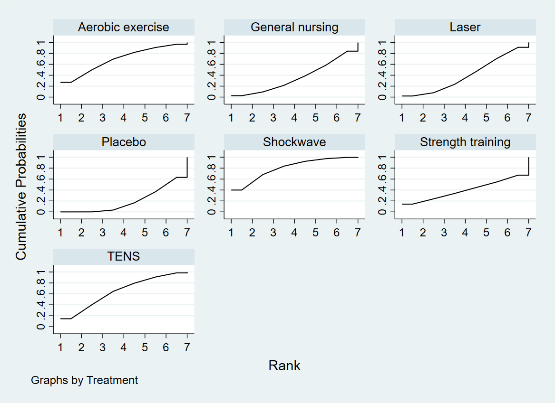


**Figure S9.7:** Cumulative ranking curve plots of WOMAC pain 1-3 months.


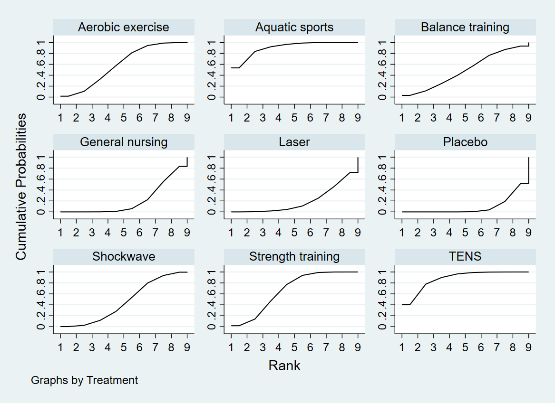


**Figure S9.8:** Cumulative ranking curve plots of WOMAC pain >3 months.


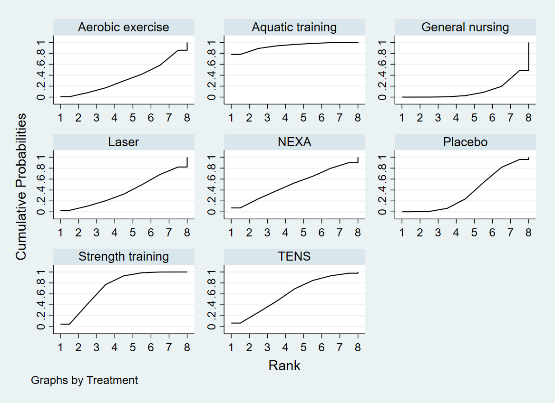


**Figure S9.9:** Cumulative ranking curve plots of WOMAC stiffness < 1 month.


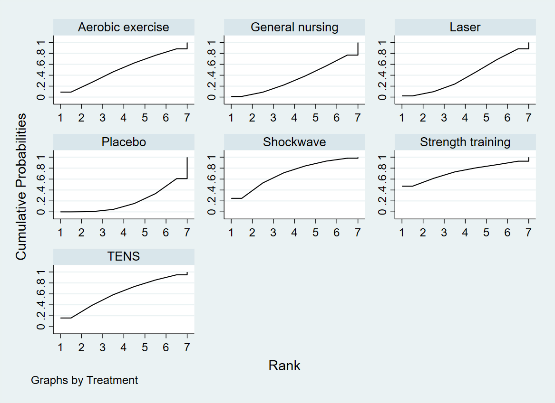


**Figure S9.10:** Cumulative ranking curve plots of WOMAC stiffness 1-3 months.


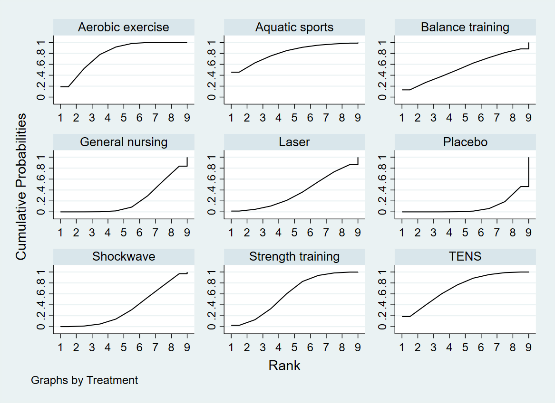


**Figure S9.11:** Cumulative ranking curve plots of WOMAC stiffness >3 months.


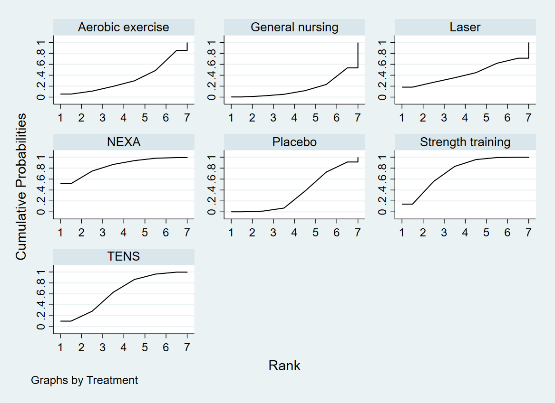


**Figure S9.12:** Cumulative ranking curve plots of WOMAC function < 1 month.


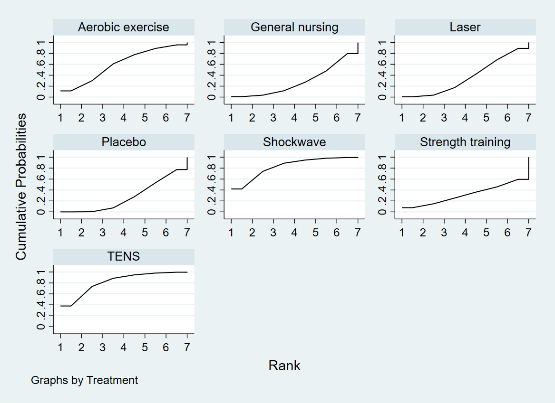


**Figure S9.13:** Cumulative ranking curve plots of WOMAC function 1-3 months.


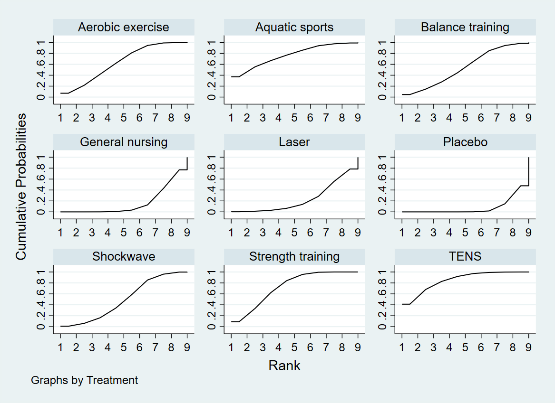


**Figure S9.14:** Cumulative ranking curve plots of WOMAC function >3 months.


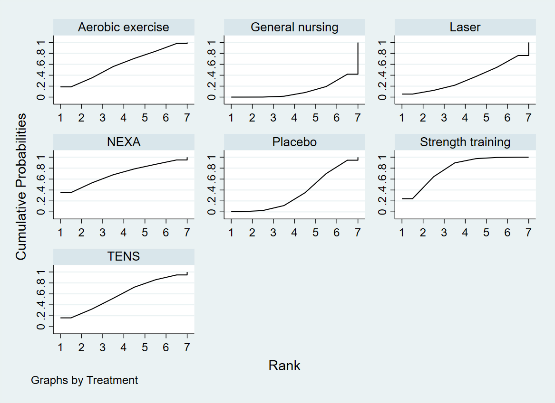


**Figure S9.15:** Cumulative ranking curve plots of WOMAC total score < 1 month.


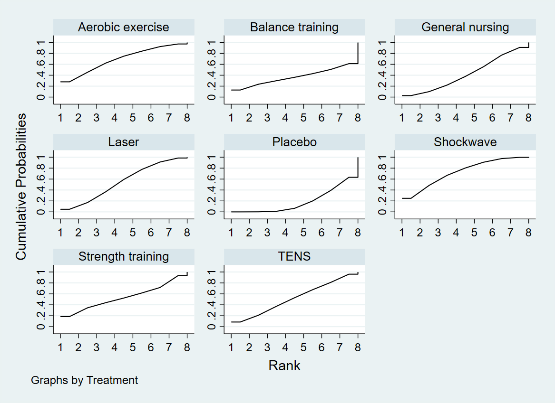


**Figure S9.16:** Cumulative ranking curve plots of WOMAC total score 1-3 months.


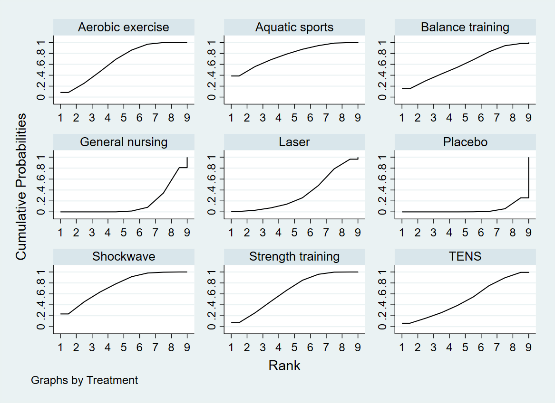


**Figure S9.17:** Cumulative ranking curve plots of WOMAC total score >3 months.


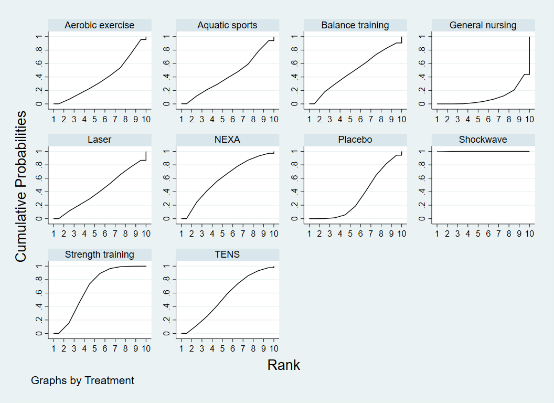


**Appendix 10: The results of sensitivity Analyses**

Abbreviations: NEXA, Neuromuscular exercise; TENS, Transcutaneous Electrical Nerve Stimulation.

**Figure S10.1:** Cumulative ranking curve plots of VAS pain during walking at less than 1 month.


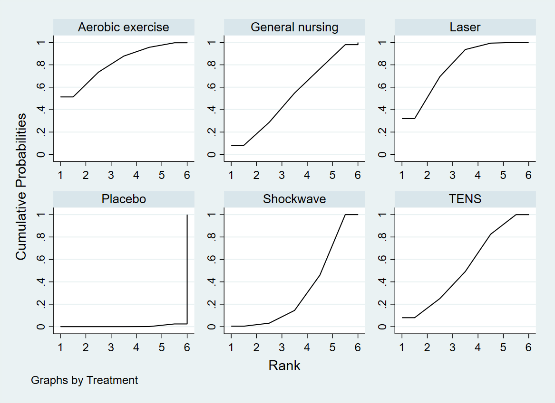


**Figure S10.2:** Cumulative ranking curve plots of VAS pain during walking at 1-3 months.


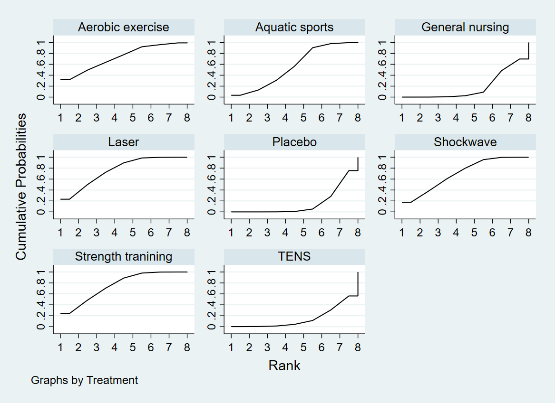


**Figure S10.3:** Cumulative ranking curve plots of WOMAC pain < 1 month.


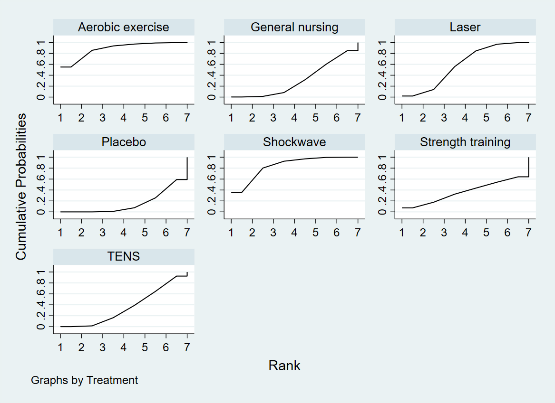


**Figure S10.4:** Cumulative ranking curve plots of WOMAC pain 1-3 months.


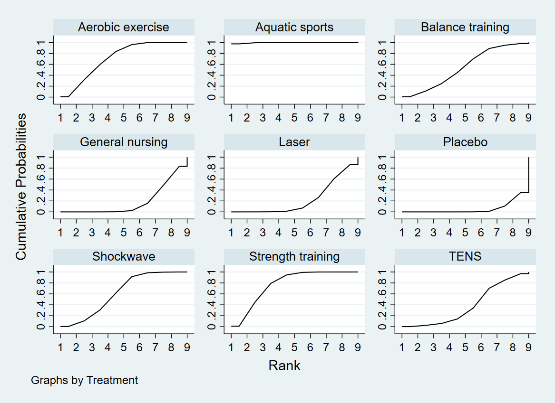


**Figure S10.5:** Cumulative ranking curve plots of WOMAC pain >3 months.


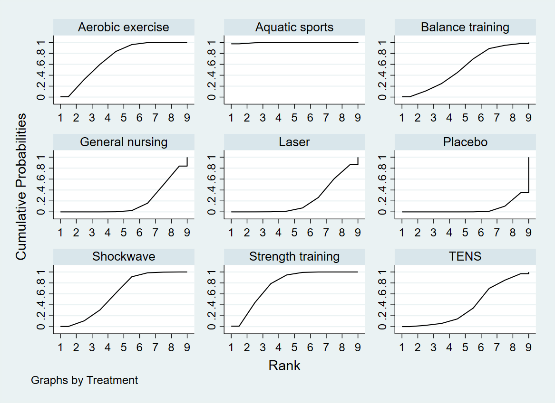


**Figure S10.6:** Cumulative ranking curve plots of WOMAC stiffness < 1 month.


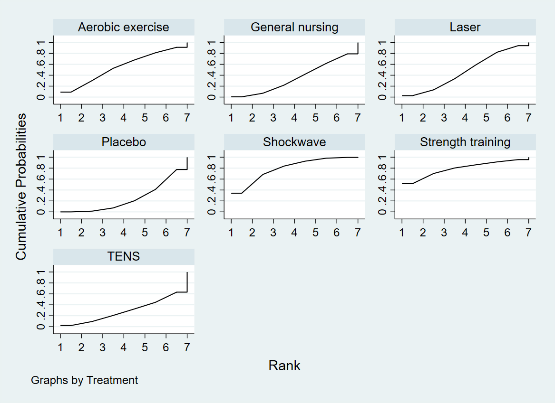


**Figure S10.7:** Cumulative ranking curve plots of WOMAC stiffness 1-3 months.


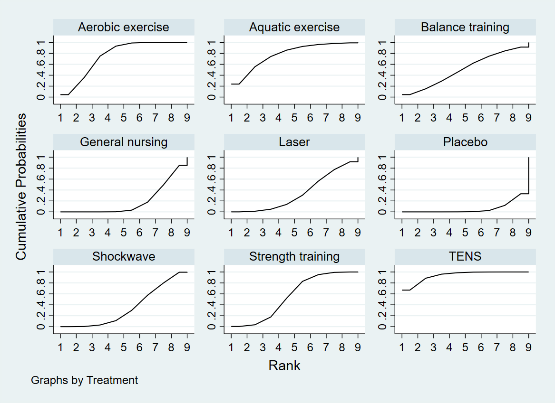


**Figure S10.8:** Cumulative ranking curve plots of WOMAC function < 1 month.


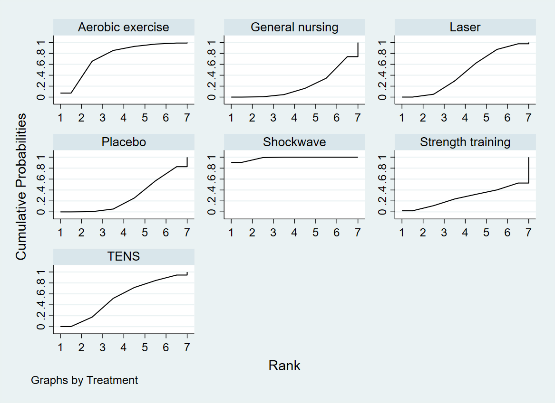


**Figure S10.9:** Cumulative ranking curve plots of WOMAC function 1-3 months.


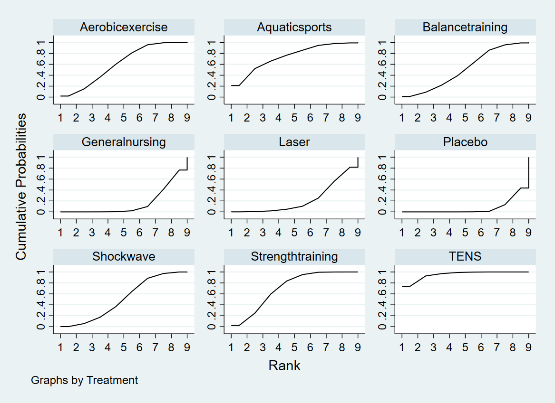


**Figure S10.10:** Cumulative ranking curve plots of WOMAC function >3 months.


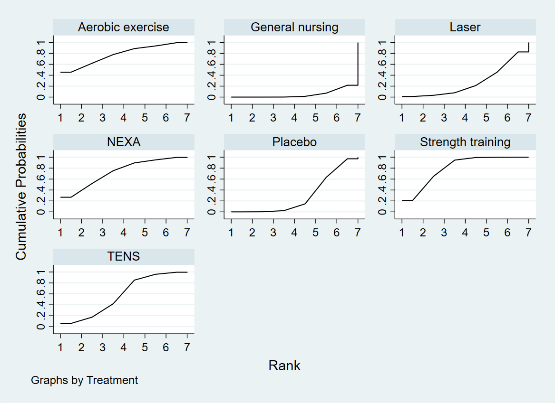


**Figure S10.11:** Cumulative ranking curve plots of WOMAC total score < 1 month.


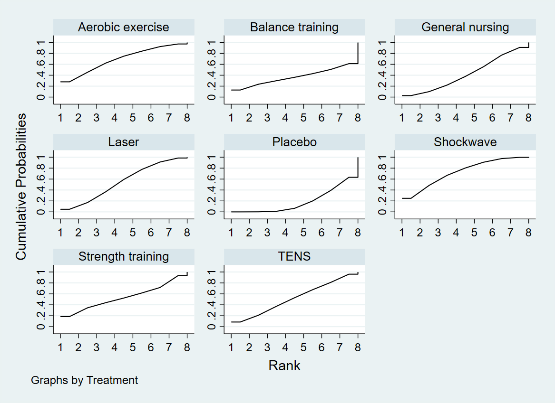


**Figure S10.12:** Cumulative ranking curve plots of WOMAC total score 1-3 months.


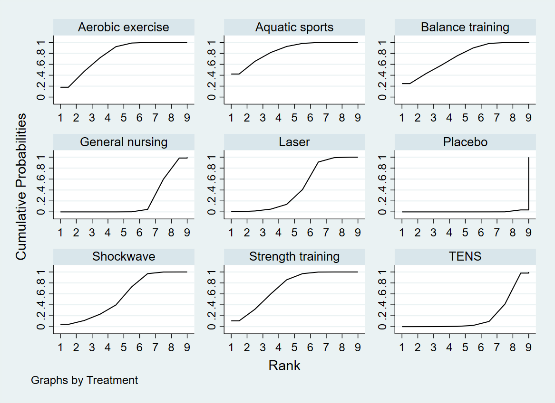


**Figure S10.13:** Cumulative ranking curve plots of WOMAC total score >3 months.


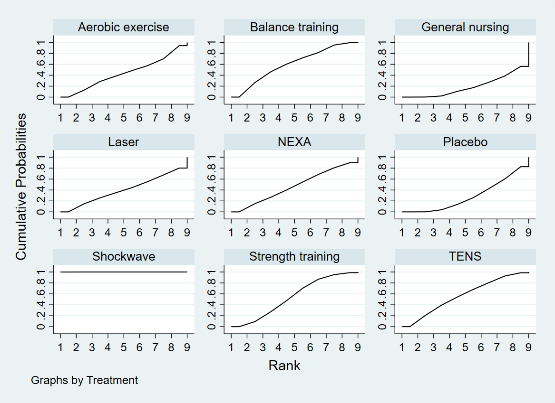


**Figure S10.14:** Forest plot of network effect sizes between the various interventions of VAS pain on walking at less than 1 month.


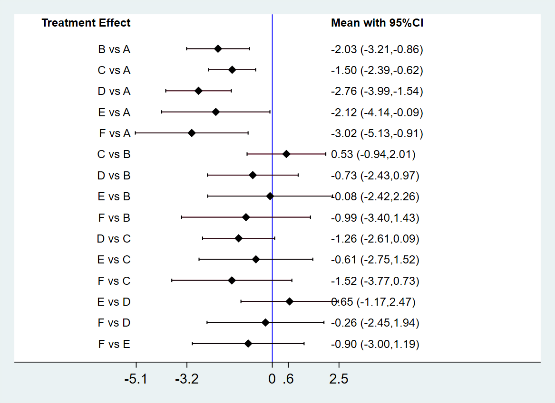


Abbreviations: A, Placebo; B, Transcutaneous Electrical Nerve Stimulation; C, Shockwave;

D, Laser; E, General nursing; F, Aerobic exercise.

A sensitivity network meta-analysis with low risk of bias for the VAS pain during walking at 1-3 months

**Figure S10.15:**network map


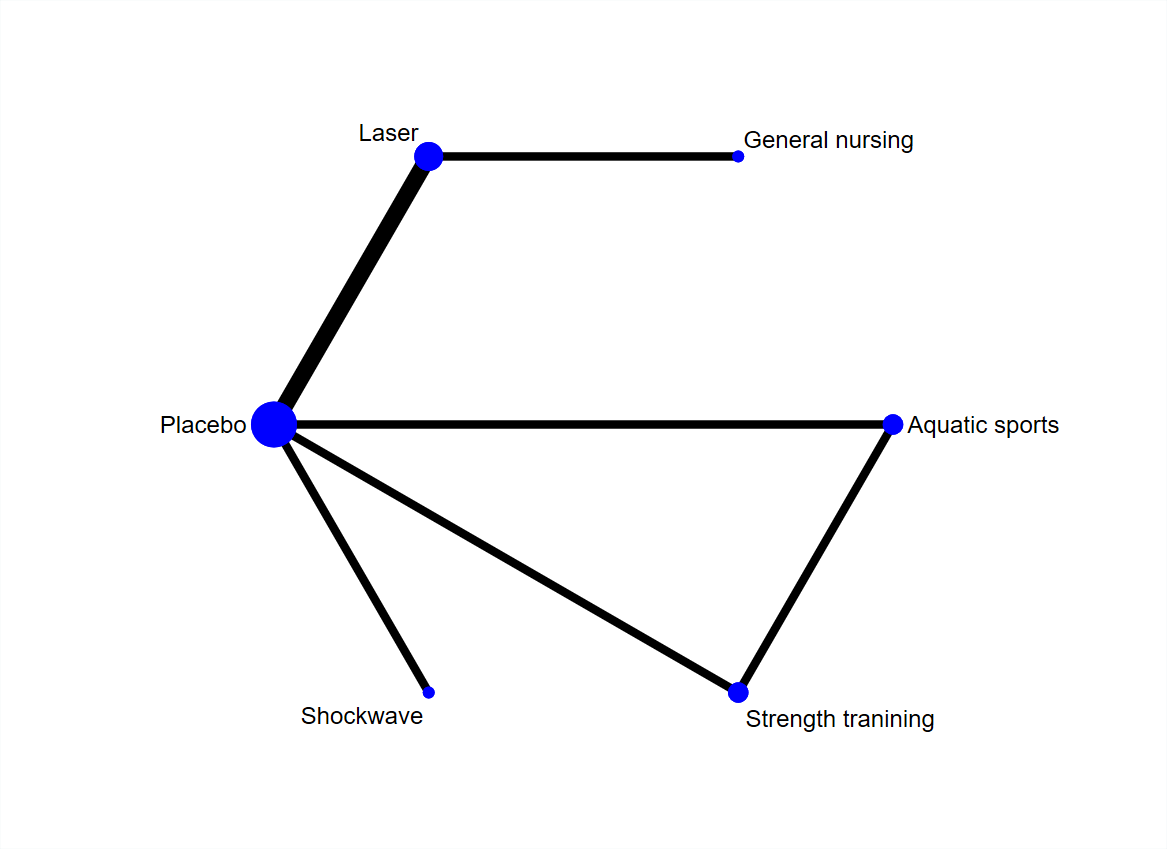


**Figure S10.15:**Cumulative ranking curve map


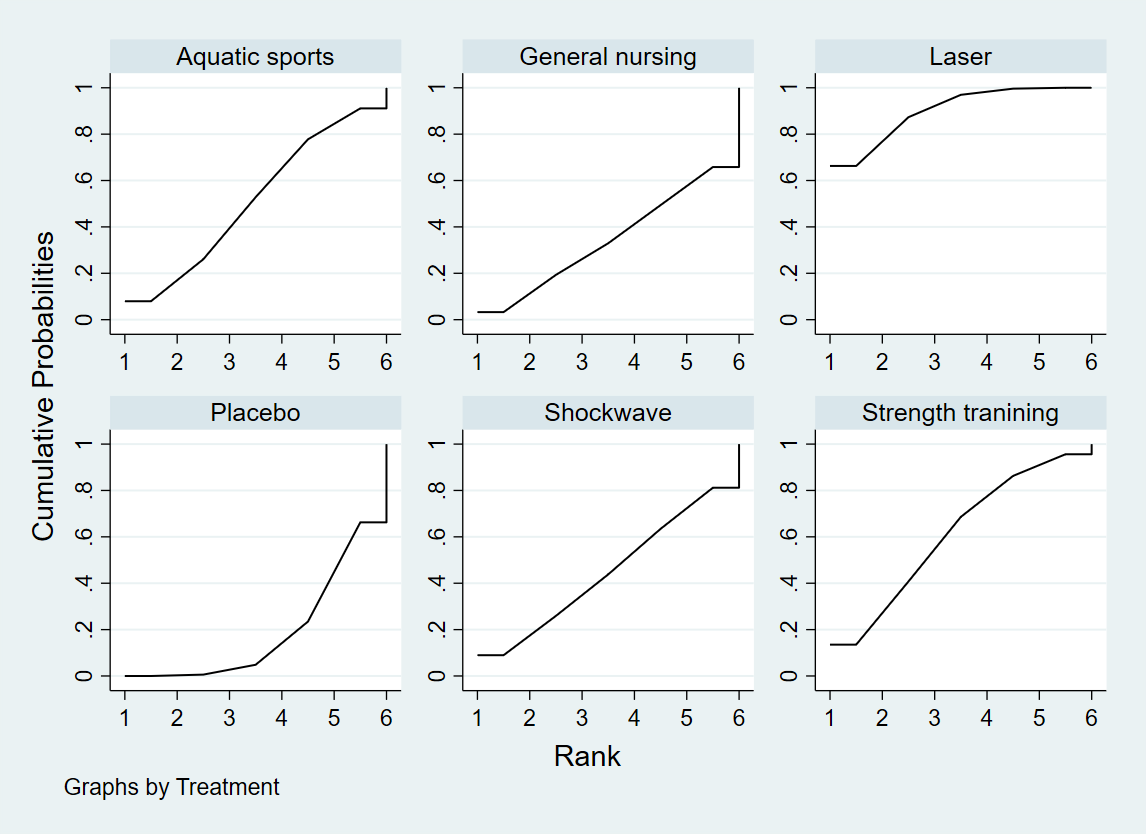


**Figure S10.16:**forest map


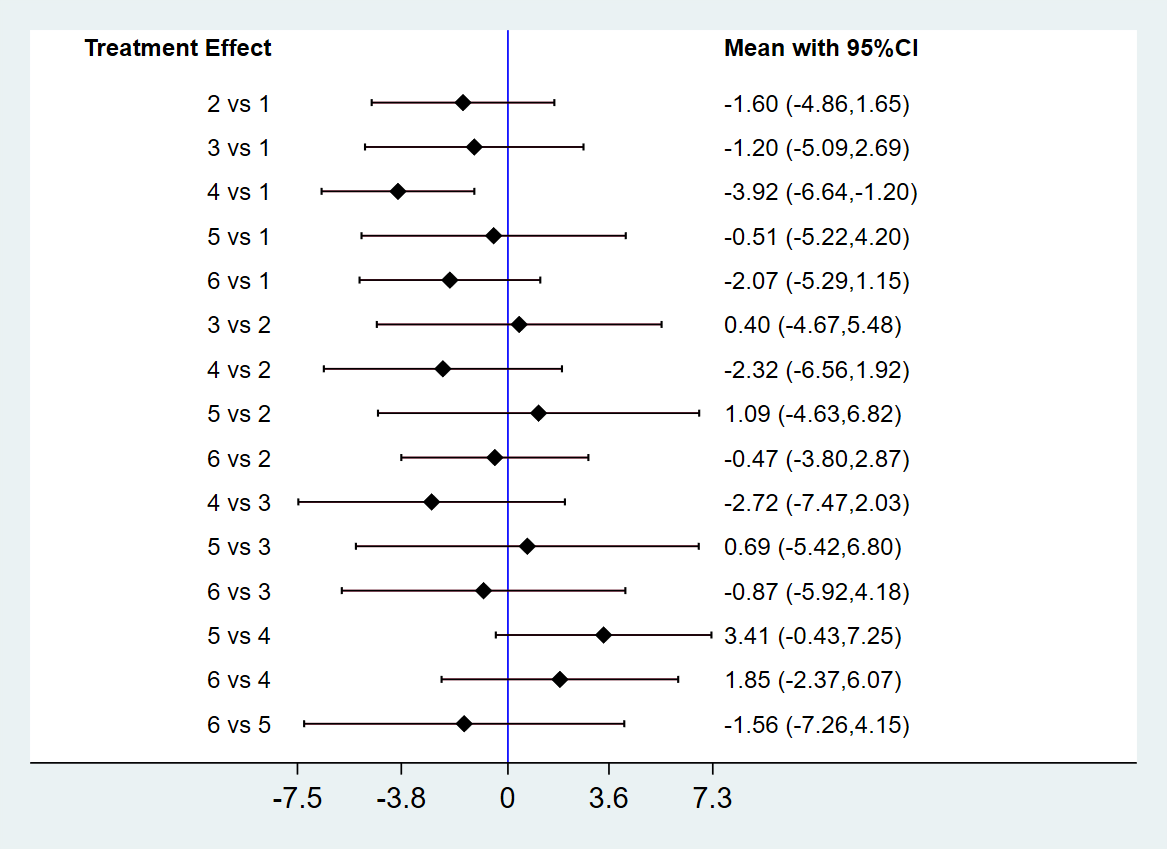


Sensitivity analysis after excluding electro-modalities

**Figure S10.17:**network map of VAS pain during walking at <1 month


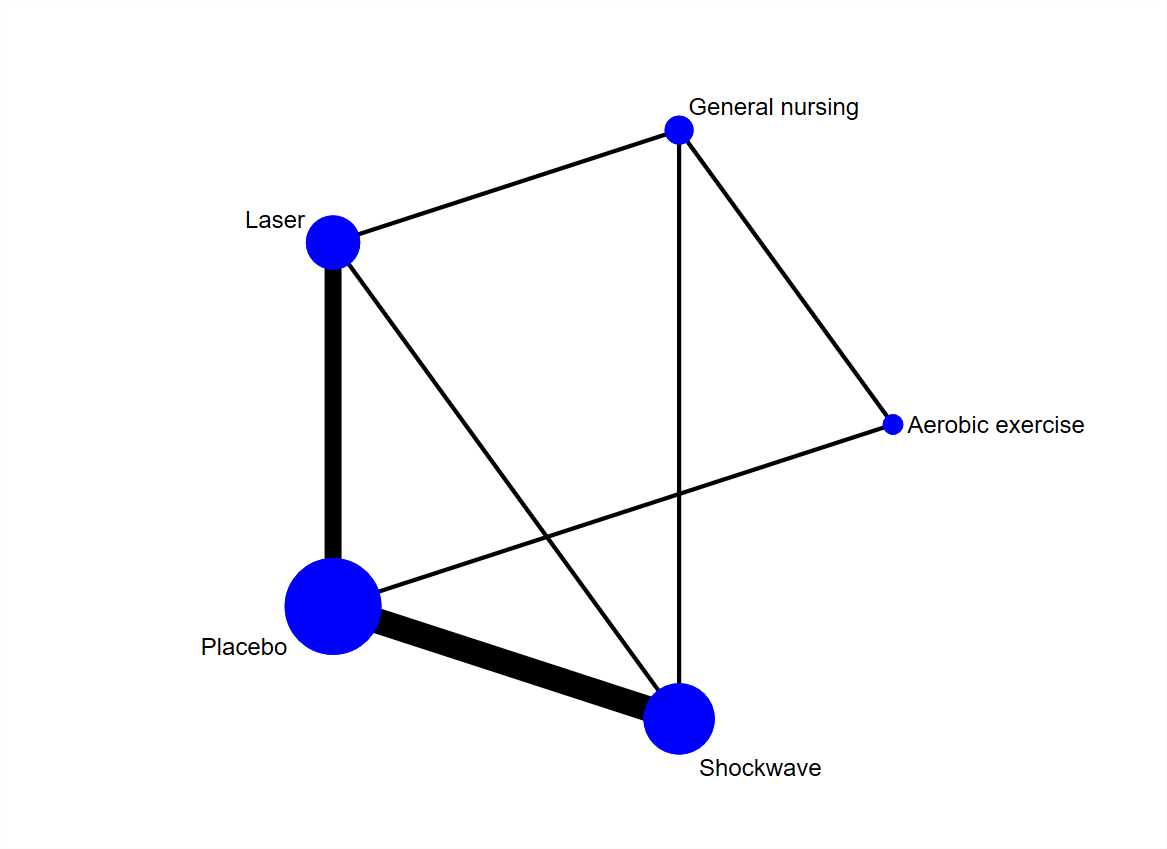


**Figure S10.18:**forest map of VAS pain during walking at <1 month


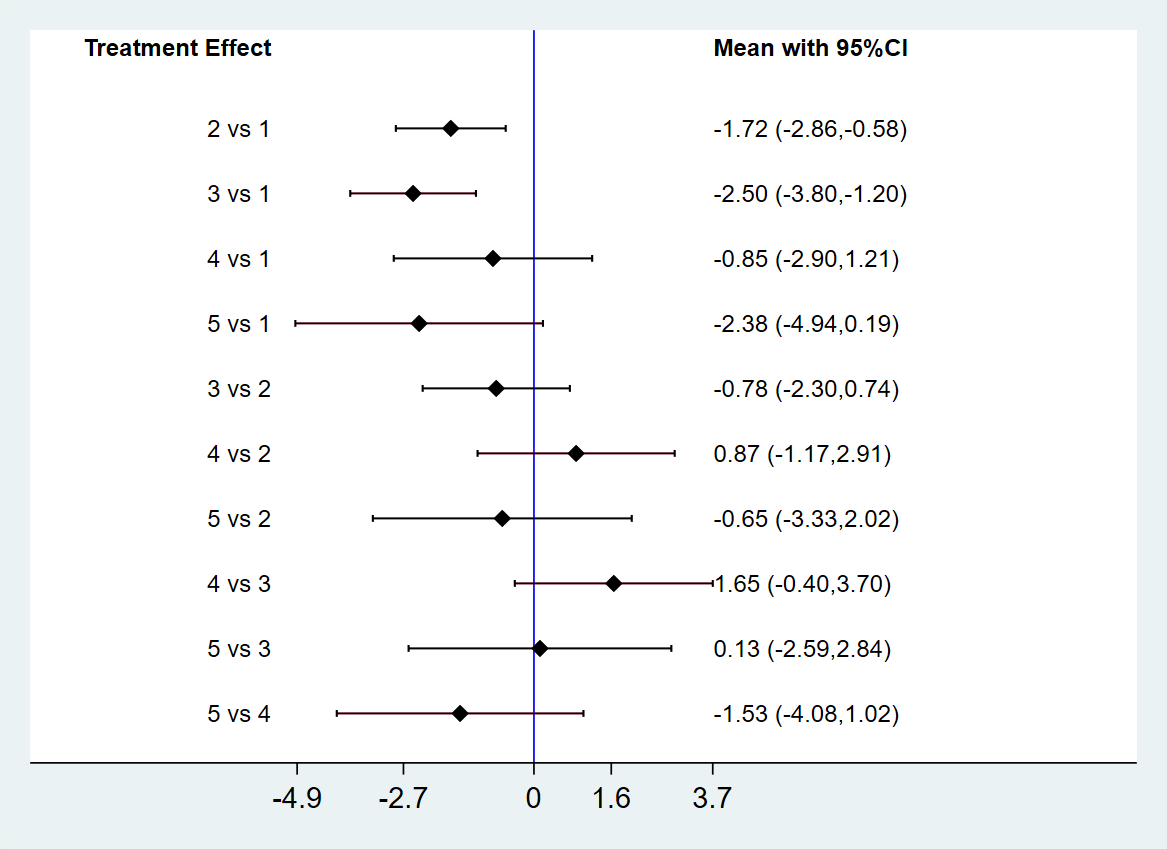


1,Placebo; 2,Shockwave; 3,Laser; 4,General nursing; 5,Aerobic exercise.

**Figure S10.19:**Cumulative ranking curve map of VAS pain during walking at <1 month


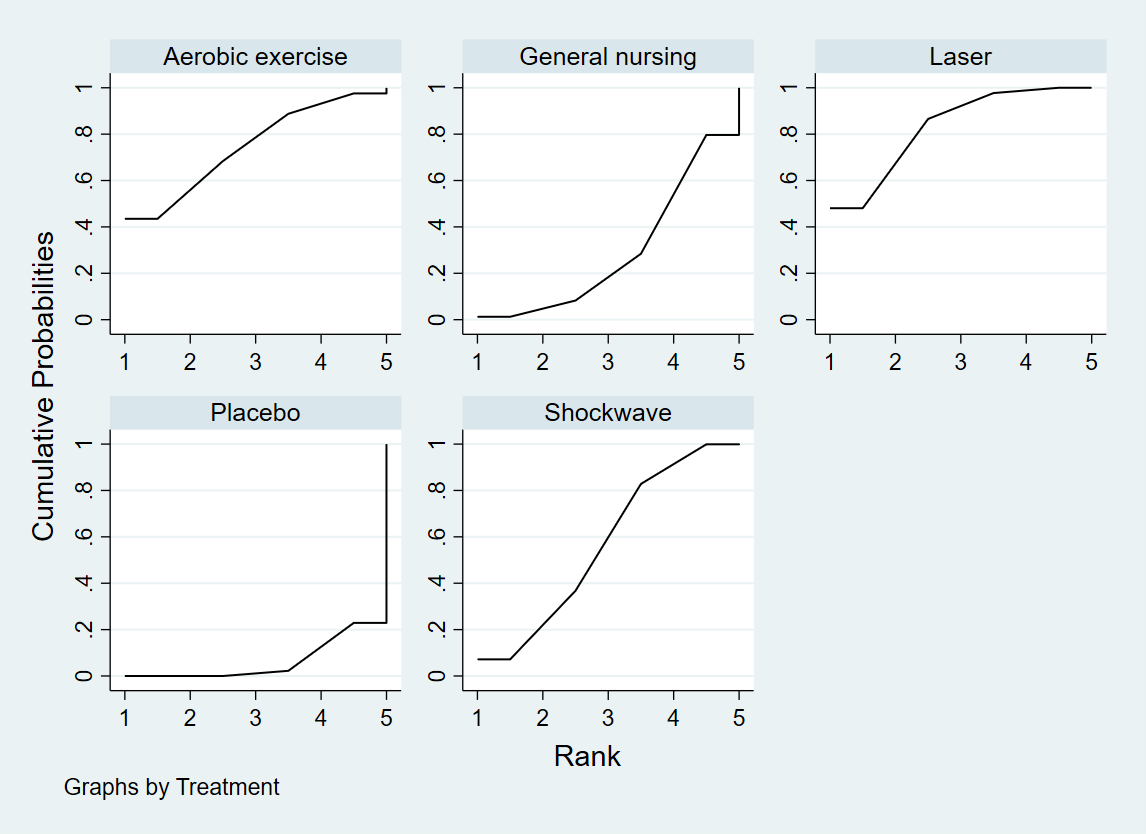


**Figure S10.20:**network map of VAS pain during walking at 1-3 months


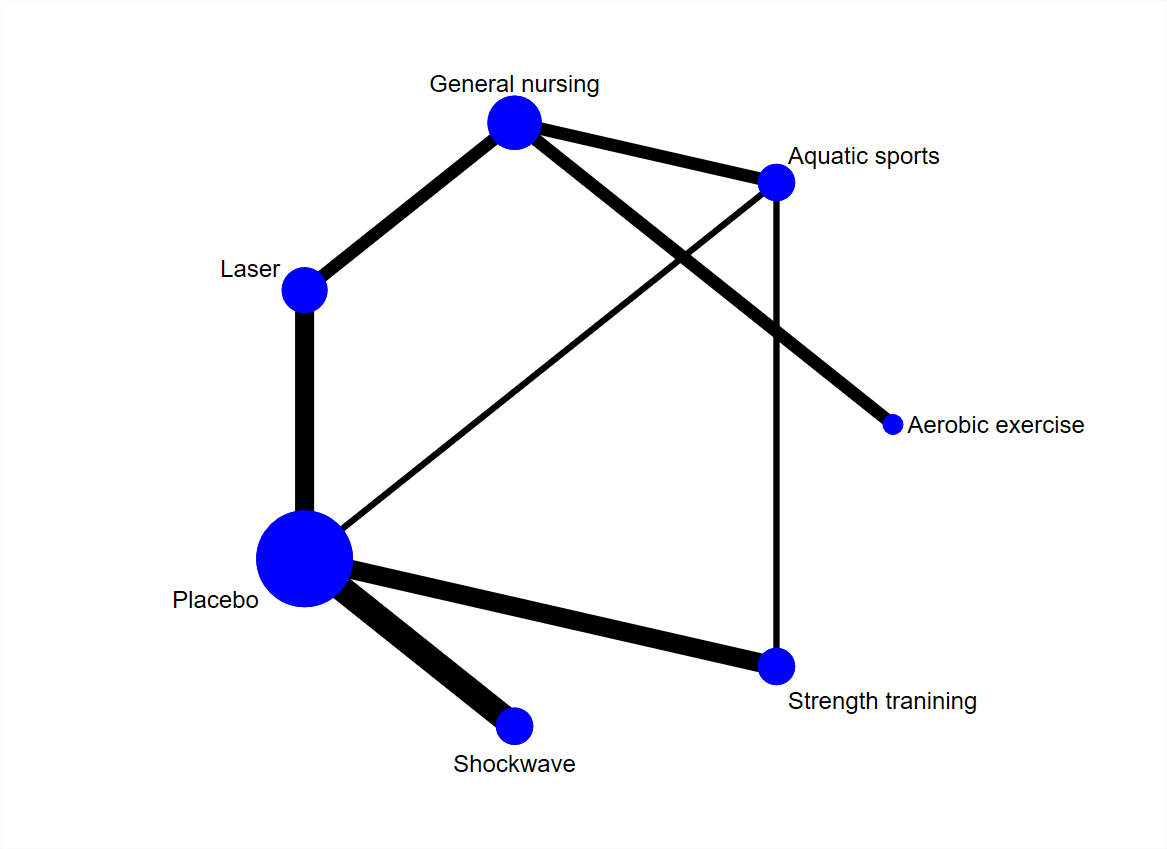


**Figure S10.21:**forest map of VAS pain during walking at 1-3 months


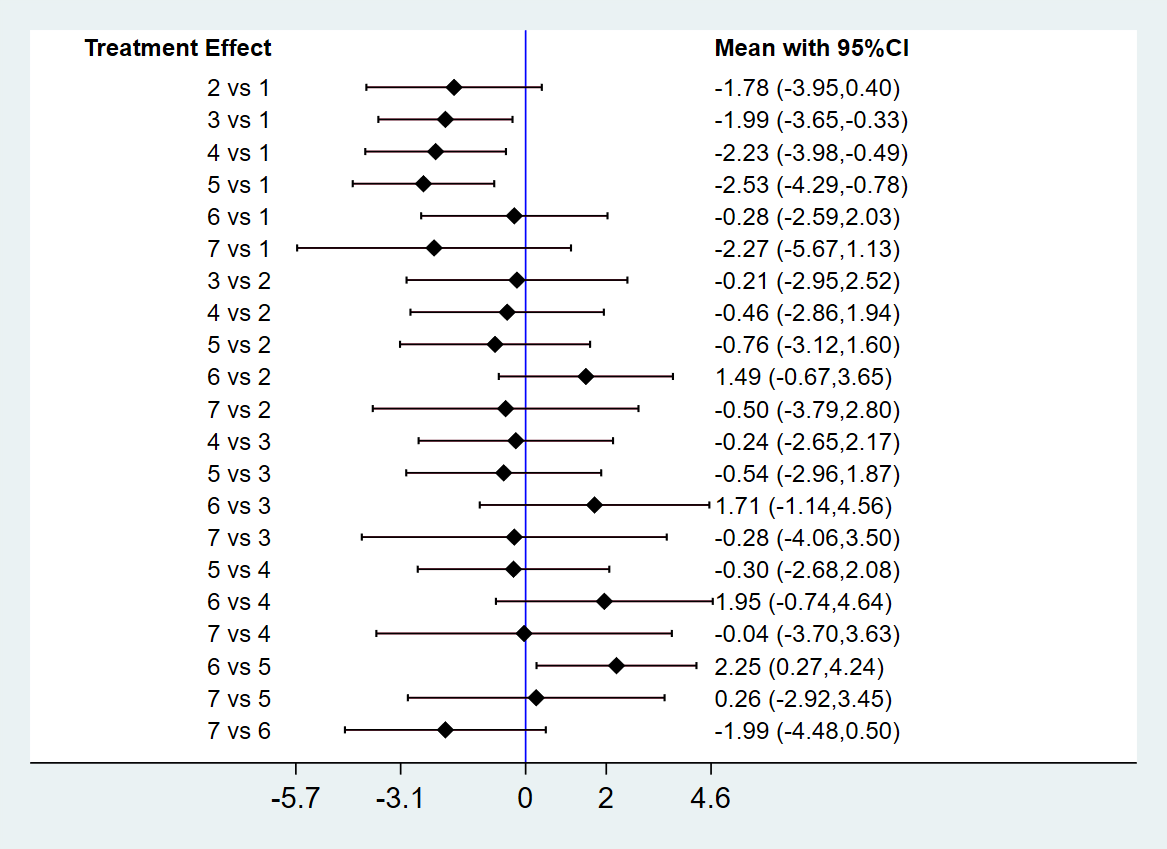


1,Placebo; 2,Aquatic sports; 3,Shockwave; 4,Strength tranining; 5,Laser; 6,General nursing; 7,Aerobic exercise.

**Figure S10.22:**Cumulative ranking curve map of VAS pain during walking at 1-3 months


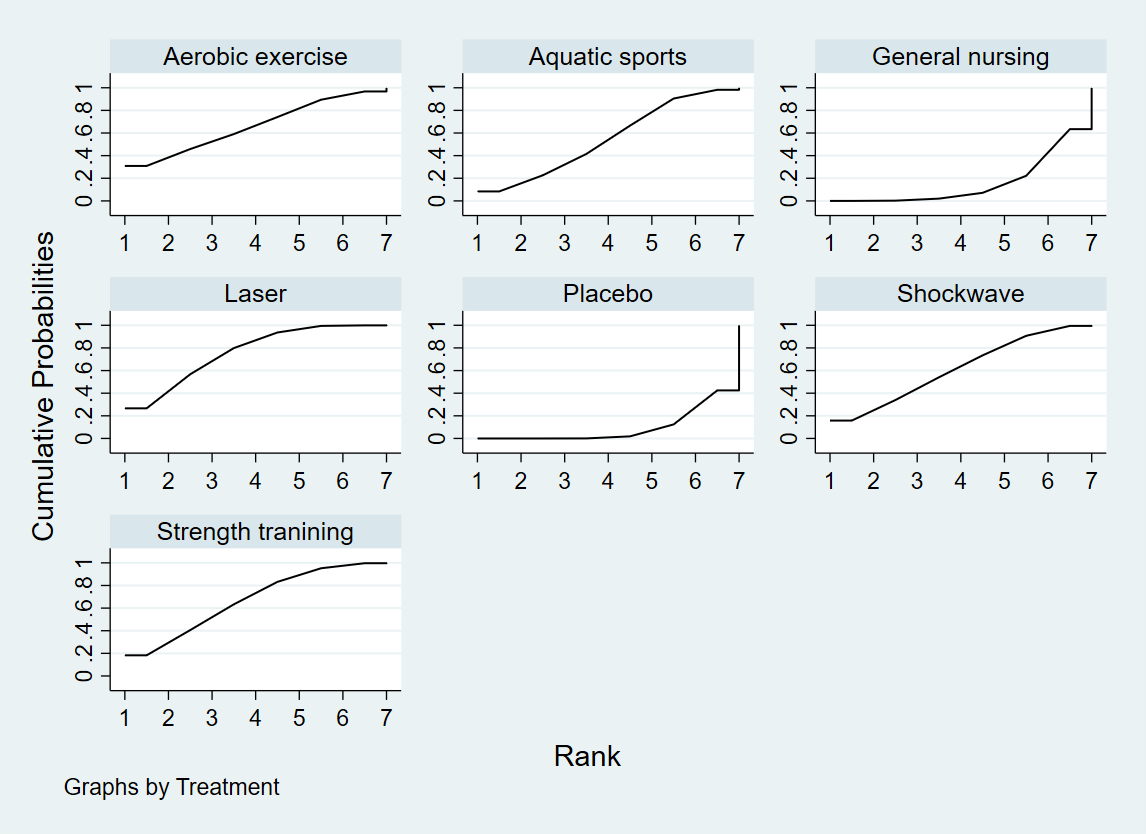


**Figure S10.23:**Network map of WOMAC total scores at <1 month


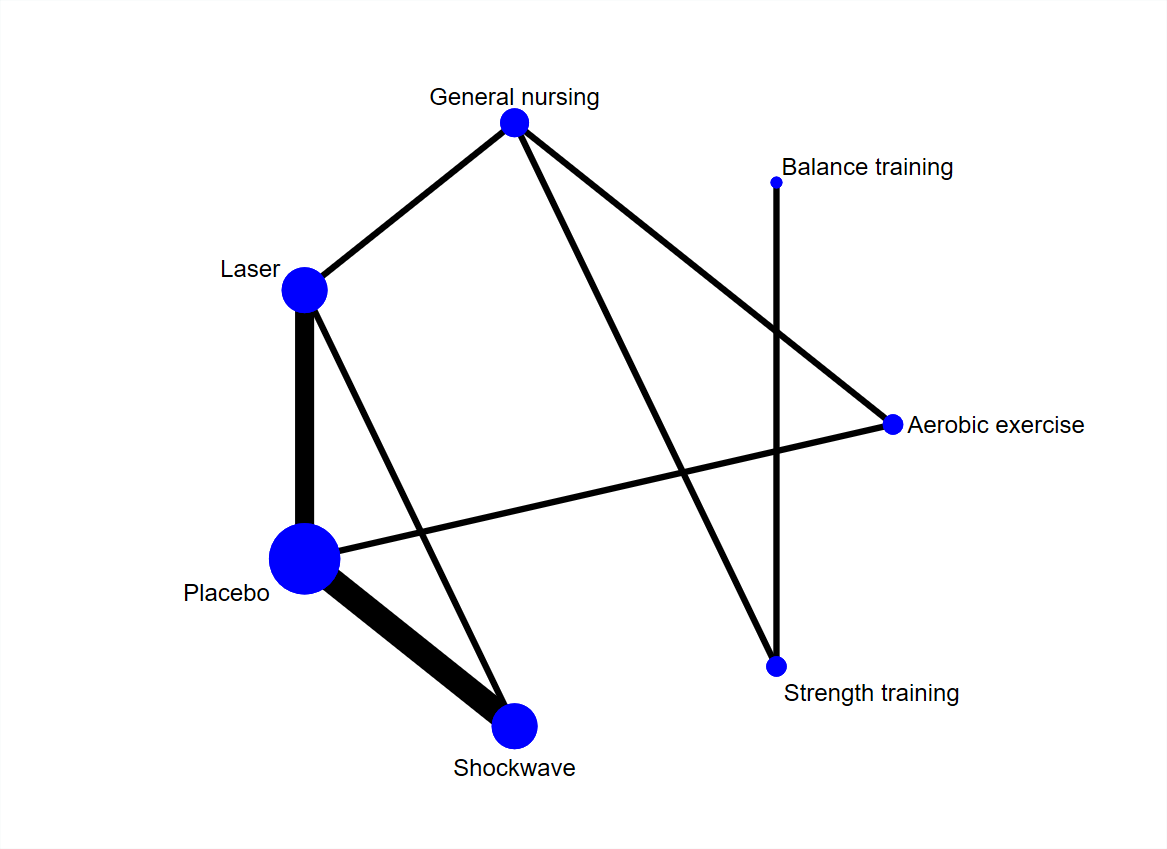


**Figure S10.24:**forest map of WOMAC total scores at <1 month


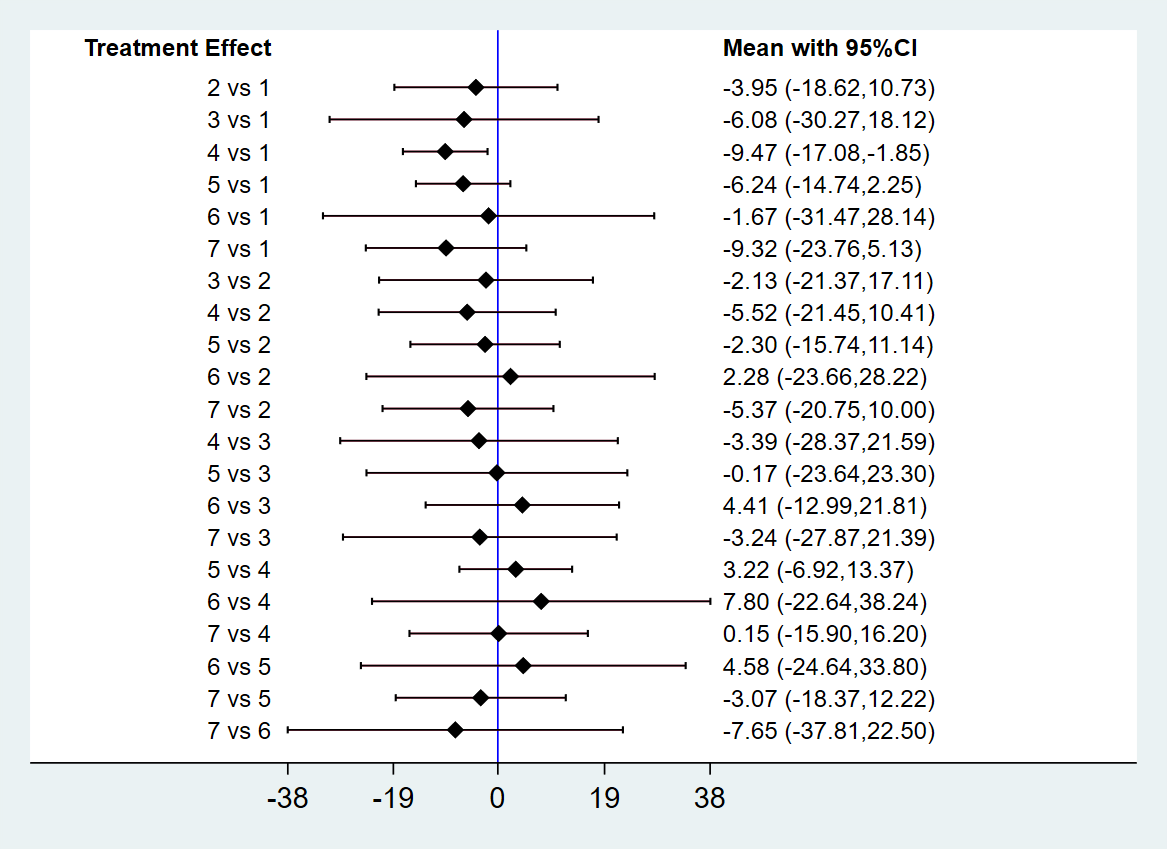


1,Placebo; 2,General nursing; 3,Strength training; 4,Shockwave; 5,Laser; 6,Balance training; 7,Aerobic exercise.

**Figure S10.25:**Cumulative ranking curve map of WOMAC total scores at <1 month
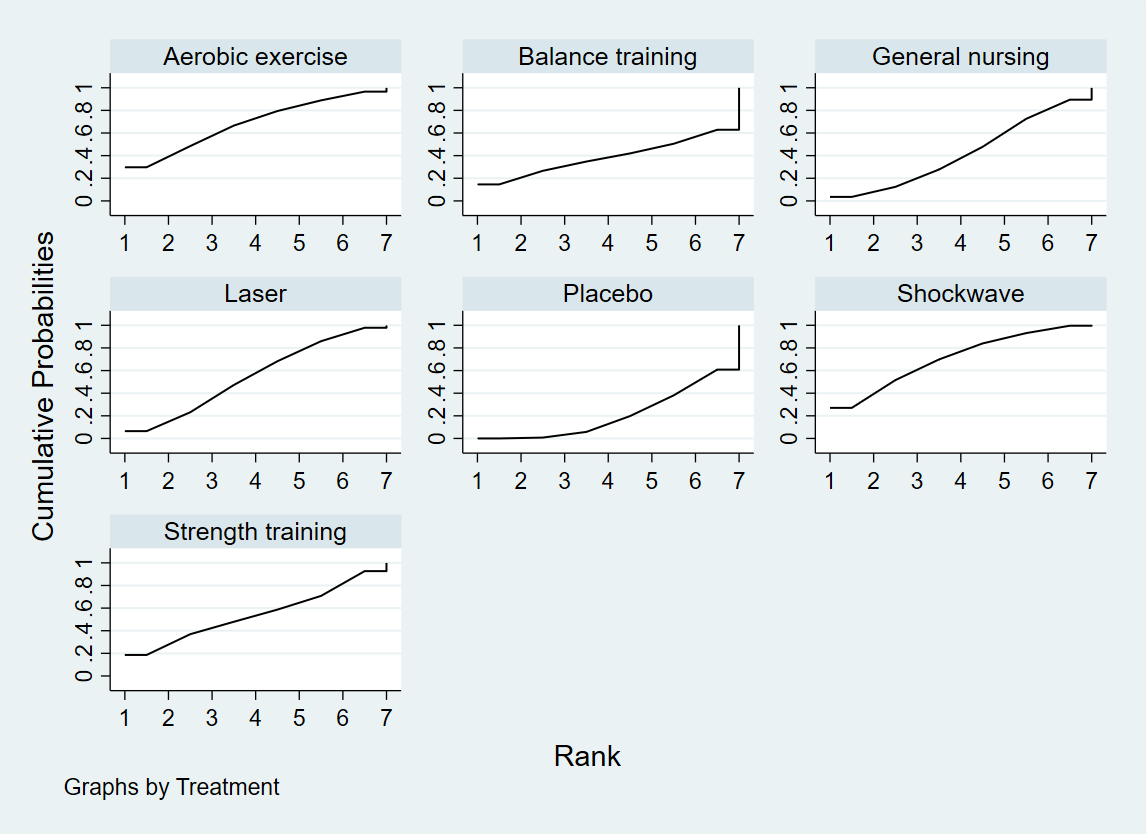


**Figure S10.26:**network map of WOMAC total scores at 1-3 months
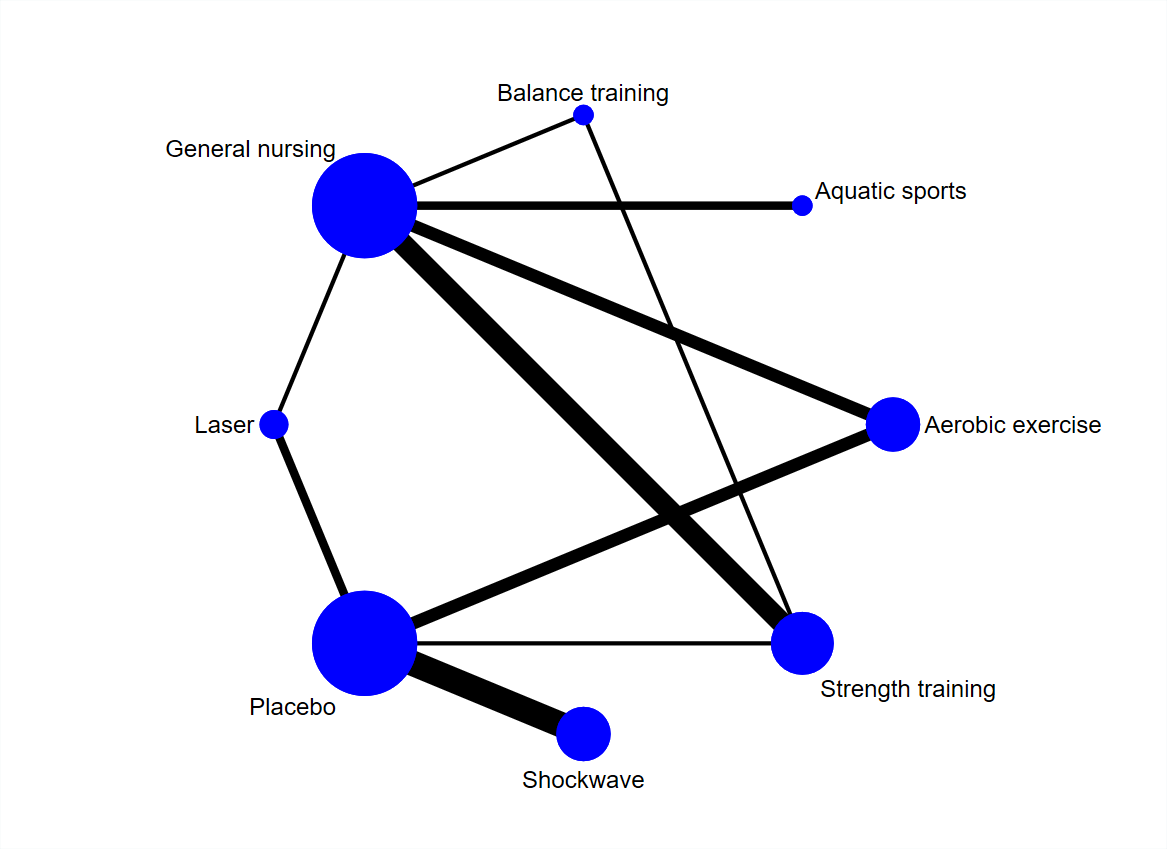


**Figure S10.27:**forest map of WOMAC total scores at 1-3 months


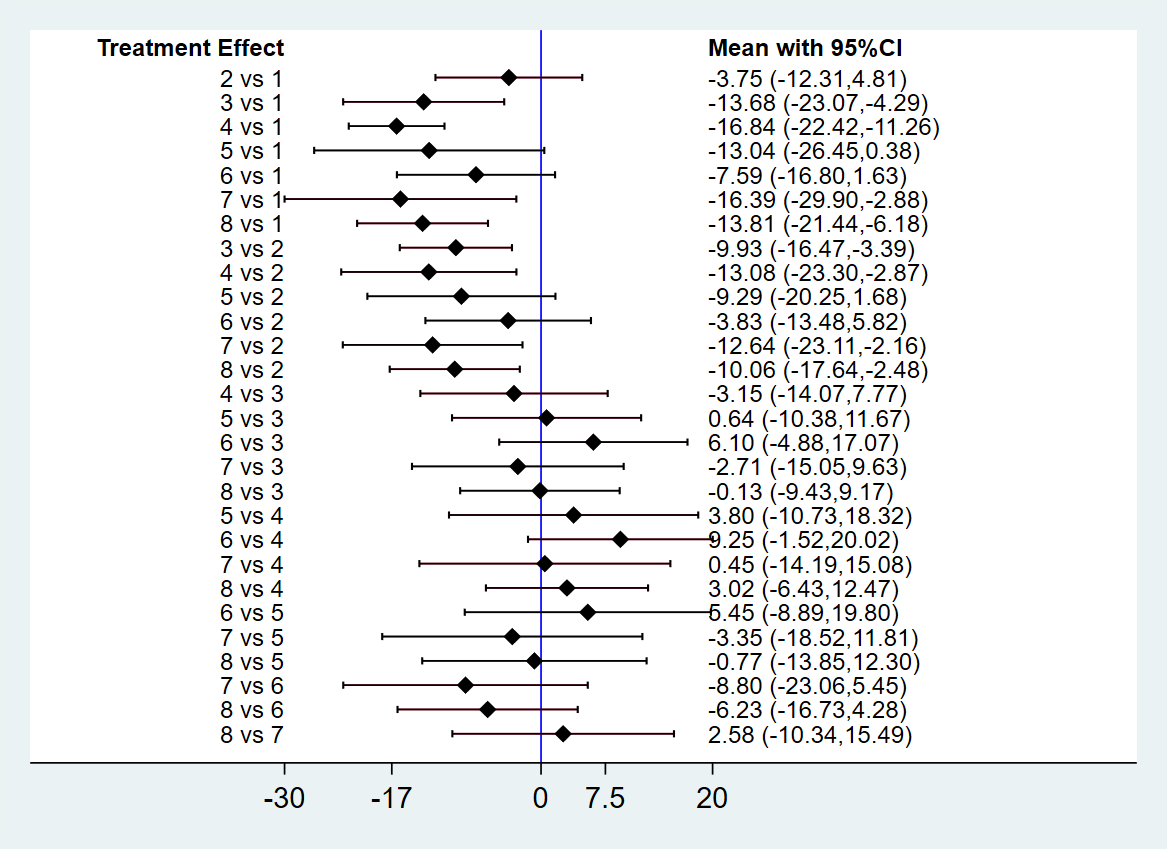


1,Placebo; 2,General nursing; 3,Strength training; 4,Shockwave; 5,Balance training; 6,Laser; 7,Aquatic sports; 8,Aerobic exercise.

**Figure S10.28:**Cumulative ranking curve map of WOMAC total scores at 1-3 months


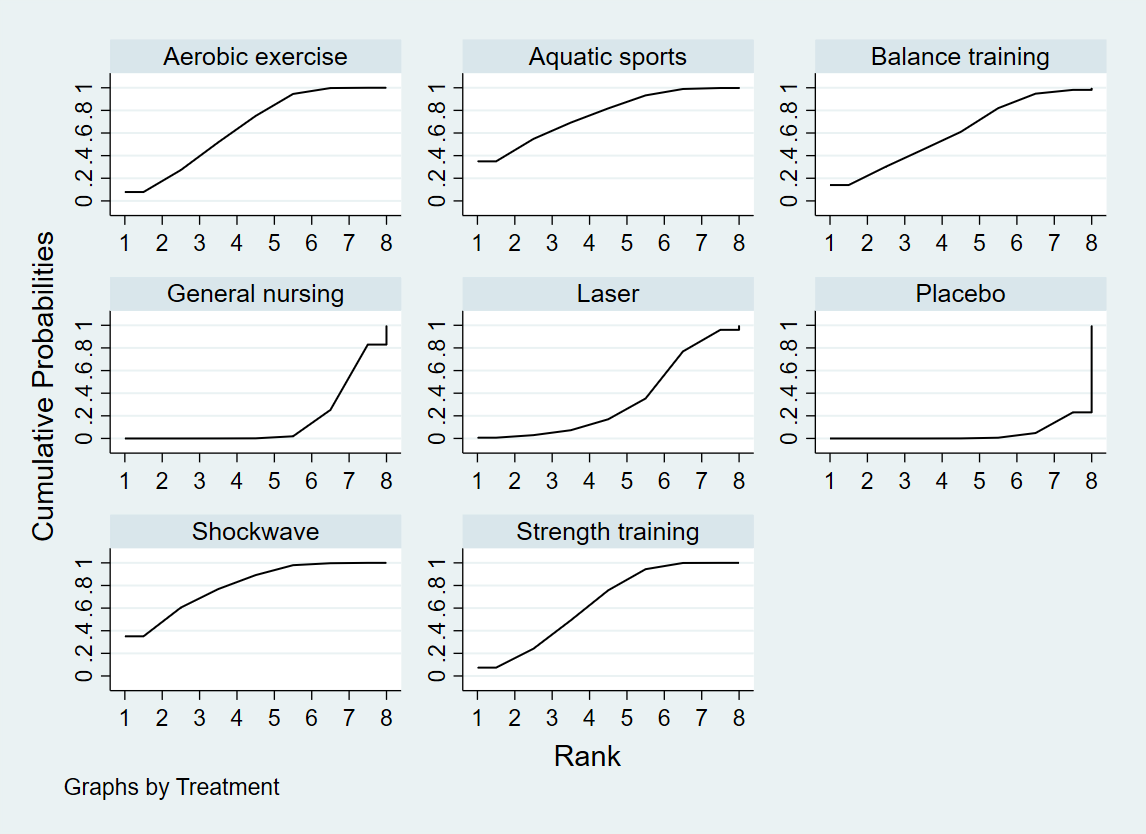


**Figure S10.29:**network map of WOMAC total scores at >3 months


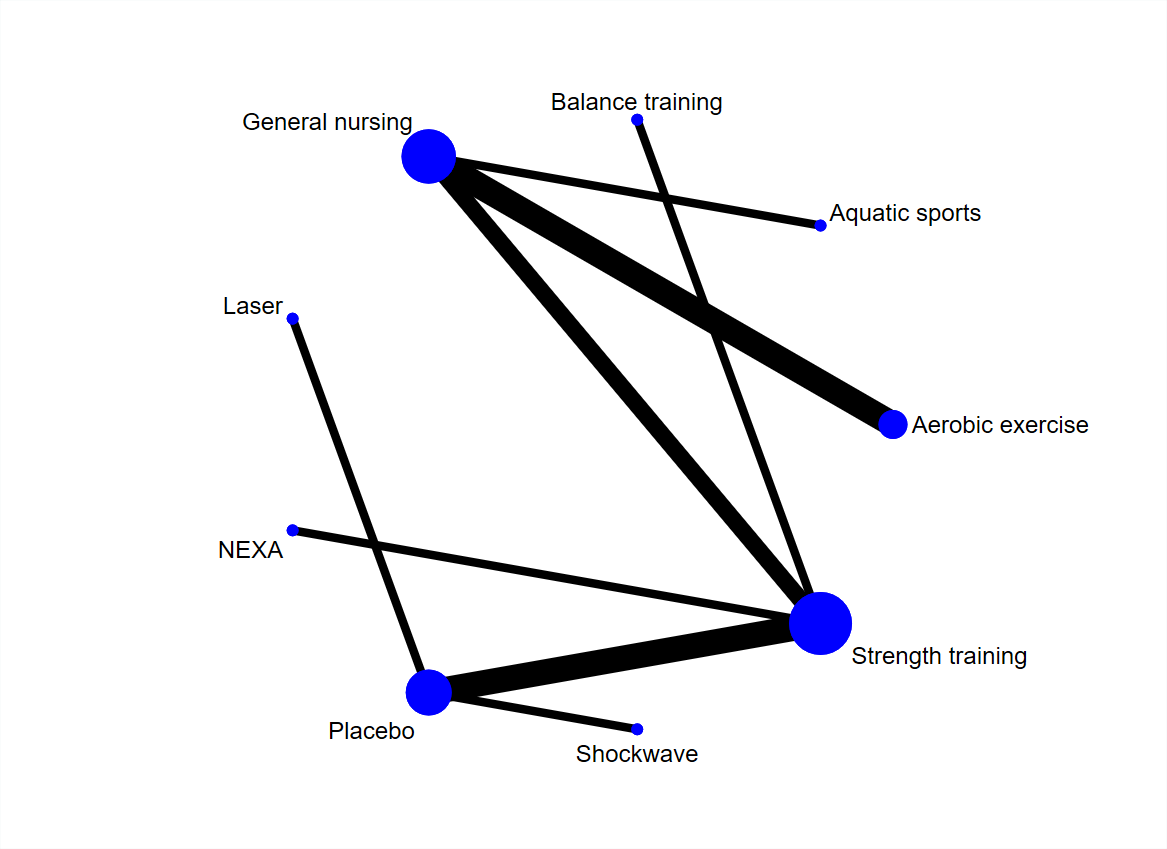


**Figure S10.30:**forest map of WOMAC total scores at >3 months


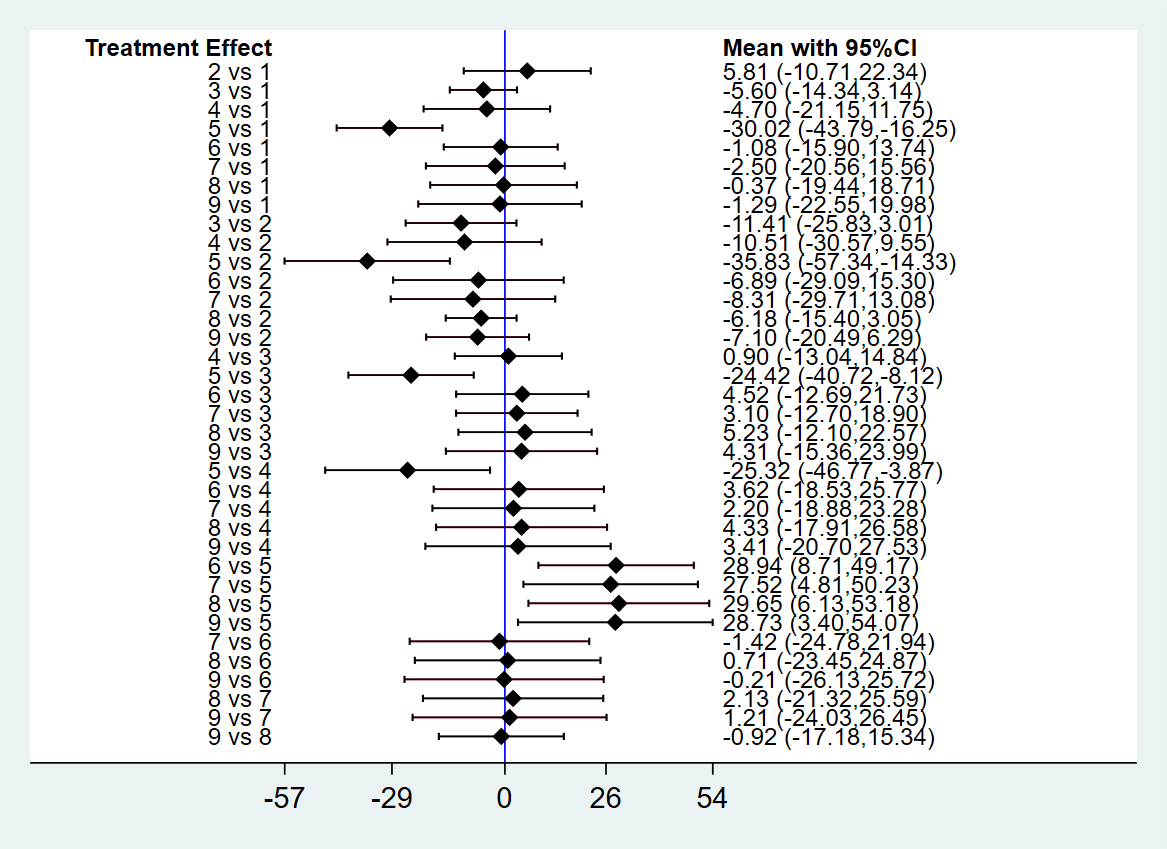


1,Placebo; 2,General nursing; 3,Strength training; 4,NEXA; 5,Shockwave; 6,Laser; 7,Balance training; 8,Aerobic exercise; 9,Aquatic sports.

**Figure S10.31:**Cumulative ranking curve map of WOMAC total scores at >3 months


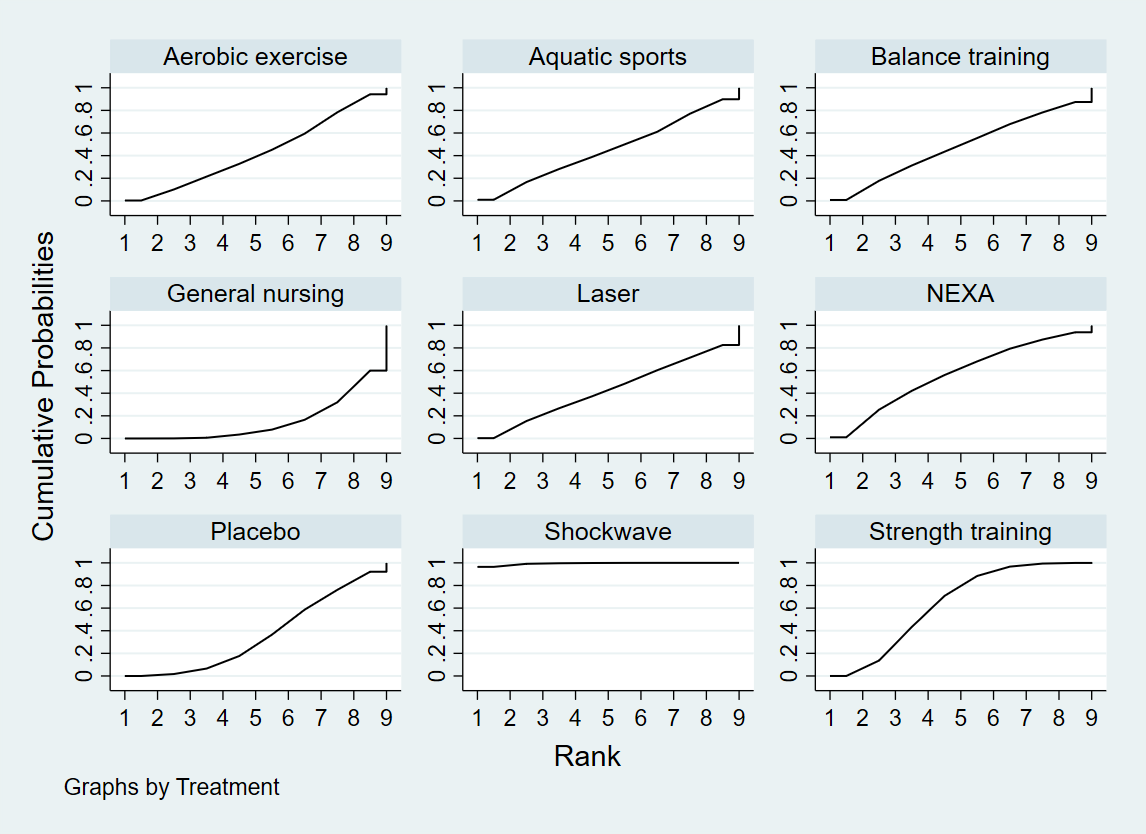


**Appendix 11: The results of subgroup analyse**

A subgroup analysis was conducted based on age, gender, and BMI. Each outcome variable consisted of two subgroups. Group A and Group B. Group A represents the lower 50% of age, gender, and BMI, while Group B represents the upper 50%. Each subgroup contains at least 10 RCTs.

For VAS pain on walking for 1-3 months, we only conducted subgroup analysis on age.

Abbreviations: NEXA, Neuromuscular exercise; TENS, Transcutaneous Electrical Nerve Stimulation.

**Figure S11.1:** Pairwise comparison of forest map of VAS pain during walking at 1-3 months Group A.


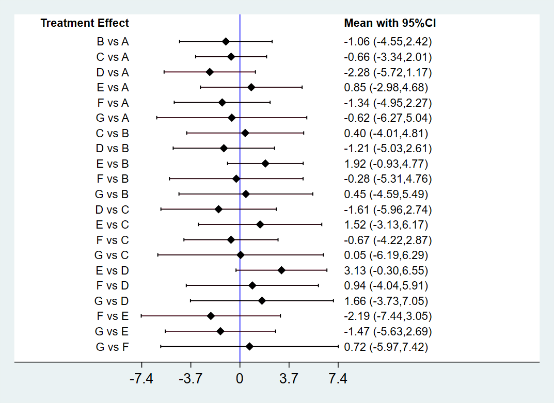


Abbreviations: A, Placebo; B, Aquatic sports; C, Shockwave; D, Laser; E, General nursing;

F, Transcutaneous Electrical Nerve Stimulation; G, Aerobic exercise.

**Figure S11.2:** Pairwise comparison of forest map of VAS pain during walking at 1-3 months Group B.


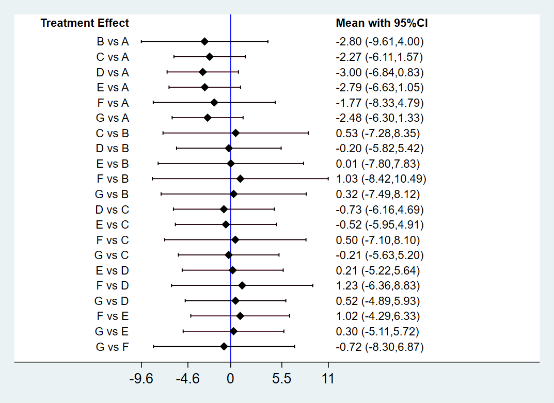


Abbreviations: A, Placebo; B, Aquatic sports; C, Shockwave; D, Strength training; E, Laser;

F, General nursing; G, Transcutaneous Electrical Nerve Stimulation.

Subgroup analysis on age for WOMAC pain1-3 months

**Figure S11.3:** Pairwise comparison of forest map of WOMAC pain 1-3 months Group A.

Abbreviations: A, Placebo; B, General nursing; C, Strength training; D, Shockwave; E, Laser; F, Aquatic sports; G, Aerobic exercise; H, Transcutaneous Electrical Nerve Stimulation.

**Figure S11.4:** Pairwise comparison of forest map of WOMAC pain 1-3 months Group B.

Abbreviations: A, Placebo; B, General nursing; C, Strength training; D, Balance training;

E, Shockwave; F, Laser; G, Aerobic exercise; H, Transcutaneous Electrical Nerve Stimulation.

Subgroup analysis on BMI for WOMAC pain1-3 months

**Figure S11.5:** Pairwise comparison of forest map of WOMAC pain 1-3 months Group A.

Abbreviations: A, Placebo; B, General nursing; C, Strength training; D, Balance training;

E, Shockwave; F, Laser; G, Aerobic exercise; H, Transcutaneous Electrical Nerve Stimulation.

**Figure S11.6:** Pairwise comparison of forest map of WOMAC pain 1-3 months Group B.

Abbreviations: A, Placebo; B, General nursing; C, Strength training; D, Shockwave; E, Laser;

F, Aquatic sports; G, Aerobic exercise; H, Transcutaneous Electrical Nerve Stimulation.

Subgroup analysis on sex for WOMAC pain1-3 months

**Figure S11.7:** Pairwise comparison of forest map of WOMAC pain 1-3 months Group A.

Abbreviations: A, Placebo; B, General nursing; C, Strength training; D, Balance training;

E, Shockwave; F, Laser; G, Aerobic exercise; H, Transcutaneous Electrical Nerve Stimulation.

**Figure S11.8:** Pairwise comparison of forest map of WOMAC pain 1-3 months Group B.

Abbreviations: A, Placebo; B, General nursing; C, Strength training; D, Balance training;

E, Shockwave; F, Laser; G, Aquatic sports; H, Aerobic exercise.

Subgroup analysis on age for WOMAC stiffness 1-3 months

**Figure S11.9:** Pairwise comparison of forest map of WOMAC stiffness 1-3 months Group A.

Abbreviations: A, Placebo; B, General nursing; C, Strength training; D, Shockwave; E, Aquatic sports; F, Aerobic exercise; G, Transcutaneous Electrical Nerve Stimulation.

**Figure S11.10:** Pairwise comparison of forest map of WOMAC stiffness 1-3 months Group B.

Abbreviations: A, Placebo; B, General nursing; C, Strength training; D, Balance training; E, Shockwave; F, Laser; G, Aquatic sports; H, Aerobic exercise; I, Transcutaneous Electrical Nerve Stimulation.

Subgroup analysis on BMI for WOMAC stiffness 1-3 months

**Figure S11.11:** Pairwise comparison of forest map of WOMAC stiffness 1-3 months Group A.

Abbreviations: A, Placebo; B, General nursing; C, Strength training; D, Shockwave; E, Laser;

F, Aquatic sports; G, Aerobic exercise; H, Transcutaneous Electrical Nerve Stimulation.

**Figure S11.12:** Pairwise comparison of forest map of WOMAC stiffness 1-3 months Group B.

Abbreviations: A, Placebo; B, General nursing; C, Strength training; D, Balance training;

E, Shockwave; F, Laser; G, Aerobic exercise; H, Transcutaneous Electrical Nerve Stimulation.

Subgroup analysis on age for WOMAC function 1-3 months

**Figure S11.13:** Pairwise comparison of forest map of WOMAC function 1-3 months Group A.

Abbreviations: A, Placebo; B, General nursing; C, Strength training; D, Balance training;

E, Shockwave; F, Laser; G, Aquatic sports; H, Aerobic exercise; I, Transcutaneous Electrical Nerve Stimulation.

**Figure S11.14:** Pairwise comparison of forest map of WOMAC function 1-3 months Group B.

Abbreviations: A, Placebo; B, General nursing; C, Strength training; D, Balance training;

E, Shockwave; F, Laser; G, Aerobic exercise; H, Transcutaneous Electrical Nerve Stimulation.

Subgroup analysis on BMI for WOMAC function 1-3 months

**Figure S11.15:** Pairwise comparison of forest map of WOMAC function 1-3 months Group A.

Abbreviations: A, Placebo; B, General nursing; C, Strength training; D, Balance training;

E, Shockwave; F, Laser; G, Aquatic sports; H, Aerobic exercise; I, Transcutaneous Electrical Nerve Stimulation.

**Figure S11.16:** Pairwise comparison of forest map of WOMAC function 1-3 months Group B.

Abbreviations: A, Placebo; B, General nursing; C, Strength training; D, Balance training;

E, Shockwave; F, Laser; G, Aerobic exercise; H, Transcutaneous Electrical Nerve Stimulation.

Subgroup analysis on sex for WOMAC function 1-3 months

**Figure S11.17:** Pairwise comparison of forest map of WOMAC function 1-3 months Group A.

Abbreviations: A, Placebo; B, General nursing; C, Strength training; D, Balance training;

E, Shockwave; F, Laser; G, Aquatic sports.

**Figure S11.18:** Pairwise comparison of forest map of WOMAC function 1-3 months Group B.

Abbreviations: A, Placebo; B, General nursing; C, Strength training; D, Balance training;

E, Shockwave; F, Laser; G, Aquatic sports; H, Aerobic exercise.

Subgroup analysis on age for WOMAC total score 1-3 months

**Figure S11.19:** Pairwise comparison of forest map of WOMAC total score 1-3 months Group A.

Abbreviations: A, Placebo; B, General nursing; C, Strength training; D, Shockwave; E; Laser;

F, Aquatic sports; G, Aerobic exercise.

**Figure S11.20:** Pairwise comparison of forest map of WOMAC total score 1-3 months Group B.

Abbreviations: A, Placebo; B, General nursing; C, Strength training; D, Shockwave; E; Balance training; F, Laser; G, Aquatic sports; H, Aerobic exercise; I, Transcutaneous Electrical Nerve Stimulation.

Subgroup analysis on BMI for WOMAC total score 1-3 months

**Figure S11.21:** Pairwise comparison of forest map of WOMAC total score 1-3 months Group A.

Abbreviations: A, Placebo; B, General nursing; C, Strength training; D, Shockwave; E; Balance training; F, Laser; G, Aquatic sports; H, Aerobic exercise.

**Figure S11.22:** Pairwise comparison of forest map of WOMAC total score 1-3 months Group B.

Abbreviations: A, Placebo; B, General nursing; C, Strength training; D, Shockwave; E; Balance training; F, Laser; G, Aerobic exercise; H, Transcutaneous Electrical Nerve Stimulation.

**Appendix 12: Adverse events**

**TableS12.1:** Summary of adverse events

| Study | Group | Adverse events | Number |
| --- | --- | --- | --- |
| Messier  2021^2^ | Strength training | Pain, fall | 9 |
|  | General nursing |  | 0 |
| Bennell  2014^4^ | Strength training | Pain, swelling | 10 |
|  | NEXA | Pain, swelling | 14 |
| Karakaş2020^9^ | Aquatic sports | Pain | 3 |
|  | Placebo |  | 0 |
| Sadeghi  2023^10^ | Shockwave |  | 0 |
|  | Placebo | Pain | 2 |
| McIlroy  2017^13^ | Shockwave | Pain | 1 |
|  | Placebo | Pain | 3 |
|  |  |  |  |
| Özgönenel2018^28^ | TENS | Transient local skin reactions | 2 |
|  | Placebo | Transient local skin reactions | 1 |
| Mascarin  2012^31^ | TENS | Subcutaneous bleeding, pins and needles, nausea | 22 |
|  | Placebo | Subcutaneous bleeding, pins and needles, | 11 |
| Pazit2018^42^ | Strength training | Pain | 1 |
|  | Placebo | Pain | 1 |
| Kuş2023^43^ | Strength training | Pain | 3 |
|  | Placebo |  | 0 |
| Takacs2017^52^ | Strength training | Pain | 1 |
|  | Placebo | Pain | 1 |
| GIN2017^53^ | Shockwave |  |  |
|  | Placebo | Pain | 2 |
| Gomiero  2018^63^ | Strength training | Pain | 3 |
|  | Placebo |  | 0 |
| Gundog M  2012^65^ | Strength training |  | 0 |
|  | Balance training | Pain | 1 |
| ELGENDY  2022^71^ | TENS | Subcutaneous hematoma, post-pinprick pain, and abdominal pain | 18 |
|  | Placebo | Subcutaneous hematoma, post-pinprick pain, and abdominal pain | 17 |
| Hammam  2020^20^ | TENS | Worsening of symptoms | 1 |
|  | Placebo | Worsening of symptoms | 1 |

**Figure S12.2:** Network map of adverse events.

**Figure S12.3:** Cumulative ranking curve plots of adverse events.

**Figure S12.4:** Pairwise comparison of adverse events.

Abbreviations: A, General nursing; B, Placebo; C, Strength training; D, neuromuscular exercise; E; Aquatic sports; F, Shockwave; G, Transcutaneous Electrical Nerve Stimulation; H, Balance training.

**Figure S12.5:** Funnel plot of adverse events.

Abbreviations: A, General nursing; B, Placebo; C, Strength training; D, neuromuscular exercise; E; Aquatic sports; F, Shockwave; G, Transcutaneous Electrical Nerve Stimulation; H, Balance training.

**Appendix 13:** **contribution matrix**

**Figure S13.1:** contribution matrix of VAS pain during walking at <1 month.

A: Aerobic exercise

B: General nursing

C: Laser

D: Placebo

E: Shockwave

F: TENS

**Figure S13.2:** contribution matrix of VAS pain during walking at 1-3 months.

A : Aerobic exercise

B: Aquatic sports

C: General nursing

D: Laser

E: Placebo

F: Shockwave

G: Strength tranining

H: TENS

**Figure S13.3:** contribution matrix of VAS pain during walking at >3 months.

A : Aerobic exercise

B: Aquatic sports

C: Balance training

D: General nursing

E: Laser

F: NEXA

G: Placebo

H: Strength training

I: TENS

**Figure S13.4:** contribution matrix of VAS pain at rest.

A: Aquatic sports

B: General nursing

C: Laser

D: Placebo

E: Shockwave

F: TENS

**Figure S13.5:** contribution matrix of NRS.

A: Balance training

B: Laser

C: Placebo

D: Shockwave

E: TENS

**Figure S13.6:** contribution matrix of WOMAC pain at <1 month.

A: Aerobic exercise

B: General nursing

C: Laser

D: Placebo

E: Shockwave

F: Strength training

G: TENS

**Figure S13.7:** contribution matrix of WOMAC pain at 1-3 months.

A : Aerobic exercise

B: Aquatic sports

C: Balance training

D: General nursing

E: Laser

F: Placebo

G: Shockwave

H: Strength training

I: TENS

**Figure S13.8:** contribution matrix of WOMAC pain at >3 months.

A : Placebo

B: General nursing

C: Strength training

D: NEXA

E: Laser

F: TENS

G: Aerobic exercise

H: Aquatic sports

**Figure S13.9:** contribution matrix of WOMAC stiffness scores at <1 month.

A: Aerobic exercise

B: General nursing

C: Laser

D: Placebo

E: Shockwave

F: Strength training

G: TENS

**Figure S13.10:** contribution matrix of WOMAC stiffness scores at 1-3 months.

A: Aerobic exercise

B: Aquatic sports

C: Balance training

D: General nursing

E: Laser

F: Placebo

G: Shockwave

H: Strength training

I: TENS

**Figure S13.11:** contribution matrix of WOMAC stiffness scores at >3 months.

A : Aerobic exercise

B: General nursing

C: Laser

D: NEXA

E: Placebo

F: Strength training

G: TENS

**Figure S13.12:** contribution matrix of WOMAC function scores at <1 month.

A : Aerobic exercise

B: General nursing

C: Laser

D: Placebo

E: Shockwave

F: Strength training

G: TENS

**Figure S13.13:** contribution matrix of WOMAC function scores at 1-3 months.

A: Aerobic exercise

B: Aquatic sports

C: Balance training

D: General nursing

E: Laser

F: Placebo

G: Shockwave

H: Strength training

I: TENS

**Figure S13.14:** contribution matrix of WOMAC function scores at >3 months.

A: Aerobic exercise

B: General nursing

C: Laser

D: NEXA

E: Placebo

F: Strength training

G: TENS

**Figure S13.15:** contribution matrix of WOMAC total scores at <1 month.

A: Aerobic exercise

B: Balance training

C: General nursing

D: Laser

E: Placebo

F: Shockwave

G: Strength training

H: TENS

**Figure S13.16:** contribution matrix of WOMAC total scores at 1-3 months.

A: Aerobic exercise

B: Aquatic sports

C: Balance training

D: General nursing

E: Laser

F: Placebo

G: Shockwave

H: Strength training

I: TENS

**Figure S13.17:** contribution matrix of WOMAC total scores at >3 months.

A: Aerobic exercise

B: Aquatic sports

C: Balance training

D: General nursing

E: Laser

F: NEXA

G: Placebo

H: Shockwave

I: Strength training

J: TENS
